# Upcycling Silicon as Heterogeneous Palladium Catalysts: Heck–Cassar Cross‐Coupling in Batch and Flow Conditions

**DOI:** 10.1002/cssc.70827

**Published:** 2026-06-27

**Authors:** Tian Sang, Giulia Brufani, Tommaso Scarabottini, Sofiya Zabelinskaya, Dmitri Gelman, Francesco Mauriello, Luigi Vaccaro

**Affiliations:** ^1^ Laboratory of Green S.O.C. – Dipartimento di Chimica Biologia e Biotecnologie Università degli Studi di Perugia Perugia Italy; ^2^ Department of Civil, Energy, Environmental and Material Engineering (DICEAM) Università degli Studi Mediterranea di Reggio Calabria Reggio Calabria Italy; ^3^ Institute of Chemistry The Hebrew University Jerusalem Israel

**Keywords:** continuous flow, Heck–Cassar cross‐coupling, heterogeneous catalyst, palladium nanoparticles, silicon

## Abstract

Electronic waste and end‐of‐life photovoltaic modules are positioning silicon as a high‐value material that will increasingly enter the waste stream in the coming years. Strategies to valorize this valuable waste are therefore highly desirable. Among heterogeneous catalysts, SiO_2_ plays a prominent role both as a catalyst and as a support for metal nanoparticles. Research activity exploring the use of silicon in catalysis demonstrates different applications in energetic and material chemistry. Given the growing interest in developing materials derived from industrial waste, biomass, and e‐waste as supports for heterogeneous catalysts within a circular economy framework, this work aims to demonstrate the effective upcycling of silicon by developing a Pd/Si‐based catalytic system. The resulting catalyst is designed for continuous‐flow cross‐coupling applications, exemplified by the Heck–Cassar reaction, also known as the Cu‐free Sonogashira coupling, and demonstrates both robustness and recyclability. Its applicability is demonstrated in the Heck–Cassar reaction across 21 different substrates, with yields ranging from 63% to 99%.

## Graphical abstract

Silicon application as a support for Pd Nanoparticle catalysts in Heck–Cassar cross‐coupling under continuous flow.

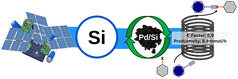

## 1 Introduction

Silicon is the second most abundant element in Earth’s crust, after oxygen, and occurs primarily as silicates [1]; it is a key element of modern technological development. It plays a crucial role in the production of electronic devices, advanced materials, and photovoltaic panels. New technologies and the affordability of electronics drive the annual generation of waste electrical and electronic equipment (WEEE) [2, 3]. Estimating the exact annual volume of e‐waste is challenging. Nevertheless, the European Union generates an estimated 10 million tons of end‐of‐life electronic devices annually [4]. This waste stream constitutes a significant reservoir of economic value: in 2019, the global market for secondary electronic resources was valued at approximately USD 57 billion [5]. The usability of valuable materials present in these waste streams supports the development of renewable energy technologies, helping to overcome both the scarcity of raw materials and the adverse effects associated with the use of non‐renewable energy resources [6]. Metals such as copper, nickel, and palladium can be recovered using hydrometallurgical and pyrometallurgical techniques and subsequently converted in situ into catalytically active nanoparticles, or employed in the preparation of mixed‐metal oxide catalysts, or without the need for extensive separation steps [7]. In addition, non‐metallic components can be repurposed as catalyst supports, enhancing catalyst stability and dispersion [8]. In this context, e‐waste also serves as a source of carbon and silicon for the preparation of nanostructured materials [9].

Photovoltaic (PV) panels, counted among the WEEE, drive the global transition toward renewable energy sources. Currently, PV systems predominantly operate on a linear “take‐make‐use‐dispose” model, leading to increased landfill waste and environmental concerns. To mitigate these issues, transitioning toward circular strategies and establishing an efficient PV recycling infrastructure is essential [10]. Projections anticipate substantial global growth in solar PV production, reaching over 1630 GW by 2030 and a remarkable 4500 GW by 2050. With an average lifetime of 25–30 years, global PV waste is expected to reach 8 million tons by 2030, surging to 60–78 million tonnes by 2050. It is estimated that the cumulative PV waste generated by 2050 will have an economic value of 15 billion USD through recycling [11, 12]. The crystalline silicon (c‐Si) solar cell industry has occupied more than 90% of the PV market [10, 13]. As a result, a substantial fraction (3.65%) of the total mass of Si‐based PV panels expected to enter the waste stream consists of solar‐grade, ultra‐high‐purity (≥99.9999%), high‐cost, and energy‐intensive silicon [14, 15]. The environmental impact of crystalline silicon production is significant because the metallurgical purification process is highly energy intensive. Recycling can significantly reduce the high costs associated with solar‐grade silicon production while mitigating its adverse environmental impacts [16, 20]. However, silicon recovered from disposed PV modules is rarely reused in the solar industry, primarily due to wafer breakage and contamination (e.g., dopants, Ag/Al coatings, EVA residues) [18], resulting in a metallurgical‐grade purity (~99%) [14, 15]. This limitation has prompted the exploration of alternative strategies to valorize this high‐value waste [21, 29]. In this work, we propose exploiting metallurgical‐grade silicon as a support for palladium nanoparticles and applying it as a heterogeneous catalyst.

Among heterogeneous catalysts, metal oxides in high oxidation states are well‐established, with SiO_2_ playing a particularly prominent role as both catalyst and support. SiO_2_ is an irreducible oxide and is generally regarded as a relatively inert support. Silica derived from waste finds extended applicability as mesoporous materials [30, 31] and in catalytic applications [32, 34]. Metal nanoparticles supported on silica have become a common platform for heterogeneous catalysis [35, 37]. The high density of surface silanol (Si─OH) groups strongly influences the size, dispersion, and oxidation state of supported metal nanoparticles, thereby enhancing catalytic performance and demonstrating suitability for applications under both batch and continuous‐flow apparatus [38, 40].

**FIGURE 1:**
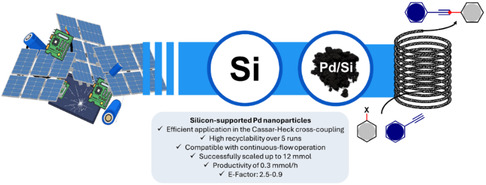
Development of silicon‐based Pd‐nanoparticle catalyst applied in the Heck–Cassar cross‐coupling reaction under continuous flow conditions.

Given the growing interest in the recycling of materials derived from industrial waste, biomass, and e‐waste as supports for heterogeneous catalysts within a circular economy framework [30, 34, 41, 47], the objective of this work is to demonstrate the effective upcycling of silicon through the development of a Pd/Si‐based catalytic system, suitable for continuous‐flow cross‐coupling applications in C─C bond formation. The Heck–Cassar reaction was selected as a benchmark method for accessing alkynyl moieties, which are key structural motifs in numerous pharmaceuticals and natural products [48, 51]. The Pd/Si catalyst was explored as a platform for developing a waste‐minimized Heck–Cassar cross‐coupling protocol under both batch and continuous‐flow conditions. Its performance and stability under the reaction conditions were investigated and compared with those of commercially available catalysts. Furthermore, recyclability and reusability studies were undertaken to examine catalyst robustness and to gain insight into the role of Si‐based supports in influencing catalytic stability and reactivity (Figure 1).

## 2 Results and Discussion

The heterogeneous catalyst Pd/Si is produced using the polyol method, which has already been applied to waste‐derived carbonaceous supports for the immobilization of metallic nanoparticles (NPs) [52, 54]. In a 50 mL two‐neck round‐bottom flask, 400 mg of the Si support was suspended in 20 mL of diethylene glycol. An aqueous solution of H_2_PdCl_4_ (prepared by dissolving 87 mg of PdCl_2_ in 1 mL of HCl) was added dropwise under sonication. The pH was adjusted to 12–13 by adding 5 M NaOH solution. The reduction step was carried out at 130°C for 3 h under an Ar atmosphere. The suspension was filtered and washed several times with hot water, ethanol, and acetone. The loading of Pd (2.7 wt%) was measured by MP‐AES analysis.

**FIGURE 2:**
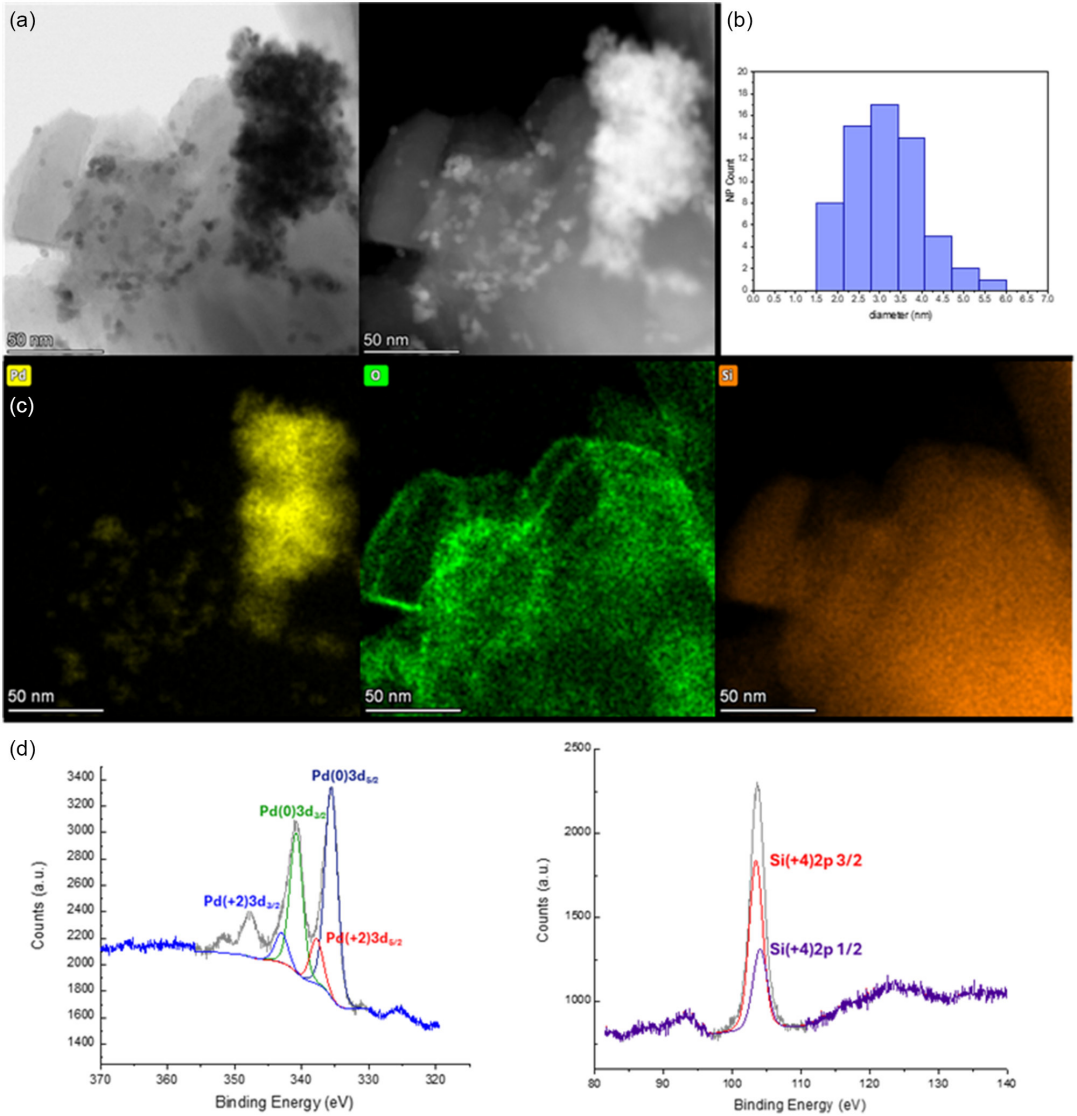
Characterization of Pd/Si: (a) STEM image of Pd/Si; (b) nanoparticle size distribution; (c) EDS elemental mapping of Pd, O, and Si; (d) XPS spectra of Pd and Si.

The Pd/Si was characterized by STEM and EDS (Figure 2a,b), revealing the formation of Pd nanoparticles (NPs) with an average particle size of 3.2 ± 1 nm (Figure 2c). XPS analysis exhibits very intense Pd 3d peaks, which are mainly composed of Pd with oxidation states (0) at a binding energy of 335.6 eV and (+2) at 337.8 eV, with a 0/+2 ratio of 5. The peaks corresponding to the Pd 3d_5/2_ and Pd 3d_3/2_ orbitals show a DSOC (doublet spin–orbit coupling) of 5.2 eV, both for Pd0 and Pd(+2). Measurements of the Si 2p region reveal only the component corresponding to oxidized silicon with an oxidation state of +4 (Figure 2d).

Optimization of the Heck–Cassar reaction was initiated using the Pd/Si catalyst to identify the most efficient reaction conditions for C─C bond formation. Starting from established reaction conditions for the Heck–Cassar coupling [53], iodobenzene **1a** and phenylacetylene **2a** were selected as model substrates using DABCO as organic base (Table 1). In line with a sustainability‐driven approach, iodobenzene was selected over bromobenzene and chlorobenzene to ensure efficient reactivity under mild conditions. Its superior reactivity enables the use of a simple, ligand‐free heterogeneous catalyst, thereby avoiding costly and waste‐generating ligand systems. This strategy reduces both environmental impact and process complexity, aligning with more sustainable and industrially relevant cross‐coupling practices [55]. Notably, when the optimized conditions were applied using chlorobenzene or bromobenzene in place of iodobenzene, no conversion was observed.

**TABLE 1:** Heck–Cassar cross‐coupling reaction conditions optimization.

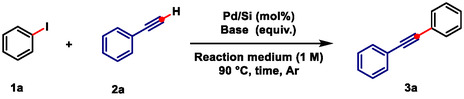						
**Entry** a	**Medium**	**Base**	**Base (equiv.)**	**Time, h**	**2a** **(equiv.)**	**C, %** b
1	EtOH	DABCO	1.5	24	1.5	43
2	PEG	DABCO	1.5	24	1.5	52
3	DMF	DABCO	1.5	24	1.5	69
4	1,4‐dioxane	DABCO	1.5	24	1.5	91
5	TAME	DABCO	1.5	24	1.5	96
6	CPME	DABCO	1.5	24	1.5	99
7^c^	CPME	DABCO	1.5	24	1.5	99
8	CPME	Et_3_N	1.5	24	1.5	10
9	CPME	DBU	1.5	24	1.5	61
10	CPME	K_2_CO_3_	1.5	24	1.5	—
11	CPME	Na_2_CO_3_	1.5	24	1.5	—
12	CPME	Cs_2_CO_3_	1.5	24	1.5	—
13^d^	CPME	DABCO	1.5	24	1.5	97
14^e^	CPME	DABCO	1.5	24	1.5	99
15^f^	CPME	DABCO	1.5	16	1.1	80
16^f^	CPME	DABCO	1	16	1	73
17^f^	CPME	DABCO	1.1	16	1.1	99
18^f^	CPME	DABCO	1.1	5	1.1	99
19^f^	CPME	DABCO	1.1	4	1.1	93
20^f^	CPME	DABCO	1.1	3	1.1	87
21^f^	CPME	DABCO	1.1	1	1.1	84
22^f^^,^ g	CPME	DABCO	1.1	3 h	1.1	76
23^f^^,^ h	CPME	DABCO	1.1	3 h	1.1	81

a Reaction conditions: Pd/Si (2.7 wt%; 0.5 mol%), **1a** (1 mmol), **2a** (1.5 equiv.), DABCO (1.5 equiv.), reaction medium (1 mL), 90°C, 24 h, under Ar.

b GC conversion has been determined using samples of pure compounds as reference standards.

c Under an air atmosphere.

d Reaction medium (2 mL).

e Reaction medium (0.5 mL).

f Reaction medium (0.1 mL).

g Pd/C (10 wt%; 0.5 mol%).

h Pd/Al_2_O_3_ (1 wt%; 0.5 mol%).

The influence of the reaction medium was explored (entries 1–6). Protic solvents, ethanol (entry 1) and PEG (entry 2), were detrimental, likely due to competitive coordination at the Pd surface or inhibition of the active state. Polar aprotic solvents such as DMF (entry 3) afforded incomplete conversion, likely due to their coordination to Pd, trapping it in poorly reactive intermediates. By contrast, ethereal reaction mediums, 1,4‐dioxane and TAME, provided effective performance (entries 4 and 5), and CPME showed comparable efficacy (entry 6). This trend can be rationalized in the context of a release‐and‐catch mechanism. The low coordinative ability of ethereal solvents toward Pd prevents the inhibition, while the reduced polarity promotes the neutral catalytic pathway and improves solvation of the organic substrates. Beyond its optimal catalytic performance, CPME offers a distinctly favorable profile: low toxicity, high chemical stability, and production from petrochemical waste *via* a 100% atom‐economy process [56, 59].

The reaction was performed under air and gave analogous results; however, to ensure reproducibility, the optimization was performed under Ar (entry 7). The efficiency of forming **3a** was evaluated using various bases in CPME at 90°C for 24 h (entries 8–12). Primarily, it can be noted that while organic bases consistently led to **1a** conversion, inorganic carbonates were ineffective due to their low solubility in CPME. Organic bases, particularly DABCO, afforded superior performance. In contrast, Et_3_N (entry 8) and DBU (entry 9) afforded inferior results. The superior performance of DABCO compared to Et_3_N is likely not exclusively related to its basicity but rather to its dual role as both a base and a transient coordinating ligand. In heterogeneous Cu‐free catalytic systems, DABCO may stabilize catalytically active metal species and facilitate acetylide formation. The amount of solvent used was screened, with no significant variation in concentration observed (entries 13–15). Then, the concentration reduction minimized the amount of base employed and **2a**, and significantly reduced the total reaction time required to fully convert the initial starting materials (entries 15–21). When the two reaction substrates **1a** and **2a** were used in a stoichiometric ratio, only a moderate conversion was observed (entry 16). Increasing the amount of **2a** to 1.1 equiv. significantly improved the outcome, delivering complete conversion under otherwise identical conditions (entry 17). Under these conditions, 5 h was identified as the minimum time to achieve quantitative conversion (entry 18), whereas shorter times resulted in slightly lower conversions (entries 19–21).

Comparative evaluation with two commercially available catalysts demonstrated that Pd/Si exhibited markedly superior catalytic activity, achieving in just 1 h a conversion comparable to that obtained with Pd/C or Pd/Al_2_O_3_ in 3 h (entries 22–23).

**FIGURE 3:**
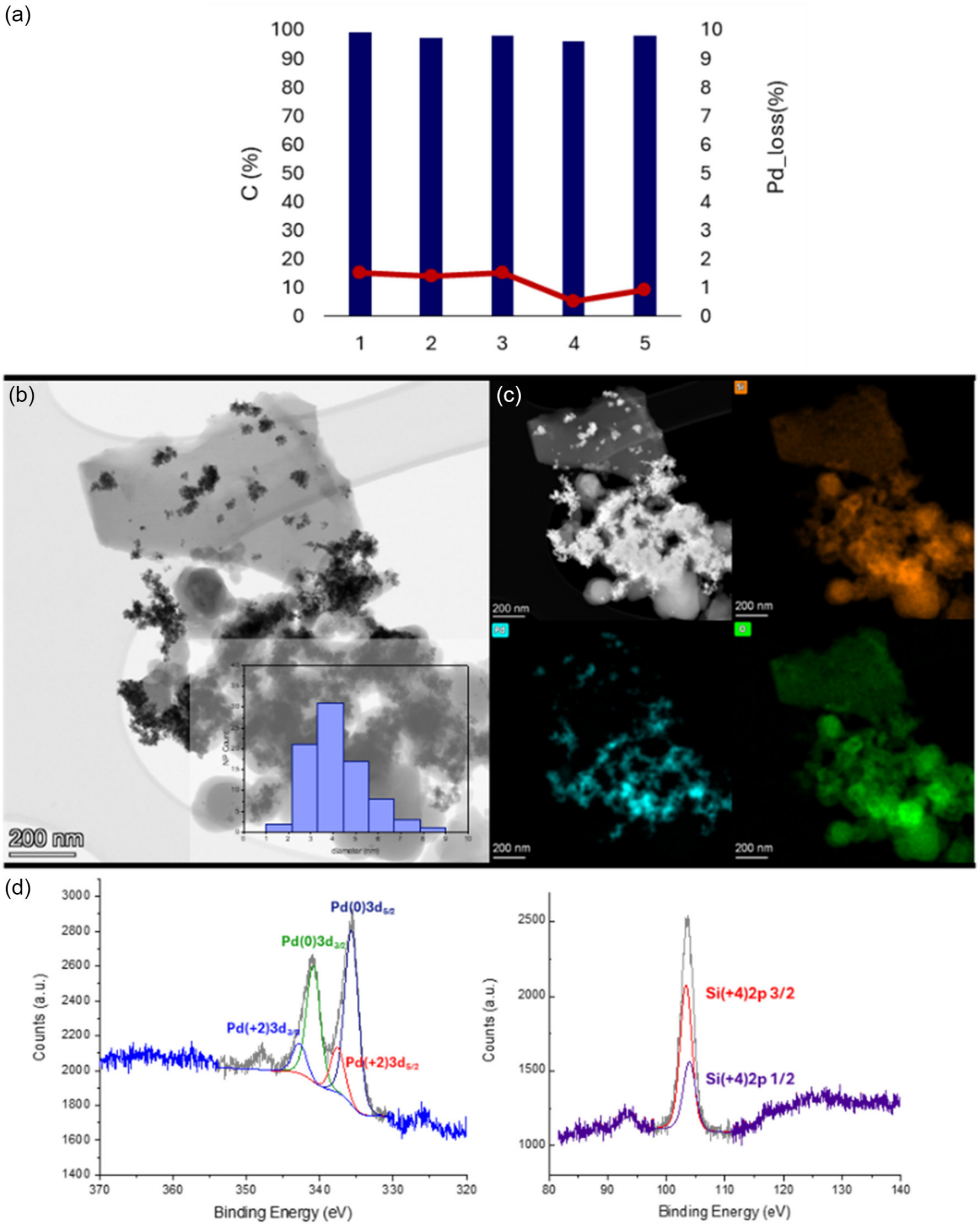
(a) Recycle of Pd/Si for 5 consecutive runs under optimized reaction conditions. (b) STEM images of the spent catalyst and Pd nanoparticle size distribution. (c) EDS images of the spent catalyst showing Si, O, and Pd. (d) XPS analysis of the spent catalyst. Reaction conditions: Pd/Si (0.5 mol%), **1a** (1 mmol), **2a** (1.1 equiv.), DABCO (1.1 equiv.), CPME (0.1 mL), 90°C, 5 h, under Ar. C (%) has been determined using samples of pure compounds as reference standards.

At this stage, the catalyst’s stability was assessed by evaluating its performance over five consecutive runs (Figure 3). The catalyst maintained consistent activity throughout the repeated cycles, with minimal palladium leaching observed at the end of each run (Figure 3a). STEM analysis of the spent catalyst confirmed that the nanometric dimensions of the Pd nanoparticles were preserved, with an average diameter of 3.1 ± 0.1 nm (Figure 3b). XPS analysis revealed that the catalyst surface is predominantly composed of Pd in the 0 oxidation state (binding energy 336.1 eV) along with Pd^2+^ (binding energy 337.7 eV), with a Pd^0^/Pd^2+^ ratio of 5. The Pd3d_5/2_ and 3d_3/2_ peaks exhibit a spin–orbit coupling (DSOC) of 5.2 eV for both oxidation states (Figure 3d).

To gain mechanistic insight into the catalytic pathway, we investigated a catch‐and‐release process using a combination of kinetic analysis, catalyst poisoning, and leaching measurements [60]. We first conducted time‐resolved kinetic studies under optimized reaction conditions, monitoring the reaction from 30 min to 5 h (See Supporting Information). The concentration‐versus‐time plot showed that the reaction reached 84% conversion within 1 h. This time was selected to perform the mercury‐drop test, observing only a marginal increase to 85% after a total of 5 h with mercury addition, implying that the palladium at this point is predominantly in a soluble form susceptible to amalgamation. To further substantiate this, a hot filtration test was carried out under identical conditions. MP‐AES analysis revealed notable Pd leaching (>4.5%) after 30 min and 1 h, consistent with partial dissolution of Pd species from the heterogeneous catalyst. Nevertheless, recycling experiments demonstrated that the catalyst consistently delivered high yields over 5 runs, with Pd leaching levels remaining low (<1.5%), indicating efficient capture of leached Pd species in the solid phase (see Supporting Information). These observations strongly suggest that catalysis proceeds at least in part *via* a dynamic catch‐and‐release mechanism, consistent with previous observations in heterogeneous Pd‐catalyzed cross‐coupling reactions.

**SCHEME 1:**
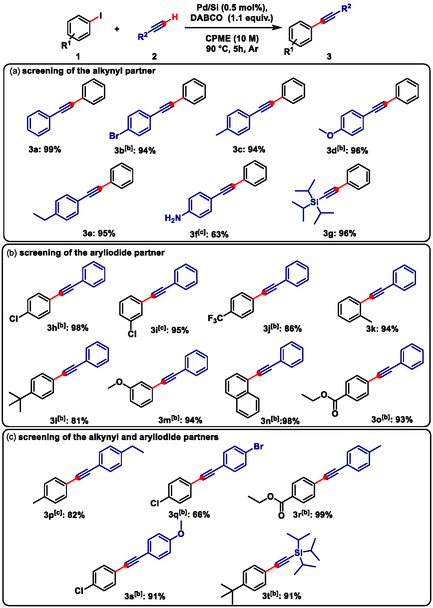
Substrate scope under optimized reaction conditions. (a) screening of alkynyl partners; (b) screening of aryl iodide partners; (c) screening of both alkynyl and aryl iodide partners. [a] Reaction conditions: Pd/Si (0.5 mol%), **1** (1 mmol), **2** (1.1 equiv.), DABCO (1.1 equiv.), CPME (0.1 mL), 90°C, 5 h, under Ar. [b] 16 h. [c] 24 h.

With the optimized reaction conditions in hand, the Pd/Si‐catalyzed Heck–Cassar protocol was tested with variously decorated partners to demonstrate its reliability and robustness. First, various terminal alkyne derivatives were tested (Scheme 1a). Next, several electrophile partners were employed to synthesize various diarylalkynes (Scheme 1b). Finally, diversely decorated partners were tested to expand the scope of accessible structures (Scheme 1c). The developed methodology, operating under highly concentrated conditions, enabled effective coupling with a wide array of coupling partners. A series of iodobenzenes and phenylacetylenes bearing diverse substituents, including electron‐withdrawing groups (─Cl, ─Br, ─CF_3_, ─COOEt) and electron‐donating groups (─Me, ─OMe, ─Et, tBu, ─NH_2_), as well as sterically encumbered substrates (2‐Me), underwent efficient coupling to afford the corresponding alkynylated products in uniformly high yields. In contrast, the use of less reactive aryl electrophiles, such as naphthalene derivatives, yielded the product in moderate quantity (70%, **3n**). Some of the synthesized products belong to classes of valuable precursors for biologically active compounds and advanced functional materials. In particular, compound **3b** serves as a precursor for molecules employed in liquid crystal compositions [61] as well as in optoelectronic applications [62]. Compound **3f** is a precursor for glucocerebrosidase modulators [63] and an intermediate for compounds designed to reduce uric acid levels [64]. Notably, when acetyl‐triisopropylsilane was employed as the coupling partner, the desired product was still obtained in excellent yield (**3 g, 3 t**), further highlighting the adaptability of the methodology, given the versatility of these building blocks [65, 66]. Finally, compound **3h** is a valuable precursor for the synthesis of *N*‐ and *O*‐heterocycles, as demonstrated in several recent studies [67, 70].

A large‐scale experiment (5 mmol) for the synthesis of product **3a** was performed without any loss of efficiency, affording a 99% yield.

Pd contamination in the final product was assessed after isolation. For compound **3a,** purified without chromatography, the Pd content was 5.2 ppm, well below the FDA‐permitted daily exposure (PDE) of 10 ppm/day [71, 73]. As a representative counterexample, compound **3b**, which required a longer reaction time of 16 h, retained 12 ppm of Pd, exceeding the regulatory limit and necessitating an additional purification step.

**SCHEME 2:**
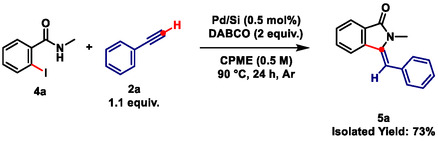
Pd/Si promoted tandem Heck–Cassar and cyclization.

The effectiveness of the developed reaction conditions in combination with our Pd/Si catalyst was tested for the synthesis of 3‐methyleneisoindolin‐1‐ones via a tandem Heck–Cassar coupling followed by intramolecular cyclization [74, 75]. The reaction proceeds through the coupling of 2‐iodobenzamides with terminal alkynes; subsequent addition of two equivalents of DABCO promotes amine deprotonation and triggers cyclization, affording the desired products in an isolated yield of 73% (Scheme 2) (Optimization of the reaction conditions is reported in the Supporting Information).

Given the excellent catalyst stability and efficacy and the complete solubility of the DABCO in CPME, the appropriate conditions and flow setup were optimized to define a continuous‐flow protocol that balances efficiency and long‐term productivity. A blend of Pd/Si:quartz powder (10:90) was used to pack PTFE reactors with an internal diameter of 1/4″, thermoset at 90°C. Concentration, flow rate, temperature, and Back Pressure Regulator (BPR) were systematically varied to evaluate their effect on conversion. We started with a CPME 2 mol/L to ensure complete solubility of the reaction components (Table 2).

**TABLE 2:** Heck–Cassar cross‐coupling reaction conditions optimization under continuous‐flow.

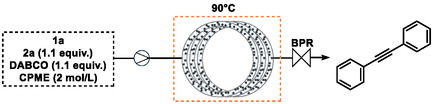						
**Entry** a	**Molarity, M**	**Flow rate,** **mL/min**	**Residence time, min**	**T,** **°C**	**BPR, psi**	**C, %** b
1	2	0.5	9	90	75	8
2	2	0.2	22	90	75	16
3	2	0.1	44	90	100	40
4	2	0.03	148	90	100	43
5	1	0.03	148	90	100	41
6	0.5	0.03	148	90	100	45
7^c^	0.5	0.1	67	90	100	60
8^c^	0.5	0.03	222	90	250	78
9^c^	0.5	0.03	222	110	250	65
10^c^, ^d^	0.5	0.03	222	90	250	45
11^e^	0.5	0.015	739	90	250	92
12^e^	0.5	0.010	1109	90	250	99
13^e^	1	0.010	1109	90	250	99

a Reaction condition: Pd/Si (16%), **1a** (1 mmol), **2a** (1.1 equiv.), DABCO (1.1 equiv.), CPME, 90°C, 1 m of reactor length (333 mg of Pd/Si 2.7 wt%).

b GC conversion has been determined using samples of pure compounds as reference standards.

c 1.5 m of reactor length (500 mg of Pd/Si 2.7 wt%).

d DABCO (1.5 equiv.).

e 2.5 m of reactor length, (833 mg of Pd/Si 2.7 wt%).

Decreasing the flow rate from 0.5 to 0.03 mL/min significantly increased conversion (entries 1–4). Increasing the BPR value strongly improved conversion (entries 3–4). Changes in molarity from 2.0 to 0.5 M had a limited effect under comparable conditions (entries 4–6). A longer reactor (1.5 m) enabled higher conversion (entry 7). Combining this with a 250 psi BPR yielded an increase in conversion (entry 8). Increasing the temperature to 110°C resulted in lower conversion under otherwise identical conditions (entry 9). Increasing the amount of DABCO did not increase conversion (entry 10). Combining a longer reactor (2.5 m) with a 250 psi BPR yielded nearly quantitative conversion at a flow rate of 0.03 mL/min (entry 11). Further decreasing the flow rate to 0.01 mL/min led to full conversion (entry 12–13).

**FIGURE 4:**
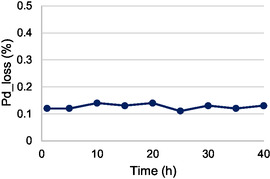
Pd‐loss (%) versus time (h).

A 40 h long‐term experiment was conducted under the optimized reaction conditions (entry 12), the process delivered 11.76 mmol of product **3a** (98% of isolated yield) over 40 h, corresponding to a productivity of 0.3 mmol/h, a space‐time yield (STY) of 3.7 mmol L^−1^ h^−1^, liquid hourly space velocity (LHSV) of 0.054 h^−1^ and observing a stable Pd loss around 0.1% (Figure 4).

**SCHEME 3:**
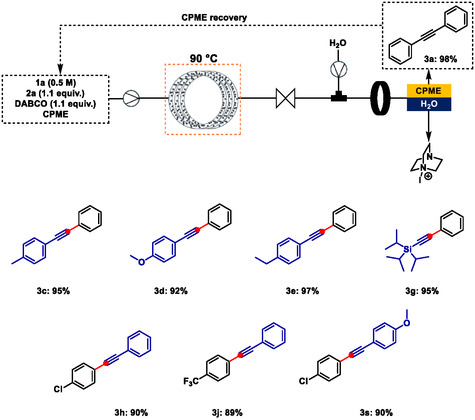
Pd/Si promoted tandem Heck–Cassar and cyclization. Reaction condition: Pd/Si (16%), **1a** (0.5 M), **2a** (1.1 equiv.), DABCO (1.1 equiv.) in CPME, 90°C, 2.5 m of reactor length, (833 mg of Pd/Si 2.7 wt%).

The setup was equipped with a water introduction system to generate a biphasic system and a residence loop to enhance phase mixing. The reactor outlet was equipped with a liquid–liquid membrane separator, enabling continuous separation of the organic and aqueous phases. This configuration allowed for an optimized work‐up procedure. In particular, CPME was recovered in pure form by simple distillation and directly reused as a reaction medium in successive reactions without further purification, while the aqueous DABCO–I^‐^ solution constituted the sole waste stream of the process. With the optimized continuous‐flow set‐up, we explored the scope of the Heck–Cassar reaction, achieving isolated yields ranging from 89% to 98% for representative 8 substrates screened (Scheme 3).

**FIGURE 5:**
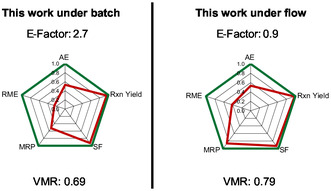
Green metrics comparison under batch and flow conditions. Atom Economy (AE); Rxn Yield (Reaction Yield); Stoichiometric Factor (SF); Material Recovery Parameters (MRP); Reaction Mass Efficiency (RME); Vector Magnitude Ratio (VMR).

As part of ongoing efforts to develop more sustainable synthetic protocols, the process’s environmental footprint was evaluated [76, 79]. Excellent E‐factor values were obtained for both batch and flow conditions, calculated representatively for the synthesis of product **3a** (2.7 and 0.9, respectively), representing a significant improvement compared to literature precedent, which typically reports E‐factors of around 8.6 in batch and 2.8 in flow [53]. This enhancement can be attributed to a substantial reduction in waste generation, primarily due to the lower excesses of alkyne and DABCO required in our protocol, as reflected by the excellent stoichiometric factor. Solvent recovery via distillation during both the reaction and work‐up stages results in a sensible increase in the Material Recovery Parameter (MRP), with a corresponding improvement in the Reaction Mass Efficiency (RME) and Vector Magnitude Ration (VMR) (Figure 5) (see Supporting Information for calculations).

## 3 Conclusion

In summary, we have demonstrated the use of silicon as a support for palladium nanoparticle‐based catalysts (Pd/Si), an approach that can be extended to silicon derived from e‐waste and photovoltaic sources. The resulting catalyst proved effective in a packed‐bed reactor and demonstrated robust, efficient performance in the Heck–Cassar cross‐coupling reaction. Notably, the reaction was successfully performed in CPME, a reaction medium that display a distinctly favorable profile, under both batch and continuous‐flow conditions. Exploiting the catalyst’s high stability and reactivity, we implemented a continuous‐flow protocol that operated for 40 h. The system showed stable efficiency with minimal palladium loss, underscoring its sustainability. Moreover, the successful application of this method in intramolecular cyclization reactions further demonstrates its versatility. Finally, the lower E‐factor of 0.9 obtained under flow conditions further attests to the methodology’s favorable environmental profile. The catalyst showed good recyclability over 5 consecutive runs, with no significant changes in Pd nanoparticles, while operating under a release‐and‐catch mechanism, as previously reported for a Pd‐nanoparticle‐based catalyst in the literature. The developed reaction conditions were applied to the synthesis of 20 variously functionalized alkynes, and the catalyst was used in a tandem Heck–Cassar coupling followed by intramolecular cyclization to yield 3‐methyleneisoindolin‐1‐ones.

## Funding

This study was supported by European Commission (ECS00000041 – VITALITY, Ecosistema TECH4YOU – (Spoke 3 – Goal 3.5)), Ministero dell’Università e della Ricerca (20223ARWAY – REWIND), Ministero degli Affari Esteri e della Cooperazione Internazionale (HYDROFA), Council of Higher Education (IL) (Rita Levi Montalcini Award program).

## Conflicts of Interest

The authors declare no conflicts of interest.

## Supporting information

Supplementary Material

## Data Availability

The data that support the findings of this study are available on request from the corresponding author. The data are not publicly available due to privacy or ethical restrictions.
