# Supplementary material for: Upcycling Silicon as Heterogeneous Palladium Catalysts: Heck–Cassar Cross‐Coupling in Batch and Flow Conditions
Source: ChemSusChem. 2026 Jun 27;19(13):e70827. doi: 10.1002/cssc.70827 (PMC13309916; doi:10.1002/cssc.70827)

SUPPORTING INFORMATION

Upcycling Silicon as Heterogeneous Palladium Catalysts: Heck-Cassar Cross-Coupling in Batch and Flow Conditions.

Tian Sang,[a†] Giulia Brufani,[b†] Tommaso Scarabottini,[a] Sofiya Zabelinskaya,[a,c] Dmitri Gelman,[c] Francesco Mauriello,[b] Luigi Vaccaro[a*]*

[a]Laboratory of Green S.O.C. – Dipartimento di Chimica, Biologia e Biotecnologie, Università degli Studi di Perugia. Via Elce di Sotto 8, 06123, Perugia, Italy.
[b]Department of Civil, Energy, Environmental and Material Engineering (DICEAM), Università degli Studi Mediterranea di Reggio Calabria. Via Graziella, Feo di Vito, Reggio Calabria 89122, Italy.
[c]Institute of Chemistry. The Hebrew University. 91904 Jerusalem, Israel

† These authors contributed equally to this work

E-mail: luigi.vaccaro@unipg.it

Total number of pages: 60

total number of figures: 23 at page S9, S38-S59

total number of tables: 7 at pages S5-S8

TABLE OF CONTENTS:

1. General Remarks S2
2. General Procedures S4
3. Mechanistic Investigation S5
4. E-factor Calculation S7
5. Radial Polygon Metrics Calculation S7
6. Optimization of the Reaction Conditions and Characterization of 5a S8
7. Characterization Data S10
8. ^{1}H, ^{13}C and ^{19}F NMR Spectra S38

1. General Remarks

The reagents used in this work were purchased from Merck and Zentek and used without further purification or activation procedures. Silicon powder (325 mesh, 7440-21-3) was purchased from Merck.

Gas chromatography (GC) analyses were conducted using Agilent 6850 and Shimadzu GC 2010 Pro, which were equipped with a 5-MS column and helium as the carrier gas. The temperature ramp involves an initial time of 1 minute at 70°C, then increasing by 15°C every minute up to a final temperature of 300°C for 5 minutes.

Gas chromatography-mass spectroscopy (GC-MS) analysis was conducted by Agilent 8890 GC system 5977 GC/MSD instruments and Hewlett-Packard HP 6890N Network GC system/5975 Mass Selective Detector equipped with an electron impact ioniser at 70 eV.

Melting points were measured on a Buchi 510 apparatus.

XPS measurements were performed in a home-built ultra-high-vacuum (UHV) chamber with a base pressure of 10^{-9} mbar. Samples were prepared by pressing the powder onto carbon tape. A non-monochromatic Al Kα X-ray source (hv = 1486.6 eV, VSW-A10) operated at 120 W (12 kV, 10 mA) was used in combination with a hemispherical electron/ion energy analyzer (VSW-HA100) equipped with a 16-channel detector. Photoelectrons were collected in the normal emission direction, with a 54.5° angle between the analyzer axis and the X-ray source. Spectra were acquired in Fixed Analyzer Transmission (FAT) mode with a pass energy of 44 eV. Energy calibration was performed by setting the aliphatic C 1s component to 285.1 eV. Data analysis was carried out using CasaXPS software. Background subtraction was performed using a Shirley function. Peak deconvolution was carried out using a Gaussian/Lorentzian (GL) line shape for C 1s, K 2p, and Co 2p regions, and an asymmetric Lorentzian (LA) line shape for Si 2p and Pd 3d regions. Empirical sensitivity factors were used to calculate semi-quantitative atomic ratios.

High-resolution transmission electron microscopy (HR-TEM): Thermo Scientific™ Talos™ F200X scanning/transmission electron microscope (S/TEM) with energy dispersive x-ray spectroscopy (EDS) signal detection.

^{1}H-NMR, ^{13}C-NMR and ^{19}F-NMR analyses were obtained using a Bruker DRX-ADVANCE 400 MHz NMR spectrometer, with $CDCl_3$ and $(CD_3)_2CO$ as the solvent and reference standard. All the NMR Spectra reported present around 95% purity.

Solvent Distillation was performed with BUCHI Kugelrohr B-585.

Centrifugation was carried out using a Beckman Coulter Allegra 64 R centrifuge for the catalyst recovery.

The Agilent Technologies 4210 instrument was used to determine the metal content via microwave plasma atomic emission spectroscopy (MP-AES).

The reaction mixture was introduced into the flow setup using a high-performance liquid chromatography (HPLC) pump LC-40D.

A Zaiput ETFE liquid-liquid/gas-liquid separator was used for in-line liquid-liquid phase separation.

2. General procedures

2.1. Pd/Si synthesis.

In a 50 mL two-neck round-bottom flask equipped with a condenser, 400 mg of commercial silicon was suspended in 20 mL of diethylene glycol and subjected to ultrasonic treatment. An aqueous solution of $PdCl_2$ (prepared by dissolving 75 mg of $PdCl_2$ in 1 mL of HCl, 33% w/w, at 80 °C) was then added dropwise under sonication. The pH of the suspension was adjusted to 10 by the addition of NaOH solution (5 M). The mixture was refluxed at 130 °C under an argon atmosphere for 3 h. After cooling to room temperature, the suspension was filtered through a sintered-glass funnel to recover the Pd/Si catalyst. The solid was washed thoroughly with hot water, ethanol, and acetone, and subsequently dried under vacuum. The loading of Pd (2.7 wt%) was measured by MPAES analysis.

2.2. Typical procedure for the Cassar-Heck coupling under batch.

In a 2 mL screw-capped vial equipped with a magnetic stir bar, Pd/Si catalyst (2.7 wt%, 0.5 mol%), DABCO (1.1 equiv.), iodobenzene (**1a**) (1 mmol), phenylacetylene (**2a**) (1.1 equiv.) and CPME (10 M) were sequentially added. The mixture was purged with Ar and stirred at 90 °C in a heating module. After 5 h, the reaction mixture was cooled to room temperature, and the catalyst was recovered by centrifugation (6000 rpm for 10 min) and washed 3 times with fresh CPME (2 mL each time). The organic phase was collected and extracted three times with an equal volume of water. The CPME used was distilled and recovered (98%) to afford diphenylacetylene (**3a**) as a pale-yellow solid. The water used for the extraction was recovered via distillation (96%)

2.3. Typical procedure for the Cassar-Heck coupling under flow.

The continuous flow Sonogashira coupling was performed using two Shimadzu LC-20AD HPLC pump and a PTFE column (2.5 m, ID: ¼") fitted with HPLC peek connections. The reactor column was packed with 833 mg of Pd/Si (2.7 % wt) dispersed in 5.55 g of solid-glass beads (1.0 mm particle size). Iodobenzene (1 mmol) and phenylacetylene (1.1 mmol), and DABCO (1.1 mmol) were completely dissolved in CPME (0.5 M for iodobenzene). The line was directly inserted into the reactor column, thermostated at 90 °C, and equipped with a 250 psi BPR. The flow rate was 0.01 mL min^{-1}. Then a second channel was connected to pump water into the line. The two lines were connected via a T-junction, and then a loop (2 m, 1/16”) was added to help the mixing. The organic phase was separated using a Zaiput liquid/liquid separator. The water phase containing DABCO-I is directed to the waste disposal after water distillation. The organic phase (CPME) containing the products and the unreacted acetylenic compounds was directed to a continuous distillation apparatus. The CPME was recovered at 106-108 °C and, after NMR analysis to confirm the purity, was reused continuously. The crude reaction mixture for each compound

was evaporated to remove the unreacted acetylenic compound, yielding the pure products.

2.4. Pd/Si recycle.

After the reaction was completed, the catalyst was recovered by centrifugation, washed with CPME (3 × 2 mL) and water (2 mL), and dried under vacuum at 80 °C for 16 h. The dried catalyst was reused under the optimized conditions without significant mass loss.

2.5. General procedure for leaching determination

The crude reaction mixture, after separation from the catalyst, was dried under vacuum, dissolved in 2 mL of aqua regia, and stirred for 1 h at room temperature. The mixture was transferred into a 10 mL graduated flask and Milli-Q water was added to reach the final volume. If present, residual solids were filtered off, and the sample was analyzed by the MP-AES 4210 instrument.

2.6. General procedure for Hg-poisoning test.

In a 4 mL screw-capped vial equipped with a magnetic stirrer, Pd/Si catalyst (2.7 wt%, 0.5 mol%), DABCO (1.1 equiv.), iodobenzene (1 mmol), Phenylacetylene (1.1 equiv.), and CPME (10 M) were consecutively added, and the mixture was kept under stirring at 90 °C. After 1 h, 4 μL Hg (100 equiv. respect to the Pd) were added, and the mixture was kept under stirring for an additional 5 h. The product distribution over time was determined by GC analysis. The results are reported in **Table S1**.

3. Mechanistic Investigation

Table S1. Mercury drop test.

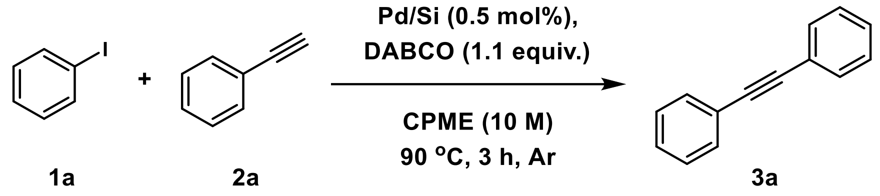

Entry [a]	catalytic test	t (h)	C(%) [b]
1	Before Hg-poisoning test	1	84
2[c]	After Hg-poisoning test	5	85

[a] Reaction conditions: Pd/Si (0.5 mol%), **1a** (1 mmol), **2a** (1.1 equiv.), DABCO (1.1 equiv.), solvent (0.1 mL), 90 °C, under Ar. [b] Conversion has been determined using samples of pure compounds as reference standards. [c] After one hour of reaction, add Hg reagent.

Table S2. Kinetic study of Pd/Si catalyst under optimized reaction conditions.

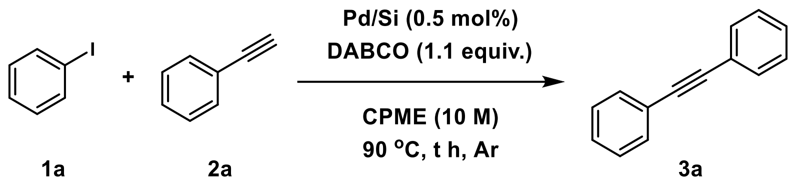

1a 2a 3a

Entry [a]	t (h)	C (%) [b]
1	0.5	80
2	1	84
3	2	89
4	3	90
5	4	93
6	5	99

[a] Reaction conditions: Pd/Si (0.5 mol%), **1a** (1 mmol), **2a** (1.1 equiv.), DABCO (1.1 equiv.), solvent (0.1 mL), 90 ℃, t h, under Ar. [b] Conversion has been determined using samples of pure compounds as reference standards.

4. E-Factor Calculation

$$EFactor = \frac{Mass\ of\ row\ waste - Mass\ of\ recovered\ materials}{Mass\ of\ product}$$

Table S3. E-Factor Calculation under batch conditions (5 mmol)

Iodobenzene (**1a**)	1.02
Phenylacetylene (**2a**)	0.56
DABCO	0.62
CPME (solvent reaction)	0.43
Pd/Si	0.10
CPME (workup)	5.16
H_2O (workup)	6.00
Pd/Si recovered	0.10
CPME recovered	5.42
H_2O water recovered	5.70
Diphenylacetylene (**3a**)	0.78

Table S4. E-Factor calculation under flow conditions (12 mmol)

Iodobenzene (**1a**)	2.45
Phenylacetylene (**2a**)	1.35
DABCO	1.48
CPME (solvent reaction)	20.64
H_2O (workup)	20.00
CPME recovered	23.2
H_2O water recovered	19.00
Diphenylacetylene (**3a**)	1.92

5. Radial Polygon Metrics Calculation

Calcation were performed with the methods and the equations reported by J. Andraos in J. Chem. Educ. 92 (2015) 1820−1830

$$AE = \frac{MW_{reactant}}{MW_{product}} \qquad SF = \frac{Actual\ Mass\ of\ Reagent}{Stoichiometric\ Mass\ of\ Reagents}$$

$$MRP = \frac{RME \cdot SF}{AE \cdot Yield} \qquad RME = \frac{1}{(1 + EFactor)}$$

$$VMR = \frac{\sqrt{AE^2 + Yield^2 + \left(\frac{1}{SF}\right)^2 + MRP^2 + RME^2}}{\sqrt{5}}$$

Table S5. Calculation under batch conditions (5 mmol)

Parameter	Actual
AE	0.53
Rxn Yield	0.99
SF	0.95
MRP	0.55
RME	0.27
VMR	0.71

Table S6. Calculation under flow conditions (12 mmol)

Parameter	Actual
AE	0.53
Rxn Yield	0.99
SF	0.95
MRP	0.94
RME	0.47
VMR	0.81

6. Optimization of the Reaction Conditions and characterization of 5a

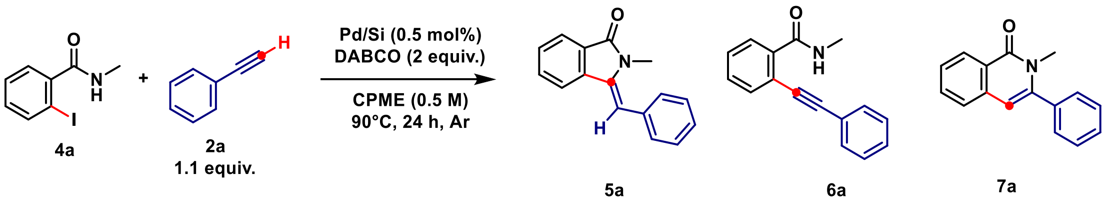

Table S7. Reaction conditions optimization for the synthesis of **5a**

Entry	4a:2a	Pd/Si (mol%)	DABCO (equiv.)	C (%)	Selectivity (%) 5a:6a:7a
1	1:1	1	1	89	12:83:5
2	1.1:1	0.5	1	90	10:84:6
3[d]	1:1.1	0.5	2	90	82:18:0
4	1:1.2	0.5	1.2	89	10:84:6

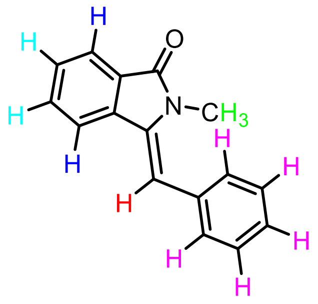

Figure S1. 1H-COSY of **5a**

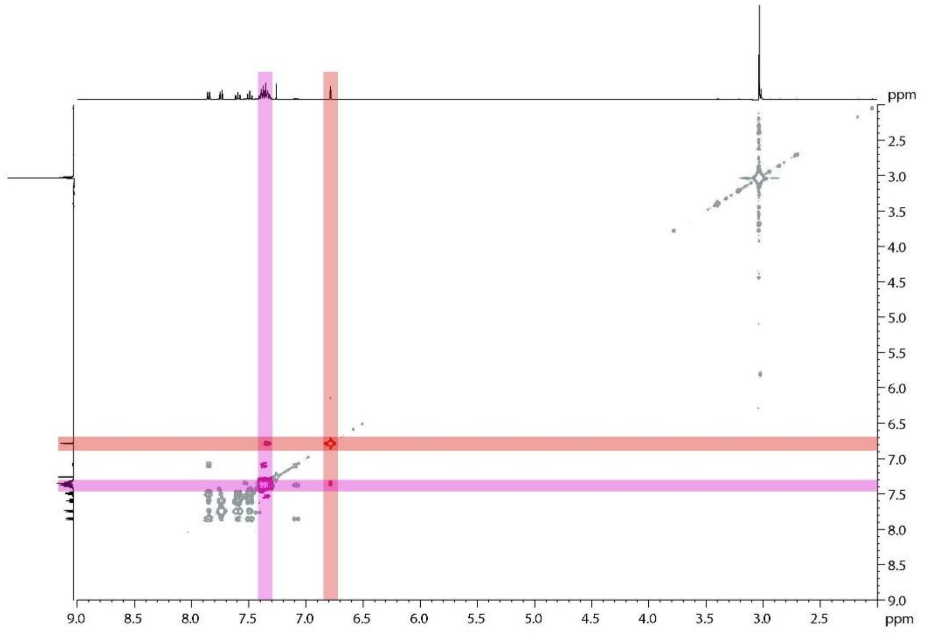

Figure S2. 1H-NOESY **of 5a**

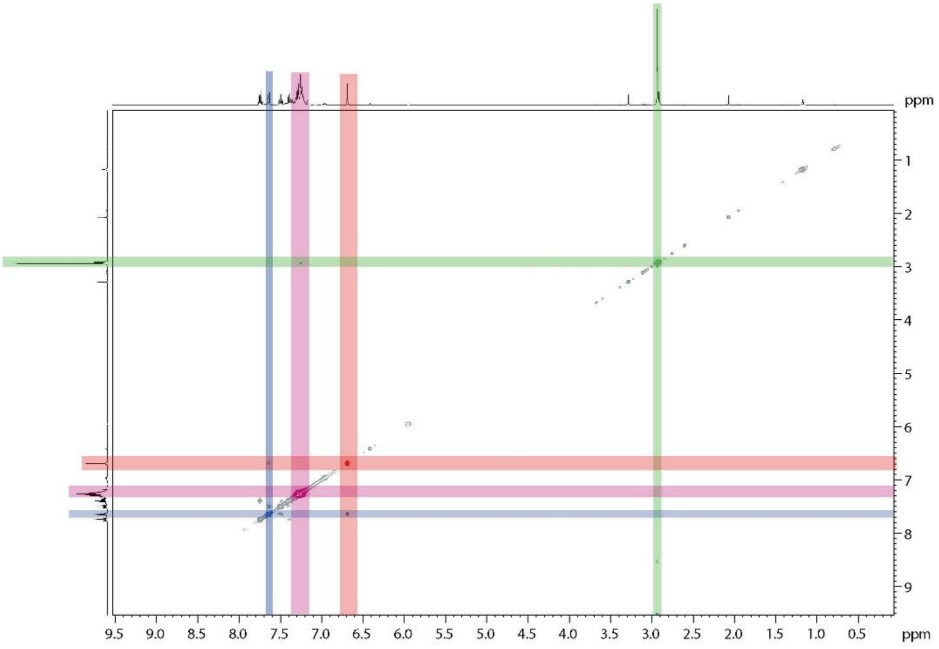

7. Characterization Data

**Chem. Name**	**1,2-diphenylethyne (3a)**			
**Lit. Ref.**	Green Chem., 2012, 14, 2840–2855			

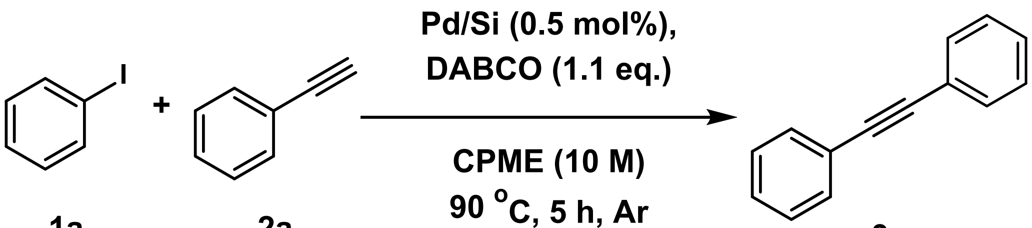

1a **2a** **3a**

M. W.: 178.23 g/mol

METHOD:

In a 2 mL screw-capped vial equipped with a magnetic stir bar, Pd/Si catalyst (5.7 wt%, 0.5 mol%, 9.3 mg), DABCO (1.1 equiv., 1.1 mmol, 123.4 mg), iodobenzene **1a** (1 mmol, 204.0 mg, 67.7 μL), Phenylacetylene **2a** (1.1 equiv., 1.1 mmol, 112.3 mg, 120.7 μL) and CPME (0.1 mL) were sequentially added. The mixture was purged with Ar and stirred at 90°C in a heating module. After 5 h, the reaction mixture was cooled to room temperature and the catalyst was recovered by centrifugation (6000 rpm, 10 min), washing three times with fresh CPME. The organic phase was collected and extracted three times with an equal volume of water. The combined organic layers were concentrated under reduced pressure, and the residue was further purified by rotary evaporation under vacuum, affording diphenylacetylene **(3a)** as a pale-yellow solid in 99% isolated yield (175.7 mg).

**Mol Formula**	$C_{14}H_{10}$	**m.p.**	64-65 °C	
**^{1}H NMR 400 MHz $CDCl_3$**	**δ value**	**No. H**	**Mult.**	**j value/Hz**
	7.60-7.53	4	m	-
	7.41-7.34	6	m	-

^{13}C NMR (100.6 MHz, $CDCl_3$) δ: 131.66, 128.39, 128.29, 123.33, 89.81.

GC-EIMS (m/z, %):179 (15), 178 (100), 176 (21)

Chem. Name	**1-bromo-4-(phenylethynyl)benzene (3b)**
**Lit. Ref.**	Chem. Commun., 2025,61, 9960-9963

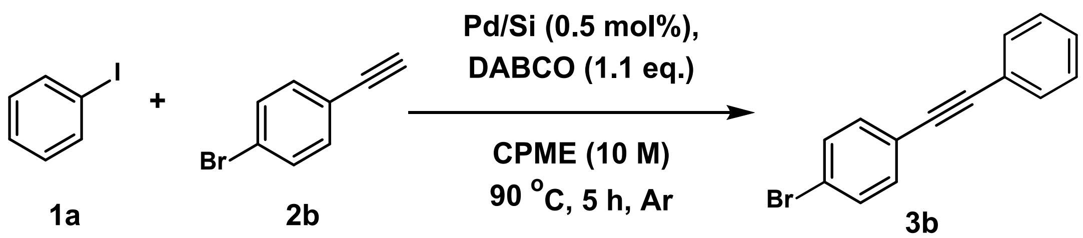

M. W.: 257.13 g/mol

METHOD:

In a 2 mL screw-capped vial equipped with a magnetic stir bar, Pd/Si catalyst (5.7 wt%, 0.5 mol%, 9.3 mg), DABCO (1.1 equiv., 1.1 mmol, 123.4 mg), iodobenzene **1a** (1 mmol, 204.0 mg, 67.7 µL), 1-bromo-4-ethynylbenzene **2b** (1.1 equiv., 1.1 mmol, 199.1 mg) and CPME (0.1 mL) were sequentially added. The mixture was purged with Ar and stirred at 90 °C in a heating module. After 5 h, the reaction mixture was cooled to room temperature and the catalyst was recovered by centrifugation (6000 rpm, 10 min), washing three times with fresh CPME. The organic phase was collected and extracted three times with an equal volume of water. The combined organic layers were concentrated under reduced pressure, and the residue was further purified by rotary evaporation under vacuum, affording 1-bromo-4-(phenylethynyl)benzene **(3b)** as a yellow solid in 94% isolated yield (241.3 mg).

**Mol Formula**	$C_{14}H_9Br$	**m.p.**	80-83 °C	
**^{1}H NMR 400 MHz $CDCl_3$**	**δ value**	**No. H**	**Mult.**	**j value/Hz**
	7.56 – 7.51	2	m	-
	7.49	2	d	8.6
	7.39	2	d	8.6
	7.37 – 7.31	3	m	-

^{13}C NMR (100.6 MHz, $CDCl_3$) δ: 133.02, 131.62, 131.60, 128.51, 128.40, 122.92, 122.48, 122.26, 90.51, 88.31.

GC-EIMS (m/z, %): 259 (64), 258 (100), 218 (25), 217 (100), 216 (100), 215 (100), 199 (25), 189 (50), 188 (100), 187 (100), 175 (77), 174 (100), 173 (100), 171 (89), 161 (100), 160 (100), 159 (100), 157 (100), 155 (35), 147 (100), 146 (100), 145(100), 143 (97), 142 (23), 137 (25), 136 (27), 135 (100), 133 (38), 132(54), 131(100), 130 (100), 129(100), 121 (100), 119 (40), 117(26), 115(59), 113 (26), 107 (37), 106 (45), 105 (100), 104 (38), 103 (100=, 102 (30), 95 (25), 93 (27), 91 (22), 85 (48), 83 (41), 80 (94), 77 (48), 73 (71), 71 (28), 69 (25), 67 (27), 59 (100), 55 (29), 45 (36), 44 (23), 43 (100), 42 (49), 41 (100), 39 (42).

Chem. Name	1-methyl-4-(phenylethynyl)benzene (3c)
Lit. Ref.	Green Chem., 2012, 14, 2840–2855

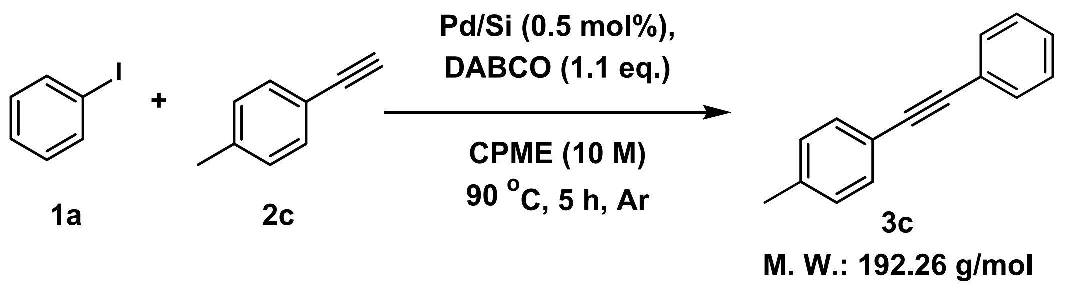

METHOD:

In a 2 mL screw-capped vial equipped with a magnetic stir bar, Pd/Si catalyst (5.7 wt%, 0.5 mol%, 9.3 mg), DABCO (1.1 equiv., 1.1 mmol, 123.4 mg), iodobenzene **1a** (1 mmol, 204.0 mg, 67.7 µL), 1-ethynyl-4-methylbenzene **2c** (1.1 equiv., 1.1 mmol, 127.8 mg, 139.4 µL) and CPME (0.1 mL) were sequentially added. The mixture was purged with Ar and stirred at 90 °C in a heating module. After 5 h, the reaction mixture was cooled to room temperature and the catalyst was recovered by centrifugation (6000 rpm, 10 min), washing three times with fresh CPME. The organic phase was collected and extracted three times with an equal volume of water. The combined organic layers were concentrated under reduced pressure, and the residue was further purified by rotary evaporation under vacuum, affording 1-methyl-4-(phenylethynyl)benzene **(3c)** as a white solid in 94% isolated yield (180.2 mg).

**Mol Formula**	$C_{15}H_{12}$	**m.p.**	70-72 °C	
**^{1}H NMR 400 MHz $CDCl_3$**	**δ value**	**No. H**	**Mult.**	**j value/Hz**
	7.53	2	dd	7.6, 2.2
	7.44	2	d	8.1
	7.38 – 7.31	3	m	-
	7.16	2	d	8.1
	2.38	3	s	-

^{13}C NMR (100.6 MHz, $CDCl_3$) δ: 138.38, 131.55, 131.50, 131.25, 129.12, 129.01, 128.31, 128.27, 128.07, 127.03, 21.54.

GC-EIMS (m/z, %):193 (16), 192 (100), 191 (52), 189 (21)

<table>
<tr><td>Chem. Name</td><td colspan="4">1-methoxy-4-(phenylethynyl)benzene (3d)</td></tr>
<tr><td>Lit. Ref.</td><td colspan="4">Chem. Commun., 2025,61, 9960-9963</td></tr>
<tr><td colspan="5">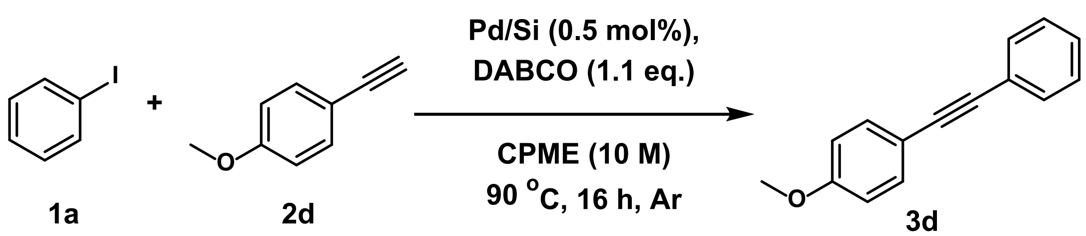

1a 2d 3d
M. W.: 208.26 g/mol</td></tr>
<tr><td colspan="5">METHOD:
In a 2 mL screw-capped vial equipped with a magnetic stir bar, Pd/Si catalyst (5.7 wt%, 0.5 mol%, 9.3 mg), DABCO (1.1 equiv., 1.1 mmol, 123.4 mg), iodobenzene 1a (1 mmol, 204.0 mg, 67.7 µL), 1-ethynyl-4-methoxybenzene 2d (1.1 equiv., 1.1 mmol, 127.8 mg, 139.4 µL) and CPME (0.1 mL) were sequentially added. The mixture was purged with Ar and stirred at 90 °C in a heating module. After 16 h, the reaction mixture was cooled to room temperature and the catalyst was recovered by centrifugation (6000 rpm, 10 min), washing three times with fresh CPME. The organic phase was collected and extracted three times with an equal volume of water. The combined organic layers were concentrated under reduced pressure, and the residue was further purified by rotary evaporation under vacuum, affording 1-methoxy-4-(phenylethynyl)benzene (3d) as a white solid in 96% isolated yield (199.6 mg).</td></tr>
<tr><td>Mol Formula</td><td>$C_{15}H_{12}O$</td><td>m.p.</td><td colspan="2">90-93°C</td></tr>
<tr><td rowspan="5">^{1}H NMR
400 MHz
$CDCl_3$</td><td>δ value</td><td>No. H</td><td>Mult.</td><td>j value/Hz</td></tr>
<tr><td>7.58 – 7.45</td><td>4</td><td>m</td><td>-</td></tr>
<tr><td>7.34</td><td>3</td><td>m</td><td>-</td></tr>
<tr><td>6.89</td><td>2</td><td>d</td><td>8.8</td></tr>
<tr><td>3.83</td><td>3</td><td>s</td><td>-</td></tr>
<tr><td colspan="5">^{13}C NMR (100.6 MHz, $CDCl_3$) δ: 133.60, 133.07, 131.46, 128.32, 127.95, 115.38, 114.01, 113.97, 113.95, 88.08, 55.31.</td></tr>
<tr><td colspan="5">GC-EIMS (m/z, %): 208 (100), 193 (10), 165 (50), 164 (18), 163 (13), 139 (13)</td></tr>
</table>

Chem. Name	1-ethyl-4-(phenylethynyl)benzene (3e)
Lit. Ref.	Appl Organomet Chem., 2024, 38, e7405.

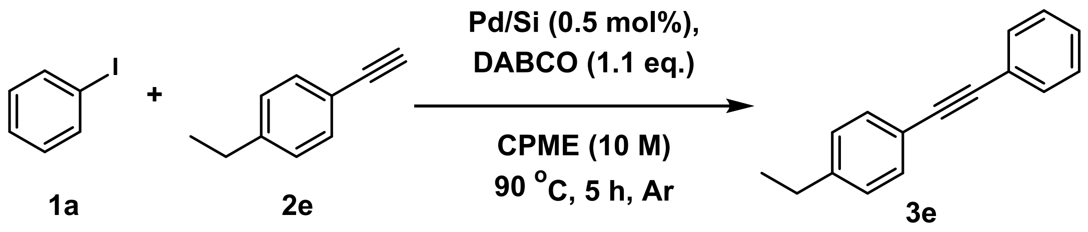

M. W.: 206.29 g/mol

METHOD:
In a 2 mL screw-capped vial equipped with a magnetic stir bar, Pd/Si catalyst (5.7 wt%, 0.5 mol%, 9.3 mg), DABCO (1.1 equiv., 1.1 mmol, 123.4 mg), iodobenzene **1a** (1 mmol, 204.0 mg, 67.7 µL), 1-ethyl-4-ethynylbenzene **2e** (1.1 equiv., 1.1 mmol, 143.2 mg, 154.0 µL) and CPME (0.1 mL) were sequentially added. The mixture was purged with Ar and stirred at 90 °C in a heating module. After 5 h, the reaction mixture was cooled to room temperature and the catalyst was recovered by centrifugation (6000 rpm, 10 min), washing three times with fresh CPME. The organic phase was collected and extracted three times with an equal volume of water. The combined organic layers were concentrated under reduced pressure, and the residue was further purified by rotary evaporation under vacuum, affording 1-methoxy-4-(phenylethynyl)benzene **(3e)** as a liquid in 95% isolated yield (195.6 mg).

**Mol Formula**	$C_{16}H_{14}$	**m.p.**	-	
**^{1}H NMR 400 MHz $CDCl_3$**	**δ value**	**No. H**	**Mult.**	**j value/Hz**
	7.56	2	m	-
	7.49	2	d	6
	7.36	3	m	-
	7.21	2	d	7
	2.69	2	q	7.5
	1.28	3	t	7.6

^{13}C NMR (100.6 MHz, $CDCl_3$) δ: 144.71, 131.64, 131.60, 128.36, 128.11, 127.97, 89.67, 88.79, 28.90, 28.88, 15.43, 15.41.

GC-EIMS (m/z, %): 208 (53), 207 (100), 206 (100), 204 (83), 203 (100), 202 (100), 201 (44), 200 (35), 193 (55), 192 (100), 191 (100), 190 (100), 189 (100), 187 (60), 179 (36), 178 (100), 177 (54), 176 (100), 175 (33), 174 (20), 166 (57), 165 (100), 164 (100), 163 (100), 162 (25), 153 (20), 152 (100), 151 (97), 150 (100), 139 (100), 138 (25), 137 (27), 128 (65), 127 (58), 126 (88), 115 (83), 113 (40), 111 (21), 103 (44) 102 (49), 101 (51), 100 (25), 99 (27), 98 (34), 96 (100), 95 (85), 91 (23), 89 (100), 88 (50), 87 (53), 86 (31), 83 (77), 78 (29), 77 (73), 76 (70), 75 (50), 74 (42), 65 (29), 63 (78), 62 (23), 52 (21), 51 (57), 50 (32), 39 (65).

Chem. Name	4-(phenylethynyl)aniline (3f)
Lit. Ref.	Chem. Eur. J., 2025, 31, e202404778.

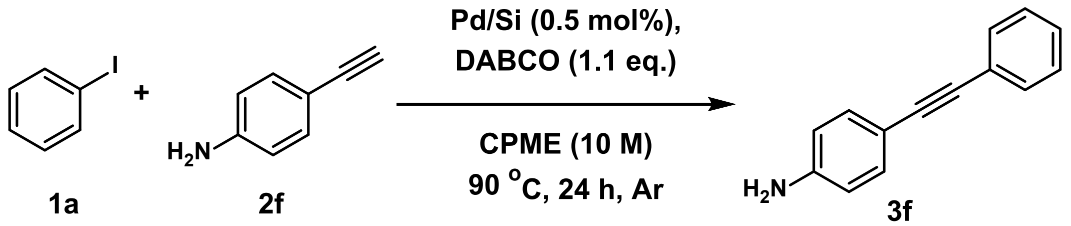

1a 2f 3f

M. W.: 193.25 g/mol

METHOD:

In a 2 mL screw-capped vial equipped with a magnetic stir bar, Pd/Si catalyst (5.7 wt%, 0.5 mol%, 9.3 mg), DABCO (1.1 equiv., 1.1 mmol, 123.4 mg), iodobenzene **1a** (1 mmol, 204.0 mg, 67.7 µL), 4-ethynylaniline **2f** (1.1 equiv., 1.1 mmol, 143.2 mg, 154.0 µL) and CPME (0.1 mL) were sequentially added. The mixture was purged with Ar and stirred at 90 °C in a heating module. After 24 h, the reaction mixture was cooled to room temperature and the catalyst was recovered by centrifugation (6000 rpm, 10 min), washing three times with fresh CPME. The organic phase was collected and extracted three times with an equal volume of water. The combined organic layers were concentrated under reduced pressure, and the residue was further purified by rotary evaporation under vacuum, affording 4-(phenylethynyl)aniline **(3f)** as a brown solid in 63% isolated yield (121.5 mg).

Mol Formula	$C_{14}H_{11}N$	m.p.	124-126 °C	
^{1}H NMR 400 MHz $CDCl_3$	**δ value**	**No. H**	**Mult.**	**j value/Hz**
	7.51	2	m	-
	7.37-7.30	5	m	-
	6.64	2	d	8.6
	3.81	2	s	-

^{13}C NMR (100.6 MHz, $CDCl_3$) δ: 146.01, 132.33, 130.72, 127.64, 127.03, 123.26, 114.12, 112.00, 89.52, 86.72.

GC-EIMS (m/z, %): 195 (28), 194 (100), 193 (100), 191 (100), 190 (62), 177 (25), 176 (28), 167 (23), 166 (70), 165 (100), 164 (89), 163 (62), 152 (42), 151 (24), 150 (23), 141 (25), 140 (32), 139 (78), 126 (43), 115 (48), 113 (25), 97 (100), 96 (28), 90 (21), 89 (47), 88 (20), 87 (24), 84 (48), 83 (35), 77 (21), 71 (29), 63 (31).

Chem. Name	**Triisopropyl(phenylethynyl)silane (3g)**
**Lit. Ref.**	J. Org. Chem., 2023, 88, 11675–11682

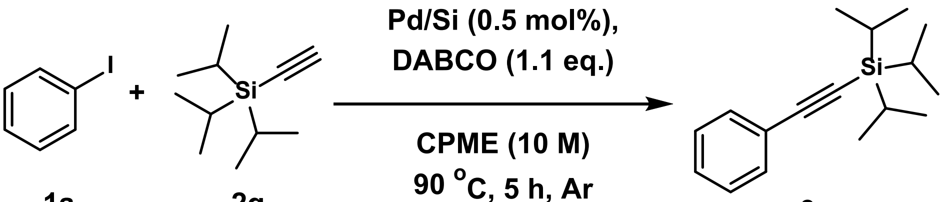

1a **2g** **3g**

M. W.: 258.48 g/mol

METHOD:
In a 2 mL screw-capped vial equipped with a magnetic stir bar, Pd/Si catalyst (5.7 wt%, 0.5 mol%, 9.3 mg), DABCO (1.1 equiv., 1.1 mmol, 123.4 mg), iodobenzene **1a** (1 mmol, 204.0 mg, 67.7 µL), ethynyltriisopropylsilane **2g** (1.1 equiv., 1.1 mmol, 200.62 mg, 246.8 µL) and CPME (0.1 mL) were sequentially added. The mixture was purged with Ar and stirred at 90 °C in a heating module. After 5 h, the reaction mixture was cooled to room temperature and the catalyst was recovered by centrifugation (6000 rpm, 10 min), washing three times with fresh CPME. The organic phase was collected and extracted three times with an equal volume of water. The combined organic layers were concentrated under reduced pressure, and the residue was further purified by rotary evaporation under vacuum, affording Triisopropyl(phenylethynyl)silane **(3g)** as a liquid in 96% isolated yield (247.8 mg).

**Mol Formula**	$C_{17}H_{26}Si$	**m.p.**	-	
**^{1}H NMR 400 MHz $CDCl_3$**	**δ value**	**No. H**	**Mult.**	**j value/Hz**
	7.57 – 7.42	2	m	-
	7.37 – 7.27	3	m	-
	1.16	21	s	-

^{13}C NMR (100.6 MHz, $CDCl_3$) δ: 132.05, 128.29, 128.18, 123.60, 107.15, 90.42, 18.70, 11.37.

GC-EIMS (m/z, %): 258 (17), 215 (100), 173 (37), 131 (22), 105 (7), 43 (42)

Chem. Name	1-chloro-4-(phenylethynyl)benzene (3h)
Lit. Ref.	J. Solid State Chem., 2024, 336, 124736.

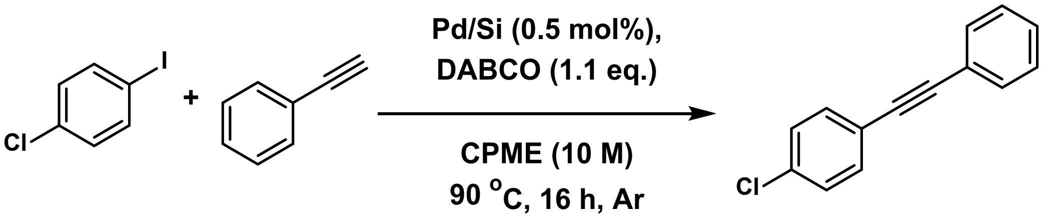

1b **2a** **3h**

M. W.: 212.68 g/mol

METHOD:

In a 2 mL screw-capped vial equipped with a magnetic stir bar, Pd/Si catalyst (5.7 wt%, 0.5 mol%, 9.3 mg), DABCO (1.1 equiv., 1.1 mmol, 123.4 mg), 1-chloro-4-iodobenzene **1b** (1 mmol, 238.5 mg), phenylacetylene **2a** (1.1 equiv., 1.1 mmol, 112.3 mg, 120.7 µL) and CPME (0.1 mL) were sequentially added. The mixture was purged with Ar and stirred at 90 °C in a heating module. After 16 h, the reaction mixture was cooled to room temperature and the catalyst was recovered by centrifugation (6000 rpm, 10 min), washing three times with fresh CPME. The organic phase was collected and extracted three times with an equal volume of water. The combined organic layers were concentrated under reduced pressure, and the residue was further purified by rotary evaporation under vacuum, affording 1-chloro-4-(phenylethynyl)benzene **(3h)** as a yellow solid in 98% isolated yield (208.4 mg).

Mol Formula	$C_{14}H_9Cl$	m.p.	110-111 °C	
^{1}H NMR 400 MHz $CDCl_3$	δ value	No. H	Mult.	j value/Hz
	7.59-7.54	2	m	-
	7.48	2	d	8.5
	7.40 – 7.32	5	m	-

^{13}C NMR (100.6 MHz, $CDCl_3$) δ: 134.30, 132.85, 131.65, 128.74, 128.53, 128.45, 122.98, 121.82, 90.40, 88.33.

GC-EIMS (m/z, %): 212 (100), 214 (33), 177 (25), 176 (17), 151 (47), 106 (12), 75 (4)

Chem. Name	**1-chloro-3-(phenylethynyl)benzene (3i)**			
**Lit. Ref.**	ACS Omega., 2023, 8, 16395–16410.			

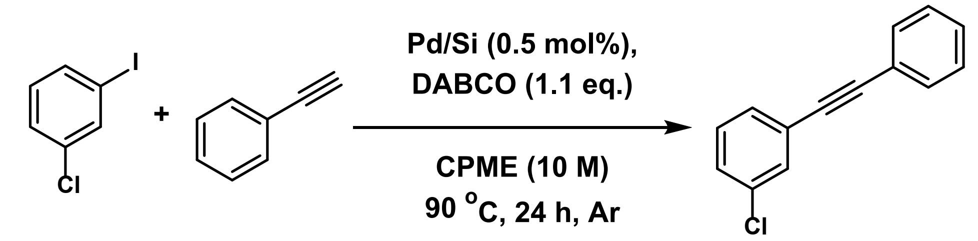

1c **2a** **3i**

M. W.: 212.68 g/mol

METHOD:

In a 2 mL screw-capped vial equipped with a magnetic stir bar, Pd/Si catalyst (5.7 wt%, 0.5 mol%, 9.3 mg), DABCO (1.1 equiv., 1.1 mmol, 123.4 mg), 1-chloro-3-iodobenzene **1c** (1 mmol, 238.5 mg, 123.8 µL), phenylacetylene **2a** (1.1 equiv., 1.1 mmol, 112.3 mg, 120.7 µL) and CPME (0.1 mL) were sequentially added. The mixture was purged with Ar and stirred at 90 °C in a heating module. After 24 h, the reaction mixture was cooled to room temperature and the catalyst was recovered by centrifugation (6000 rpm, 10 min), washing three times with fresh CPME. The organic phase was collected and extracted three times with an equal volume of water. The combined organic layers were concentrated under reduced pressure, and the residue was further purified by rotary evaporation under vacuum, affording 1-chloro-3-(phenylethynyl)benzene **(3i)** as a liquid in 95% isolated yield (201.5 mg).

**Mol Formula**	$C_{14}H_9Cl$	**m.p.**	-	
**^{1}H NMR 400 MHz $CDCl_3$**	**δ value**	**No. H**	**Mult.**	**j value/Hz**
	7.55	3	m	-
	7.42	1	d	7.3
	7.39 – 7.35	3	m	-
	7.32	1	m	-
	7.29	1	d	7.5

^{13}C NMR (100.6 MHz, $CDCl_3$) δ: 134.19, 131.70, 131.46, 129.73, 129.58, 128.68, 128.64, 128.54, 128.51, 128.43, 90.59, 87.96.

GC-EIMS (m/z, %): 212 (100), 214 (35), 177 (27), 176 (16), 151 (48), 106 (11), 75 (3)

Chem. Name	**1-(phenylethynyl)-4-(trifluoromethyl)benzene (3j)**
**Lit. Ref.**	J. Solid State Chem., 2024, 336, 124736.

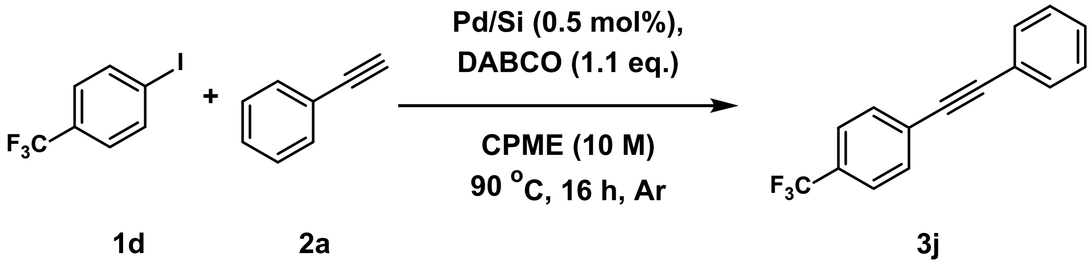

M. W.: 246.23 g/mol

METHOD:

In a 2 mL screw-capped vial equipped with a magnetic stir bar, Pd/Si catalyst (5.7 wt%, 0.5 mol%, 9.3 mg), DABCO (1.1 equiv., 1.1 mmol, 123.4 mg), 1-iodo-4-(trifluoromethyl)benzene **1d** (1 mmol, 272.0 mg, 147.0 µL), phenylacetylene **2a** (1.1 equiv., 1.1 mmol, 112.3 mg, 120.7 µL) and CPME (0.1 mL) were sequentially added. The mixture was purged with Ar and stirred at 90 °C in a heating module. After 16 h, the reaction mixture was cooled to room temperature and the catalyst was recovered by centrifugation (6000 rpm, 10 min), washing three times with fresh CPME. The organic phase was collected and extracted three times with an equal volume of water. The combined organic layers were concentrated under reduced pressure, and the residue was further purified by rotary evaporation under vacuum, affording 1-(phenylethynyl)-4-(trifluoromethyl)benzene **(3j)** as a yellow solid in 86% isolated yield (211.4 mg).

**Mol Formula**	$C_{15}H_9F_3$	**m.p.**	100-102°C	
**^{1}H NMR 400 MHz CDCl$_3$**	**δ value**	**No. H**	**Mult.**	**j value/Hz**
	7.67 – 7.59	4	m	-
	7.58-7.53	2	m	-
	7.41 – 7.35	3	m	-

^{13}C NMR (100.6 MHz, CDCl$_3$) δ: 131.81, 131.75, 130.09, 129.75, 128.84, 128.46, 127.15, 125.35, 125.31, 125.27, 125.24, 91.76, 87.97.

^{19}F NMR (376 MHz, CDCl3) δ: [-62.785].

GC-EIMS (m/z, %): 248 (19), 247(100), 246(100), 245 (85), 228 (22), 227 (100), 226 (19), 225 (94), 219 (19), 207 (32), 199 (17), 197 (18), 196 (100), 195 (17), 194 (29), 177 (30), 176 (100), 175 (45), 170 (22), 169 (22), 151 (49), 150 (40), 126 (19), 123 (43), 98 (77), 85 (20), 75 (23), 74 (17).

Chem. Name	1-methyl-2-(phenylethynyl)benzene (3k)
Lit. Ref.	Appl Organomet Chem., 2024, 38, e7405.

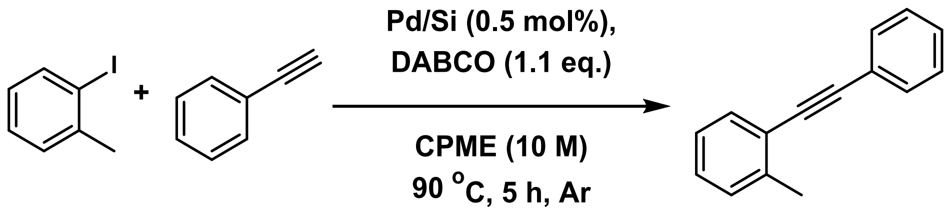

1e **2a** **3k**

M. W.: 192.26 g/mol

METHOD:

In a 2 mL screw-capped vial equipped with a magnetic stir bar, Pd/Si catalyst (5.7 wt%, 0.5 mol%, 9.3 mg), DABCO (1.1 equiv., 1.1 mmol, 123.4 mg), 1-iodo-2-methylbenzene **1e** (1 mmol, 218.0 mg, 127.3 µL), phenylacetylene **2e** (1.1 equiv., 1.1 mmol, 112.3 mg, 120.7 µL) and CPME (0.1 mL) were sequentially added. The mixture was purged with Ar and stirred at 90 °C in a heating module. After 5 h, the reaction mixture was cooled to room temperature and the catalyst was recovered by centrifugation (6000 rpm, 10 min), washing three times with fresh CPME. The organic phase was collected and extracted three times with an equal volume of water. The combined organic layers were concentrated under reduced pressure, and the residue was further purified by rotary evaporation under vacuum, affording 1-methyl-2-(phenylethynyl)benzene **(3k)** as a liquid in 94% isolated yield (180.4 mg).

Mol Formula	$C_{15}H_{12}$	m.p.	-	
^{1}H NMR 400 MHz $CDCl_3$	δ value	No. H	Mult.	j value/Hz
	7.64-7.60	2	m	-
	7.58	1	d	7.3
	7.43-7.37	3	m	-
	7.30	2	d	3.8
	7.26-7.20	1	m	-
	2.64	3	s	-

^{13}C NMR (100.6 MHz, $CDCl_3$) δ: 140.25, 131.93, 131.60, 129.56, 128.44, 128.40, 128.26, 125.68, 123.64, 123.11, 93.47, 88.47, 20.85.

GC-EIMS (m/z, %):193 (15), 192 (100), 191 (95), 190 (19), 189 (35), 165 (28)

**Chem. Name**	**1-(tert-butyl)-4-(phenylethynyl)benzene (3l)**
**Lit. Ref.**	Catal. Commun., 2015,69, 11-15.

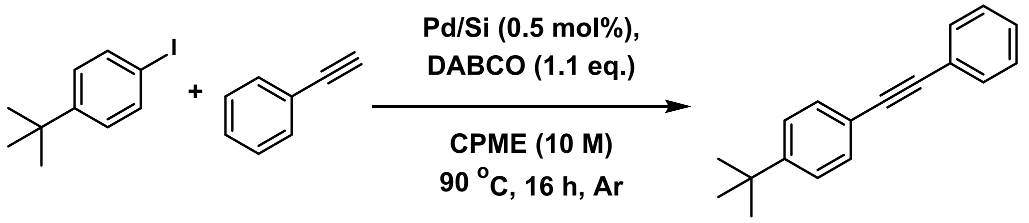

1f **2a** **3l**

M. W.: 234.34 g/mol

METHOD:
In a 2 mL screw-capped vial equipped with a magnetic stir bar, Pd/Si catalyst (5.7 wt%, 0.5 mol%, 9.3 mg), DABCO (1.1 equiv., 1.1 mmol, 123.4 mg), 1-(tert-butyl)-4-iodobenzene **1f** (1 mmol, 260.1 mg), phenylacetylene **2a** (1.1 equiv., 1.1 mmol, 112.3 mg, 120.7 µL) and CPME (0.1 mL) were sequentially added. The mixture was purged with Ar and stirred at 90 °C in a heating module. After 16 h, the reaction mixture was cooled to room temperature and the catalyst was recovered by centrifugation (6000 rpm, 10 min), washing three times with fresh CPME. The organic phase was collected and extracted three times with an equal volume of water. The combined organic layers were concentrated under reduced pressure, and the residue was further purified by rotary evaporation under vacuum, affording 1-(tert-butyl)-4-(phenylethynyl)benzene **(3l)** as a white solid in 81% isolated yield (188.8 mg).

**Mol Formula**	$C_{18}H_{18}$	**m.p.**	63-64 °C	
**^{1}H NMR 400 MHz $CDCl_3$**	**δ value**	**No. H**	**Mult.**	**j value/Hz**
	7.57	2	dd	7.6, 1.9
	7.52	2	d	8.4
	7.41	2	d	8.4
	7.38-7.34	3	m	-
	1.37	9	s	-

^{13}C NMR (100.6 MHz, $CDCl_3$) δ: 151.56, 131.63, 131.40, 131.38, 128.36, 128.11, 125.40, 123.57, 120.31, 89.61, 88.81, 31.24.

GC-EIMS (m/z, %): 236 (68), 235 (100), 234 (100), 221 (94), 220 (100), 219 (100), 217 (70), 216 (50), 215 (100), 213 (29), 205 (100), 204 (100), 203 (100), 202 (100), 201 (100), 200 (99), 192 (100), 191 (100), 190 (100), 189 (100), 187 (28); 180 (51), 179 (100), 178 (100), 177 (100), 176 (100), 175 (47), 174 (29), 166 (23), 165 (100), 164 (38), 163 (63), 153 (30), 152 (100), 151 (100), 150 (100), 142 (27), 141 (100), 142 (27), 141 (100); 139 (77), 137 (20), 128 (31), 127 (73), 126 (79), 117 (23), 116 (28), 115 (10), 113 (25), 110 (100), 108 (22), 103 (52), 102 (61), 101 (100), 100 (44), 99 (23), 98 (22), 96 (100), 95 (100), 91 (100), 89 (57), 88 (73), 87 (39), 77 (88), 76 (47), 75 (48), 74 (29), 65 (29), 63 (48), 57 (42), 51 (54), 50 (21), 29 (26), 27 (29).

Chem. Name	**1-methoxy-3-(phenylethynyl)benzene (3m)**
**Lit. Ref.**	Appl Organomet Chem., 2024, 38, e7405.

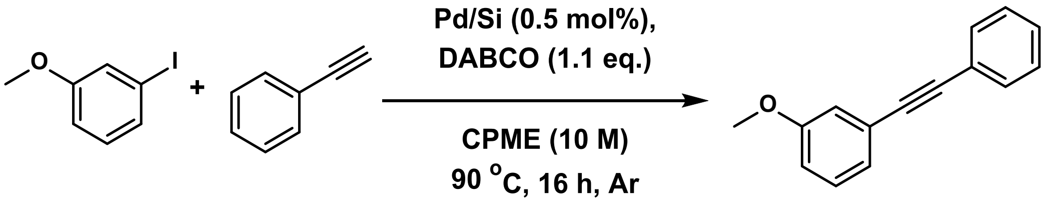

1g **2a** **3m**

M. W.: 208.26 g/mol

METHOD:

In a 2 mL screw-capped vial equipped with a magnetic stir bar, Pd/Si catalyst (5.7 wt%, 0.5 mol%, 9.3 mg), DABCO (1.1 equiv., 1.1 mmol, 123.4 mg), 1-iodo-3-methoxybenzene **1g** (1 mmol, 234.0 mg, 119.1 μL), phenylacetylene **2a** (1.1 equiv., 1.1 mmol, 112.3 mg, 120.7 μL) and CPME (0.1 mL) were sequentially added. The mixture was purged with Ar and stirred at 90 °C in a heating module. After 16 h, the reaction mixture was cooled to room temperature and the catalyst was recovered by centrifugation (6000 rpm, 10 min), washing three times with fresh CPME. The organic phase was collected and extracted three times with an equal volume of water. The combined organic layers were concentrated under reduced pressure, and the residue was further purified by rotary evaporation under vacuum, affording 1-methoxy-3-(phenylethynyl)benzene **(3m)** as a white solid in 94% isolated yield (195.4 mg).

**Mol Formula**	$C_{15}H_{12}O$	**m.p.**	77-78 °C	
**^{1}H NMR 400 MHz $CDCl_3$**	**δ value**	**No. H**	**Mult.**	**j value/Hz**
	7.63 – 7.51	2	m	-
	7.37	3	m	-
	7.29	1	d	7.8
	7.17	1	d	7.6
	7.10	1	bs	-
	6.92	1	dd	8.3, 1.7
	3.84	3	s	-

^{13}C NMR (100.6 MHz, $CDCl_3$) δ: 159.40, 131.67, 129.46, 128.40, 128.35, 124.22, 123.23, 116.38, 114.99, 113.80, 89.38, 89.26, 55.31.

GC-EIMS (m/z, %): 209 (17), 208 (100), 178 (28), 165 (30), 164 (13), 163 (13).

Chem. Name	**1-(phenylethynyl)naphthalene (3n)**
**Lit. Ref.**	ACS Omega., 2023, 8, 16395–16410.

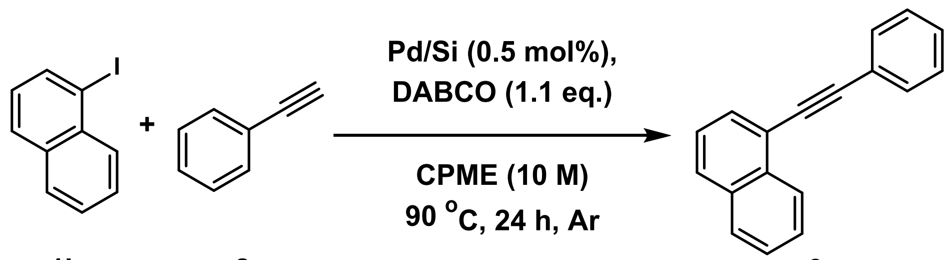

1h **2a** **3n**

M. W.: 228.29 g/mol

METHOD:

In a 2 mL screw-capped vial equipped with a magnetic stir bar, Pd/Si catalyst (5.7 wt%, 0.5 mol%, 9.3 mg), DABCO (1.1 equiv., 1.1 mmol, 123.4 mg), 1-iodonaphthalene **1h** (1 mmol, 254.1 mg, 146.0 µL), phenylacetylene **2a** (1.1 equiv., 1.1 mmol, 112.3 mg, 120.7 µL) and CPME (0.1 mL) were sequentially added. The mixture was purged with Ar and stirred at 90 °C in a heating module. After 24 h, the reaction mixture was cooled to room temperature and the catalyst was recovered by centrifugation (6000 rpm, 10 min), washing three times with fresh CPME. The organic phase was collected and extracted three times with an equal volume of water. The combined organic layers were concentrated under reduced pressure, and the residue was further purified by rotary evaporation under vacuum, affording 1-(phenylethynyl)naphthalene **(3n)** as a liquid in 70% isolated yield (144.8 mg).

**Mol Formula**	$C_{18}H_{12}$	**m.p.**	-	
	**δ value**	**No. H**	**Mult.**	**j value/Hz**
**^{1}H NMR 400 MHz $CDCl_3$**	8.57	1	d	8.3
	7.95-7.83	3	m	-
	7.58	2	d	5.9
	7.71-7.66	1	m	-
	7.63-7.57	1	m	-
	7.55-7.51	1	d	7.7
	7.51-7.40	3	m	-

^{13}C NMR (100.6 MHz, $CDCl_3$) δ: 137.52, 133.40, 133.33, 131.79, 130.50, 128.89, 128.56, 128.51, 128.45, 126.91, 126.55, 126.34, 125.40, 123.53, 94.50, 87.73.

GC-EIMS (m/z, %): 230 (100), 229 (100), 225 (100), 224 (30), 222 (30), 213 (42), 211 (25), 203 (100), 202 (100), 201 (100), 200 (100), 199 (94), 198 (63), 189 (88), 188 (70), 187 (100), 186 (33), 177 (26), 176 (100), 175 (87), 174 (100), 164 (26), 163 (93), 162 (37), 161 (28), 152 (100), 151 (100), 150 (100) 149 (44), 139 (30), 114 (100), 113 (100), 111 (41), 110 (30), 101 (100); 100 (100), 99 (67), 98 (57), 89 (32), 88 (100); 87 (95), 86 (43), 77 (33), 76 (64), 75 (85), 74 (76), 63 (83), 62 (34), 52 (28), 51 (67), 50 (54), 39 (64).

Chem. Name	Ethyl 4-(phenylethynyl)benzoate (3o)
Lit. Ref.	ACS Omega., 2023, 8, 16395–16410.

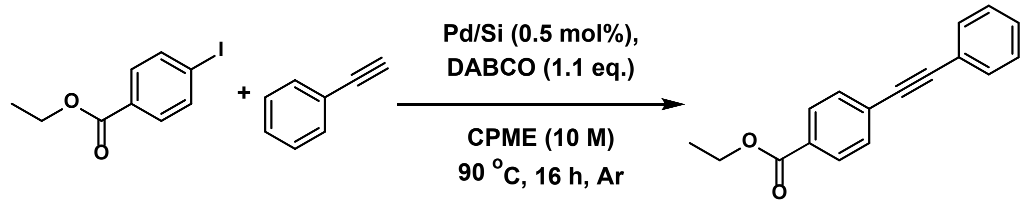

1i **2a** **3o**

M. W.: 250.30 g/mol

METHOD:
In a 2 mL screw-capped vial equipped with a magnetic stir bar, Pd/Si catalyst (5.7 wt%, 0.5 mol%, 9.3 mg), DABCO (1.1 equiv., 1.1 mmol, 123.4 mg), ethyl 4-iodobenzoate **1i** (1 mmol, 276.1 mg, 168.2 µL), phenylacetylene **2a** (1.1 equiv., 1.1 mmol, 112.3 mg, 120.7 µL) and CPME (0.1 mL) were sequentially added. The mixture was purged with Ar and stirred at 90 °C in a heating module. After 16 h, the reaction mixture was cooled to room temperature and the catalyst was recovered by centrifugation (6000 rpm, 10 min), washing three times with fresh CPME. The organic phase was collected and extracted three times with an equal volume of water. The combined organic layers were concentrated under reduced pressure, and the residue was further purified by rotary evaporation under vacuum, affording Ethyl 4-(phenylethynyl)benzoate **(3o)** as a yellow solid in 93% isolated yield (232.7 mg).

**Mol Formula**	$C_{17}H_{14}O_2$	**m.p.**	77-79 °C	
	**δ value**	**No. H**	**Mult.**	**j value/Hz**
**^{1}H NMR 400 MHz $CDCl_3$**	8.03	2	d	8.3
	7.63 – 7.53	4	m	-
	7.40 – 7.33	3	m	-
	4.39	2	q	7.1
	1.41	3	t	7.1

^{13}C NMR (100.6 MHz, $CDCl_3$) δ: 166.1, 131.5, 129.9, 129.5, 128.8, 128.5, 128.0, 122.8, 92.3, 88.7, 60.6, 14.3.

GC-EIMS (m/z, %): 252 (21), 251 (100), 250 (100), 186 (63), 185 (100), 171 (22), 170 (100), 169 (100), 168 (100), 145 (58), 144 (100), 143 (100), 142 (54), 141 (27), 120 (33), 119 (100); 118 (100), 127 (24), 126 (49), 103 (34), 88 (71), 77 (25), 75 (29), 51 (20), 29 (36), 27 (25).

Chem. Name	**1-ethyl-4-(p-tolylethynyl)benzene (3p)**
**Lit. Ref.**	ChemCatChem., 2018, 10, 758.

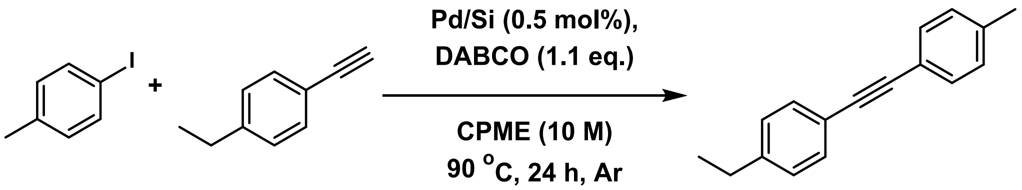

1j **2e** **3p**

M. W.: 220.32 g/mol

METHOD:
In a 2 mL screw-capped vial equipped with a magnetic stir bar, Pd/Si catalyst (5.7 wt%, 0.5 mol%, 9.3 mg), DABCO (1.1 equiv., 1.1 mmol, 123.4 mg), 1-iodo-4-methylbenzene **1j** (1 mmol, 218.0 mg), 1-ethyl-4-ethynylbenzene **2e** (1.1 equiv., 1.1 mmol, 143.2 mg, 154.0 μL) and CPME (0.1 mL) were sequentially added. The mixture was purged with Ar and stirred at 90 °C in a heating module. After 24 h, the reaction mixture was cooled to room temperature and the catalyst was recovered by centrifugation (6000 rpm, 10 min), washing three times with fresh CPME. The organic phase was collected and extracted three times with an equal volume of water. The combined organic layers were concentrated under reduced pressure, and the residue was further purified by rotary evaporation under vacuum, affording 1-ethyl-4-(p-tolylethynyl)benzene **(3p)** as a liquid in 82% isolated yield (180.6 mg).

**Mol Formula**	$C_{17}H_{16}$	**m.p.**	-	
**^{1}H NMR 400 MHz $CDCl_3$**	**δ value**	**No. H**	**Mult.**	**j value/Hz**
	7.49-7.41	4	m	-
	7.22-7.15	4	m	-
	2.68	2	q	7.6
	2.39	3	s	-
	1.27	3	t	7.6

^{13}C NMR (100.6 MHz, $CDCl_3$) δ: 144.49, 138.16, 131.53, 131.45, 129.09, 127.89, 120.64, 120.43, 88.91, 88.87, 28.83, 21.50, 15.36.

GC-EIMS (m/z, %): 221 (100), 220 (100), 206 (100), 205 (100), 204 (63), 203 (83), 202 (100), 191 (21), 190 (40), 189 (100), 178 (51), 176 (25), 165 (46), 163 (23), 152 (27), 139 (32), 115 (25), 102 (47), 101 (30), 89 (29)

Chem. Name	1-bromo-4-((4-chlorophenyl)ethynyl)benzene (3q)
Lit. Ref.	Synthesis., 2018, 50, 3197-3204.

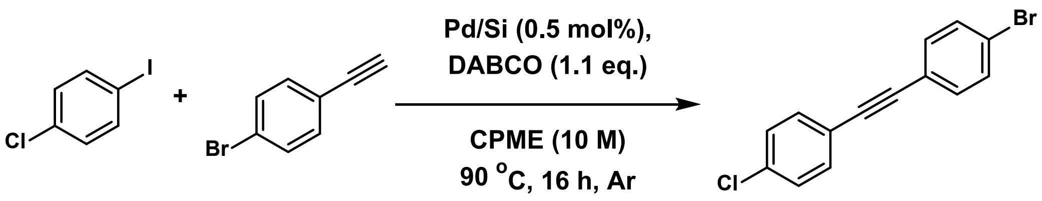

1h 2b 3q

M. W.: 291.6 g/mol

METHOD:

In a 2 mL screw-capped vial equipped with a magnetic stir bar, Pd/Si catalyst (5.7 wt%, 0.5 mol%, 9.3 mg), DABCO (1.1 equiv., 1.1 mmol, 123.4 mg), 1-chloro-4-iodobenzene **1h** (1 mmol, 238.5 mg), 1-bromo-4-ethynylbenzene **2b** (1.1 equiv., 1.1 mmol, 199.1 mg) and CPME (0.1 mL) were sequentially added. The mixture was purged with Ar and stirred at 90 °C in a heating module. After 16 h, the reaction mixture was cooled to room temperature and the catalyst was recovered by centrifugation (6000 rpm, 10 min), washing three times with fresh CPME. The organic phase was collected and extracted three times with an equal volume of water. The combined organic layers were concentrated under reduced pressure, and the residue was further purified by rotary evaporation under vacuum, affording 1-bromo-4-((4-chlorophenyl)ethynyl)benzene **(3q)** as a yellow solid in 66% isolated yield (192.4 mg).

Mol Formula	$C_{14}H_8BrCl$	m.p.	176-179 °C	
^{1}H NMR 400 MHz $CDCl_3$	δ value	No. H	Mult.	j value/Hz
	7.51	2	d	8.38
	7.47	2	d	8.38
	7.40	2	d	8.55
	7.35	2	d	8.55

^{13}C NMR (100.6 MHz, $CDCl_3$) δ: 134.57, 133.00, 132.80, 131.69, 128.77, 122.76, 121.91, 121.43, 89.36, 89.24.

GC-EIMS (m/z, %): 294 (100), 293 (69), 292 (100), 291 (54). 290 (100), 177 (39), 176 (100), 175 (48), 174 (34), 150 (49), 149 (21), 146 (34), 145 (25), 88 (52), 87 (22), 75 (32), 18 (30).

Chem. Name	Ethyl 4-(p-tolylethynyl)benzoate (3r)
Lit. Ref.	Eur. J. Org. Chem., 2022, e202200359.

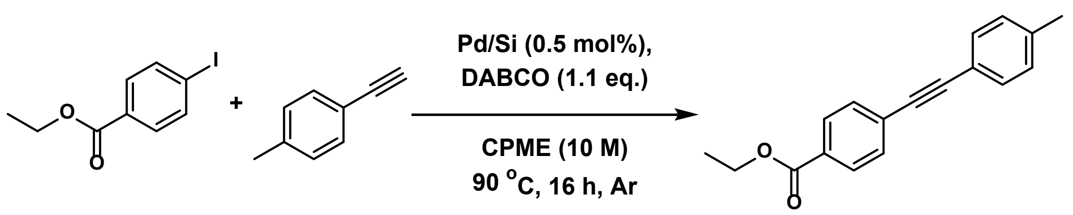

1i 2c 3r

M. W.: 264.3 g/mol

METHOD:
In a 2 mL screw-capped vial equipped with a magnetic stir bar, Pd/Si catalyst (5.7 wt%, 0.5 mol%, 9.3 mg), DABCO (1.1 equiv., 1.1 mmol, 123.4 mg), ethyl 4-iodobenzoate **1i** (1 mmol, 276.1 mg, 168.2 µL), 1-ethynyl-4-methylbenzene **2c** (1.1 equiv., 1.1 mmol, 127.8 mg, 139.4 µL) and CPME (0.1 mL) were sequentially added. The mixture was purged with Ar and stirred at 90 °C in a heating module. After 16 h, the reaction mixture was cooled to room temperature and the catalyst was recovered by centrifugation (6000 rpm, 10 min), washing three times with fresh CPME. The organic phase was collected and extracted three times with an equal volume of water. The combined organic layers were concentrated under reduced pressure, and the residue was further purified by rotary evaporation under vacuum, affording Ethyl 4-(p-tolylethynyl)benzoate **(3r)** as a yellow solid in 99% isolated yield (261.6 mg).

**Mol Formula**	$C_{18}H_{16}O_2$	**m.p.**	71-72 °C	
**^{1}H NMR 400 MHz $CDCl_3$**	**δ value**	**No. H**	**Mult.**	**j value/Hz**
	8.01	2	d	8.56
	7.57	2	d	8.56
	7.44	2	d	8.00
	7.17	2	d	8.00
	4.41	2	q	7.06
	2.40	3	s	-
	1.43	3	t	7.08

^{13}C NMR (100.6 MHz, $CDCl_3$) δ: 139.99, 131.65, 131.39, 130.21, 129.48, 129.22, 128.13, 119.67, 92.58, 87.76, 61.12, 21.01, 14.88.

GC-EIMS (m/z, %): 265 (100), 264 (100), 43 (237), 236 (100), 235 (31), 220 (100), 219 (100), 192 (32), 191 (100), 190 (100), 189 (100), 188 (26), 187 (21), 176 (97), 165 (74), 164 (25), 163 (42), 152 (24), 150 (19), 139 (33), 115 (24), 110 (47), 95 (58), 83 (36).

Chem. Name	1-chloro-4-((4-methoxyphenyl)ethynyl)benzene (3s)
Lit. Ref.	ACS Catal., 2019, 9, 3730–3736

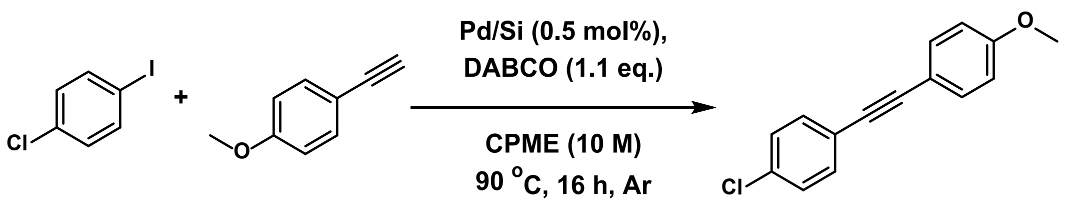

1h **2d** **3s**

M. W.: 242.70 g/mol

METHOD:

In a 2 mL screw-capped vial equipped with a magnetic stir bar, Pd/Si catalyst (5.7 wt%, 0.5 mol%, 9.3 mg), DABCO (1.1 equiv., 1.1 mmol, 123.4 mg), 1-chloro-4-iodobenzene **1h** (1 mmol, 238.5 mg), 1-ethynyl-4-methoxybenzene **2d** (1.1 equiv., 1.1 mmol, 145.4 mg, 142.7 μL) and CPME (0.1 mL) were sequentially added. The mixture was purged with Ar and stirred at 90 °C in a heating module. After 16 h, the reaction mixture was cooled to room temperature and the catalyst was recovered by centrifugation (6000 rpm, 10 min), washing three times with fresh CPME. The organic phase was collected and extracted three times with an equal volume of water. The combined organic layers were concentrated under reduced pressure, and the residue was further purified by rotary evaporation under vacuum, affording 1-chloro-4-((4-methoxyphenyl)ethynyl)benzene **(3s)** as a yellow solid in 91% isolated yield (220.8 mg).

**Mol Formula**	$C_{15}H_{11}ClO$	**m.p.**	124-126 °C	
**^{1}H NMR 400 MHz $CDCl_3$**	**δ value**	**No. H**	**Mult.**	**j value/Hz**
	7.49-7.40	4	m	-
	7.31	2	d	8.6
	6.88	2	d	8.7
	3.83	3	s	-

^{13}C NMR (100.6 MHz, $CDCl_3$) δ: 161.10, 134.29, 133.08, 132.65, 129.01, 120.97, 115.03, 114.07, 90.37, 87.00, 55.32.

GC-EIMS (m/z, %): 245 (57), 244 (100), 243 (100), 242 (100), 230 (26), 229 (100), 228 (79), 227 (100), 201 (96), 200 (42), 199 (100), 176 (41), 175 (21), 173 (34), 165 (28), 164 (100), 163 (100), 162 (24), 149 (21), 138 (39), 137 (41), 122 (26), 121 (60), 88 (25), 87 (28).

**Chem. Name**	**((4-(tert-butyl)phenyl)ethynyl)triisopropylsilane (3t)**
**Lit. Ref.**	Chem. Commun., 2025, 61, 9051-9054

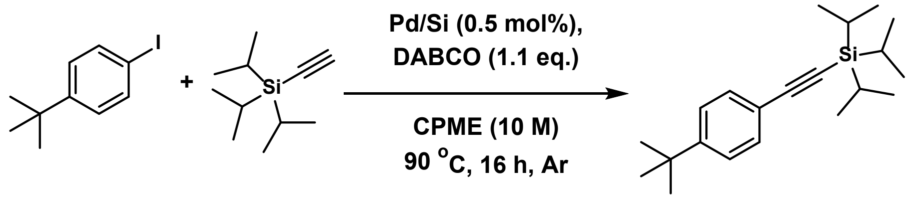

1g **2g** **3t**

M. W.: 314.59 g/mol

METHOD:

In a 2 mL screw-capped vial equipped with a magnetic stir bar, Pd/Si catalyst (5.7 wt%, 0.5 mol%, 9.3 mg), DABCO (1.1 eq., 1.1 mmol, 123.4 mg), 1-(tert-butyl)-4-iodobenzene **1g** (1 mmol, 260.1 mg), ethynyltriisopropylsilane **2g** (1.1 eq., 1.1 mmol, 200.62 mg, 246.8 μL) and CPME (0.1 mL) were sequentially added. The mixture was purged with Ar and stirred at 90 °C in a heating module. After 16 h, the reaction mixture was cooled to room temperature and the catalyst was recovered by centrifugation (6000 rpm, 10 min), washing three times with fresh CPME. The organic phase was collected and extracted three times with an equal volume of water. The combined organic layers were concentrated under reduced pressure, and the residue was further purified by rotary evaporation under vacuum, affording ((4-(tert-butyl)phenyl)ethynyl)triisopropylsilane **(3t)** as a yellow liquid in 91% isolated yield (286.2 mg).

**Mol Formula**	$C_{21}H_{34}Si$	**m.p.**	128-130°C	
**^{1}H NMR 400 MHz $CDCl_3$**	**δ value**	**No. H**	**Mult.**	**j value/Hz**
	7.46-7.42	2	m	-
	7.36-7.32	2	m	-
	1.33	9	s	-
	1.15	21	s	-

^{13}C NMR (100.6 MHz, $CDCl_3$) δ : 130.40, 126.25, 120.61, 108.37 90.45, 34.77, 31.17, 18.22, 12.26.

GC-EIMS (m/z, %): 314 (15), 271 (100), 257 (17), 229 (18), 186 (16), 129 (7), 43 (32)

Chem. Name	(Z)-3-benzylidene-2-methylisoindolin-1-one (5a)
Lit. Ref.	

Molecular Weight: 261.06

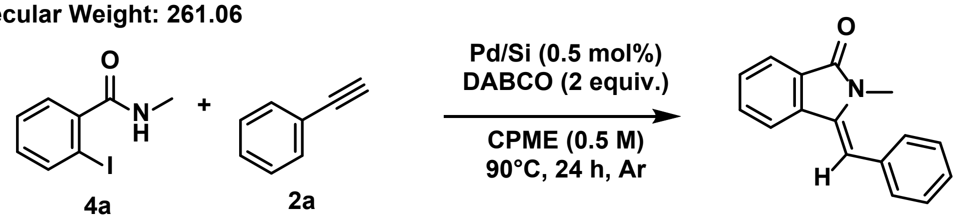

4a **2a** **1.1 equiv.**

5a
M. W.: 235.29 g/mol

METHOD:

In a 2 mL screw-capped vial equipped with a magnetic stir bar, Pd/Si catalyst (5.7 wt%, 0.5 mol%, 9.3 mg), DABCO (2 equiv., 2 mmol, 224.4 mg), 2-iodo-*N*-methylbenzamide **4a** (1 mmol, 261.1 mg), phenylacethylene **2a** (1.1 equiv., 1.1 mmol, 200.62 mg, 120.8 µL) and CPME (2 mL) were sequentially added. The mixture was purged with Ar and stirred at 90 °C in a heating module. After 24 h, the reaction mixture was cooled to room temperature and the catalyst was recovered by centrifugation (6000 rpm, 10 min), washing three times with fresh CPME. The organic phase was collected and extracted three times with an equal volume of water. The combined organic layers were concentrated under reduced pressure, and the residue was further purified by rotary evaporation under vacuum. Pure product was obtained by chromatographic purification as (Z)-3-benzylidene-2-methylisoindolin-1-one (**5a**) a solid in 73% isolated yield (171.76 mg).

Mol Formula		m.p.	129-130°C	
**^{1}H NMR 400 MHz $CDCl_3$**	**δ value**	**No. H**	**Mult.**	**j value/Hz**
	7.85	1	d	7.7
	7.74	1	d	7.7
	7.59	1	t	-
	7.50	1	t	-
	7.41-7.32	5	m	
	6.78	1	s	
	3.03	3	s	

^{13}C NMR (100.6 MHz, $CDCl_3$) δ: 169.09, 138.19, 136.30, 134.94, 132.05, 129.88, 129.15, 128.63, 128.28, 127.64, 123.31, 119.43, 106.74, 30.69.

GC-EIMS (m/z, %): 237 (47); 236 (100), 235 (100), 234 (100), 232 (100), 221 (22), 220 (100), 219 (70), 218 (47), 217 (36), 216 (90), 208 (37), 207 (100), 206 (100), 205 (73), 204 (100), 203 (22), 192 (62), 191 (83), 190 (10), 189 (36), 180 (38), 179 (100), 178 (100), 177 (100), 176 (100), 166 (83), 165 (100), 164 (80), 163 (56), 153 (22), 152 (84), 151 (65), 150 (37), 140 (23), 139 (48), 131 (22), 130(60), 129 (29), 128 (45), 126 (21) 117 (89), 116 (100), 115 (36), 91 (100), 90(99), 89 (100), 88 (37), 78 (28), 77 (100), 76 (100), 75 (58), 74 (31), 65 (26), 63 (71), 62 (22), 51 (55), 50 (49), 39 (41).

Chem. Name	1,2-diphenylethyne (3a)
Lit. Ref.	Green Chem., 2012, 14, 2840–2855

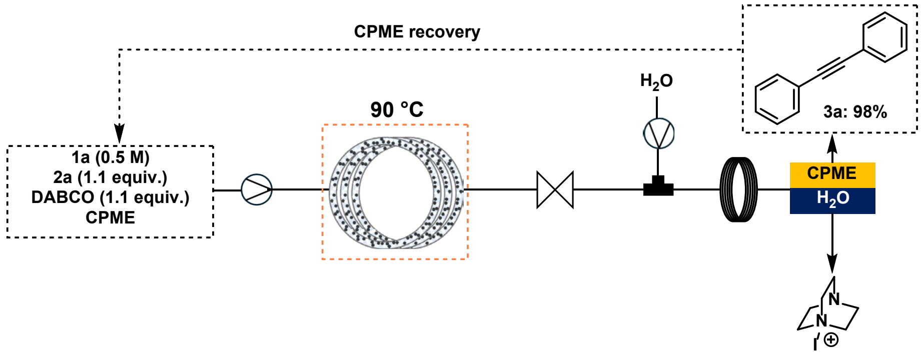

METHOD:

The continuous flow process was performed using two Shimadzu LC-20AD HPLC pump and a PTFE column (2.5 m, ID: ¼") fitted with HPLC peek connections. The reactor column was packed with 833 mg of Pd/Si (2.7 % wt) dispersed in 5.55 g of solid-glass beads (1.0 mm particle size). Iodobenzene **1a** (1 mmol) and phenylacetylene **2a** (1.1 mmol), and DABCO (1.1 mmol) were completely dissolved in CPME (0.5 M for iodobenzene). The line was directly inserted into the reactor column, thermostated at 90 °C, and equipped with a 250 psi BPR. The flow rate was 0.01 mL min^{-1}. Then a second channel was connected to pump water into the line. The two lines were connected via a T-junction, and then a loop (2 m, 1/16”) was added to help the mixing. The organic phase was separated using a Zaiput liquid/liquid separator. The water phase containing DABCO-I is directed to the waste disposal after water distillation. The organic phase (CPME) containing the products and the unreacted acetylenic compounds was directed to a continuous distillation apparatus. The CPME was recovered at 106-108 °C and, after NMR analysis to confirm the purity, was reused continuously. The crude reaction mixture for each compound was evaporated to remove the unreacted acetylenic compound, yielding the pure product **3a**. (Yield:98%)

**Mol Formula**	$C_{14}H_{10}$	**m.p.**	64-65 °C	
**^{1}H NMR**	**δ value**	**No. H**	**Mult.**	**j value/Hz**
**400 MHz**	7.60-7.53	4	m	-
**$CDCl_3$**	7.41-7.34	6	m	-

^{13}C NMR (100.6 MHz, $CDCl_3$) δ: 131.66, 128.39, 128.29, 123.33, 89.81.

GC-EIMS (m/z, %):179 (15), 178 (100), 176 (21)

Chem. Name	1-methoxy-4-(phenylethynyl)benzene (3d)
Lit. Ref.	Chem. Commun., 2025,61, 9960-9963

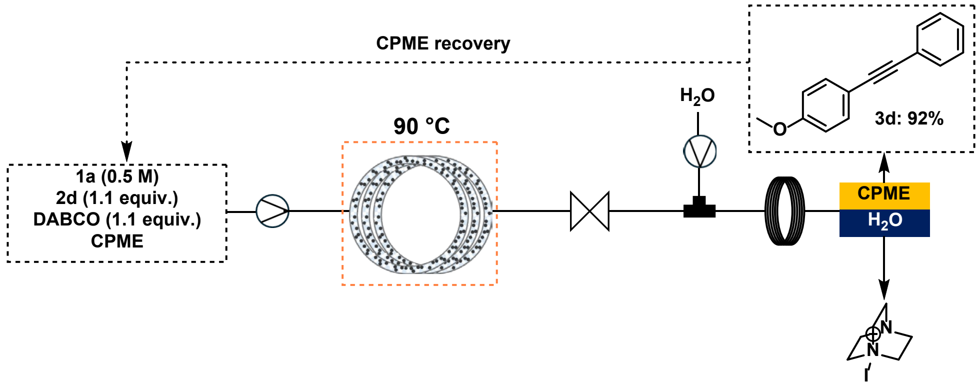

METHOD:

The continuous flow process was performed using two Shimadzu LC-20AD HPLC pump and a PTFE column (2.5 m, ID: ¼") fitted with HPLC peek connections. The reactor column was packed with 833 mg of Pd/Si (2.7 % wt) dispersed in 5.55 g of solid-glass beads (1.0 mm particle size). Iodobenzene **1a** (1 mmol) and 4-methoxyphenylacetylene **2d** (1.1 mmol), and DABCO (1.1 mmol) were completely dissolved in CPME (0.5 M for iodobenzene). The line was directly inserted into the reactor column, thermostated at 90 °C, and equipped with a 250 psi BPR. The flow rate was 0.01 mL min^{-1}. Then a second channel was connected to pump water into the line. The two lines were connected via a T-junction, and then a loop (2 m, 1/16") was added to help the mixing. The organic phase was separated using a Zaiput liquid/liquid separator. The water phase containing DABCO-I is directed to the waste disposal after water distillation. The organic phase (CPME) containing the products and the unreacted acetylenic compounds was directed to a continuous distillation apparatus. The CPME was recovered at 106-108 °C and, after NMR analysis to confirm the purity, was reused continuously. The crude reaction mixture for each compound was evaporated to remove the unreacted acetylenic compound, yielding the pure product **3d**. (Yield: 92%)

**Mol Formula**	$C_{15}H_{12}O$	**m.p.**	90-93°C	
**^{1}H NMR 400 MHz $CDCl_3$**	**δ value**	**No. H**	**Mult.**	**j value/Hz**
	7.58 – 7.45	4	m	-
	7.34	3	m	-
	6.89	2	d	8.8
	3.83	3	s	-

^{13}C NMR (100.6 MHz, $CDCl_3$) δ: 133.60, 133.07, 131.46, 128.32, 127.95, 115.38, 114.01, 113.97, 113.95, 88.08, 55.31.

GC-EIMS (m/z, %): 208 (100), 193 (10), 165 (50), 164 (18), 163 (13), 139 (13)

Chem. Name	1-ethyl-4-(phenylethynyl)benzene (3e)
Lit. Ref.	Appl Organomet Chem., 2024, 38, e7405.

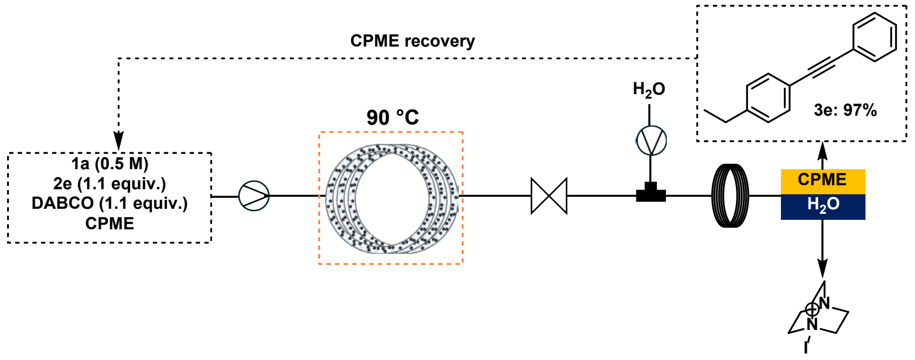

METHOD:

The continuous flow process was performed using two Shimadzu LC-20AD HPLC pump and a PTFE column (2.5 m, ID: ¼") fitted with HPLC peek connections. The reactor column was packed with 833 mg of Pd/Si (2.7 % wt) dispersed in 5.55 g of solid-glass beads (1.0 mm particle size). Iodobenzene **1a** (1 mmol) and 4-ethylphenylacetylene **2e** (1.1 mmol), and DABCO (1.1 mmol) were completely dissolved in CPME (0.5 M for iodobenzene). The line was directly inserted into the reactor column, thermostated at 90 °C, and equipped with a 250 psi BPR. The flow rate was 0.01 mL min^{-1}. Then a second channel was connected to pump water into the line. The two lines were connected via a T-junction, and then a loop (2 m, 1/16") was added to help the mixing. The organic phase was separated using a Zaiput liquid/liquid separator. The water phase containing DABCO-I is directed to the waste disposal after water distillation. The organic phase (CPME) containing the products and the unreacted acetylenic compounds was directed to a continuous distillation apparatus. The CPME was recovered at 106-108 °C and, after NMR analysis to confirm the purity, was reused continuously. The crude reaction mixture for each compound was evaporated to remove the unreacted acetylenic compound, yielding the pure product **3e**. (Yield: 97%)

**Mol Formula**	$C_{16}H_{14}$	**m.p.**	-	
	**δ value**	**No. H**	**Mult.**	**j value/Hz**
**^{1}H NMR 400 MHz $CDCl_3$**	7.56	2	m	-
	7.49	2	d	6
	7.36	3	m	-
	7.21	2	d	7
	2.69	2	q	7.5
	1.28	3	t	7.6

^{13}C NMR (100.6 MHz, $CDCl_3$) δ: 144.71, 131.64, 131.60, 128.36, 128.11, 127.97, 89.67, 88.79, 28.90, 28.88, 15.43, 15.41.

GC-EIMS (m/z, %): 208 (53), 207 (100), 206 (100), 204 (83), 203 (100), 202 (100), 201 (44), 200 (35), 193 (55), 192 (100), 191 (100), 190 (100), 189 (100), 187 (60), 179 (36), 178 (100), 177 (54), 176 (100), 175 (33), 174 (20), 166 (57), 165 (100), 164 (100), 163 (100), 162 (25), 153 (20), 152 (100), 151 (97), 150 (100), 139 (100), 138 (25), 137 (27), 128 (65), 127 (58), 126 (88), 115 (83), 113 (40), 111 (21), 103 (44) 102 (49), 101 (51), 100 (25), 99 (27), 98 (34), 96 (100), 95 (85), 91 (23), 89 (100), 88 (50), 87 (53), 86 (31), 83 (77), 78 (29), 77 (73), 76 (70), 75 (50), 74 (42), 65 (29), 63 (78), 62 (23), 52 (21), 51 (57), 50 (32), 39 (65).

Chem. Name	Triisopropyl(phenylethynyl)silane (3g)
Lit. Ref.	J. Org. Chem., 2023, 88, 11675–11682

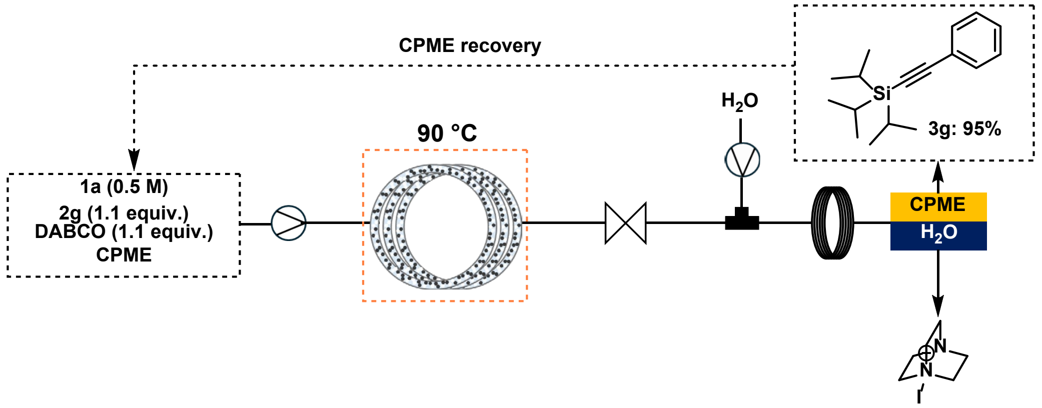

METHOD:

The continuous flow process was performed using two Shimadzu LC-20AD HPLC pump and a PTFE column (2.5 m, ID: ¼") fitted with HPLC peek connections. The reactor column was packed with 833 mg of Pd/Si (2.7 % wt) dispersed in 5.55 g of solid-glass beads (1.0 mm particle size). Iodobenzene **1a** (1 mmol) and (triisopropylsilyl)acetylene **2g** (1.1 mmol), and DABCO (1.1 mmol) were completely dissolved in CPME (0.5 M for iodobenzene). The line was directly inserted into the reactor column, thermostated at 90 °C, and equipped with a 250 psi BPR. The flow rate was 0.01 mL min^{-1}. Then a second channel was connected to pump water into the line. The two lines were connected via a T-junction, and then a loop (2 m, 1/16") was added to help the mixing. The organic phase was separated using a Zaiput liquid/liquid separator. The water phase containing DABCO-I is directed to the waste disposal after water distillation. The organic phase (CPME) containing the products and the unreacted acetylenic compounds was directed to a continuous distillation apparatus. The CPME was recovered at 106-108 °C and, after NMR analysis to confirm the purity, was reused continuously. The crude reaction mixture for each compound was evaporated to remove the unreacted acetylenic compound, yielding the pure product **3g**. (Yield: 95%)

Mol Formula	$C_{17}H_{26}Si$	m.p.	-	
^1H NMR 400 MHz $CDCl_3$	δ value	No. H	Mult.	j value/Hz
	7.57 – 7.42	2	m	-
	7.37 – 7.27	3	m	-
	1.16	21	s	-

^{13}C NMR (100.6 MHz, $CDCl_3$) δ: 132.05, 128.29, 128.18, 123.60, 107.15, 90.42, 18.70, 11.37.

GC-EIMS (m/z, %): 258 (17), 215 (100), 173 (37), 131 (22), 105 (7), 43 (42)

Chem. Name	1-chloro-4-(phenylethynyl)benzene (3h)
Lit. Ref.	J. Solid State Chem., 2024, 336, 124736.

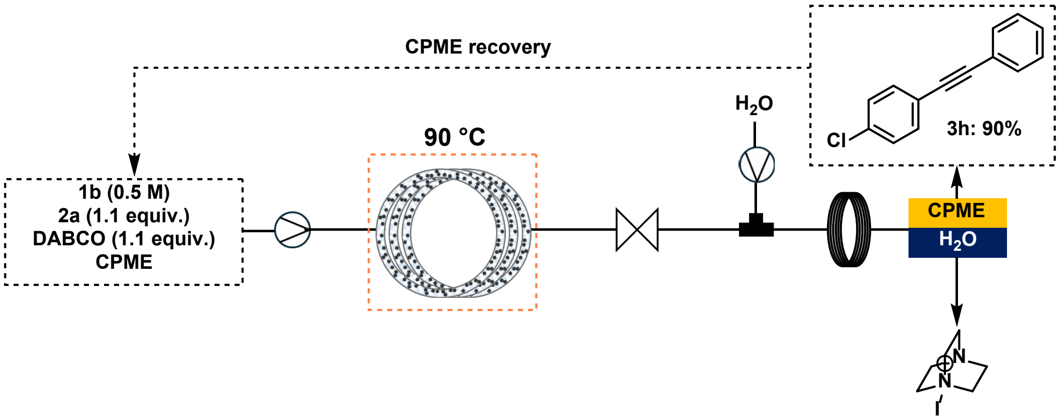

METHOD:

The continuous flow process was performed using two Shimadzu LC-20AD HPLC pumps and a PTFE column (2.5 m, ID: ¼") fitted with HPLC peak connections. The reactor column was packed with 833 mg of Pd/Si (2.7 % wt) dispersed in 5.55 g of solid-glass beads (1.0 mm particle size). 4-chloroiodobenzene **1b** (1 mmol) and phenylacetylene **2a** (1.1 mmol), and DABCO (1.1 mmol) were completely dissolved in CPME (0.5 M for iodobenzene). The line was directly inserted into the reactor column, thermostated at 90 °C, and equipped with a 250 psi BPR. The flow rate was 0.01 mL min^{-1}. Then a second channel was connected to pump water into the line. The two lines were connected via a T-junction, and then a loop (2 m, 1/16") was added to help the mixing. The organic phase was separated using a Zaiput liquid/liquid separator. The water phase containing DABCO-I is directed to the waste disposal after water distillation. The organic phase (CPME) containing the products and the unreacted acetylenic compounds was directed to a continuous distillation apparatus. The CPME was recovered at 106-108 °C and, after NMR analysis to confirm the purity, was reused continuously. The crude reaction mixture for each compound was evaporated to remove the unreacted acetylenic compound, yielding the pure product **3h**. (Yield: 90%)

**Mol Formula**	$C_{14}H_9Cl$	**m.p.**	110-111 °C	
**^{1}H NMR 400 MHz $CDCl_3$**	**δ value**	**No. H**	**Mult.**	**j value/Hz**
	7.59-7.54	2	m	-
	7.48	2	d	8.5
	7.40 – 7.32	5	m	-

^{13}C NMR (100.6 MHz, $CDCl_3$) δ: 134.30, 132.85, 131.65, 128.74, 128.53, 128.45, 122.98, 121.82, 90.40, 88.33.

GC-EIMS (m/z, %): 212 (100), 214 (33), 177 (25), 176 (17), 151 (47), 106 (12), 75 (4)

Chem. Name	1-(phenylethynyl)-4-(trifluoromethyl)benzene (3j)
Lit. Ref.	J. Solid State Chem., 2024, 336, 124736.

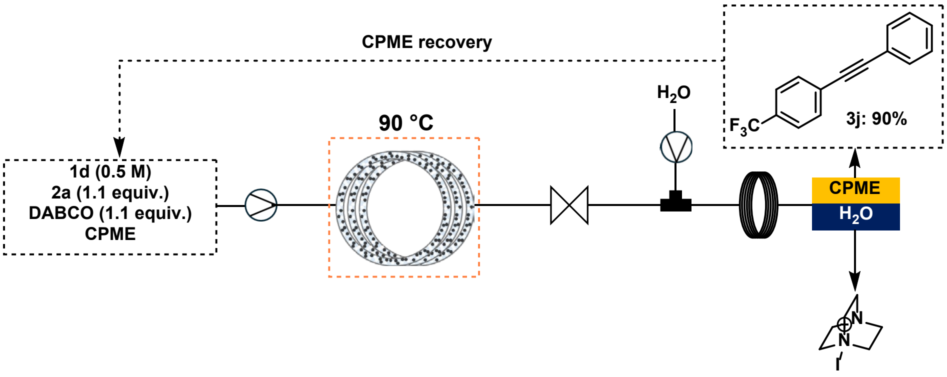

METHOD:

The continuous flow process was performed using two Shimadzu LC-20AD HPLC pumps and a PTFE column (2.5 m, ID: ¼") fitted with HPLC peak connections. The reactor column was packed with 833 mg of Pd/Si (2.7 % wt) dispersed in 5.55 g of solid-glass beads (1.0 mm particle size). 4-trifluormethyliodobenzene **1d** (1 mmol) and phenylacetylene **2a** (1.1 mmol), and DABCO (1.1 mmol) were completely dissolved in CPME (0.5 M for iodobenzene). The line was directly inserted into the reactor column, thermostated at 90 °C, and equipped with a 250 psi BPR. The flow rate was 0.01 mL min^{-1}. Then a second channel was connected to pump water into the line. The two lines were connected via a T-junction, and then a loop (2 m, 1/16”) was added to help the mixing. The organic phase was separated using a Zaiput liquid/liquid separator. The water phase containing DABCO-I is directed to the waste disposal after water distillation. The organic phase (CPME) containing the products and the unreacted acetylenic compounds was directed to a continuous distillation apparatus. The CPME was recovered at 106-108 °C and, after NMR analysis to confirm the purity, was reused continuously. The crude reaction mixture for each compound was evaporated to remove the unreacted acetylenic compound, yielding the pure product **3j**. (Yield:89%)

**Mol Formula**	$C_{15}H_9F_3$	**m.p.**	100-102°C	
**^{1}H NMR 400 MHz $CDCl_3$**	**δ value**	**No. H**	**Mult.**	**j value/Hz**
	7.67 – 7.59	4	m	-
	7.58-7.53	2	m	-
	7.41 – 7.35	3	m	-

^{13}C NMR (100.6 MHz, $CDCl_3$) δ: 131.81, 131.75, 130.09, 129.75, 128.84, 128.46, 127.15, 125.35, 125.31, 125.27, 125.24, 91.76, 87.97.

^{19}F NMR (376 MHz, CDCl3) δ: [-62.785].

GC-EIMS (m/z, %): 248 (19), 247(100), 246(100), 245 (85), 228 (22), 227 (100), 226 (19), 225 (94), 219 (19), 207 (32), 199 (17), 197 (18), 196 (100), 195 (17), 194 (29), 177 (30), 176 (100), 175 (45), 170 (22), 169 (22), 151 (49), 150 (40), 126 (19), 123 (43), 98 (77), 85 (20), 75 (23), 74 (17).

Chem. Name	1-chloro-4-((4-methoxyphenyl)ethynyl)benzene (3s)
Lit. Ref.	ACS Catal., 2019, 9, 3730–3736

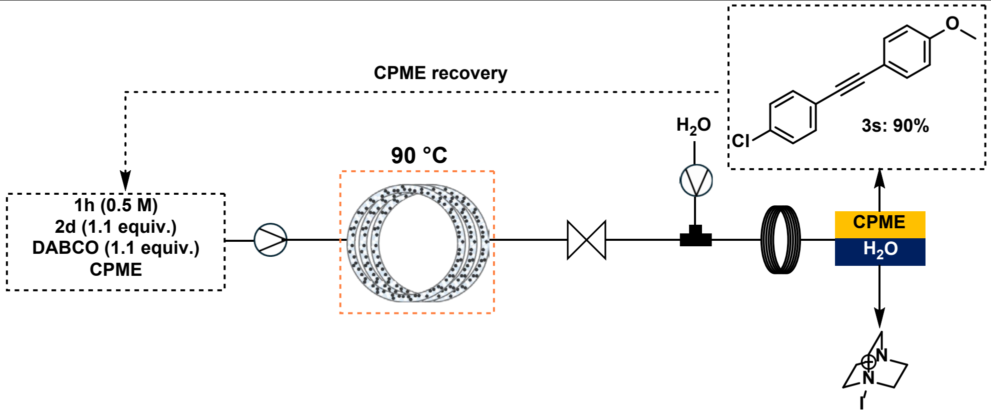

METHOD:

The continuous flow process was performed using two Shimadzu LC-20AD HPLC pump and a PTFE column (2.5 m, ID: ¼") fitted with HPLC peek connections. The reactor column was packed with 833 mg of Pd/Si (2.7 % wt) dispersed in 5.55 g of solid-glass beads (1.0 mm particle size). 4-chloro-Iodobenzene **1h** (1 mmol) and 4-methoxyphenylacetylene **2d** (1.1 mmol), and DABCO (1.1 mmol) were completely dissolved in CPME (0.5 M for iodobenzene). The line was directly inserted into the reactor column, thermostated at 90 °C, and equipped with a 250 psi BPR. The flow rate was 0.01 mL min^{-1}. Then a second channel was connected to pump water into the line. The two lines were connected via a T-junction, and then a loop (2 m, 1/16") was added to help the mixing. The organic phase was separated using a Zaiput liquid/liquid separator. The water phase containing DABCO-I is directed to the waste disposal after water distillation. The organic phase (CPME) containing the products and the unreacted acetylenic compounds was directed to a continuous distillation apparatus. The CPME was recovered at 106-108 °C and, after NMR analysis to confirm the purity, was reused continuously. The crude reaction mixture for each compound was evaporated to remove the unreacted acetylenic compound, yielding the pure product **3s**. (Yield:90%)

**Mol Formula**	$C_{15}H_{11}ClO$	**m.p.**	124-126 °C	
	**δ value**	**No. H**	**Mult.**	**j value/Hz**
**^{1}H NMR 400 MHz $CDCl_3$**	7.49-7.40	4	m	-
	7.31	2	d	8.6
	6.88	2	d	8.7
	3.83	3	s	-

^{13}C NMR (100.6 MHz, $CDCl_3$) δ: 161.10, 134.29, 133.08, 132.65, 129.01, 120.97, 115.03, 114.07, 90.37, 87.00, 55.32.

GC-EIMS (m/z, %): 245 (57), 244 (100), 243 (100), 242 (100), 230 (26), 229 (100), 228 (79), 227 (100), 201 (96), 200 (42), 199 (100), 176 (41), 175 (21), 173 (34), 165 (28), 164 (100), 163 (100), 162 (24), 149 (21), 138 (39), 137 (41), 122 (26), 121 (60), 88 (25), 87 (28).

Figure S3. ^{1}H-NMR and ^{13}C-NMR of 1,2-diphenylethyne (**3a**)

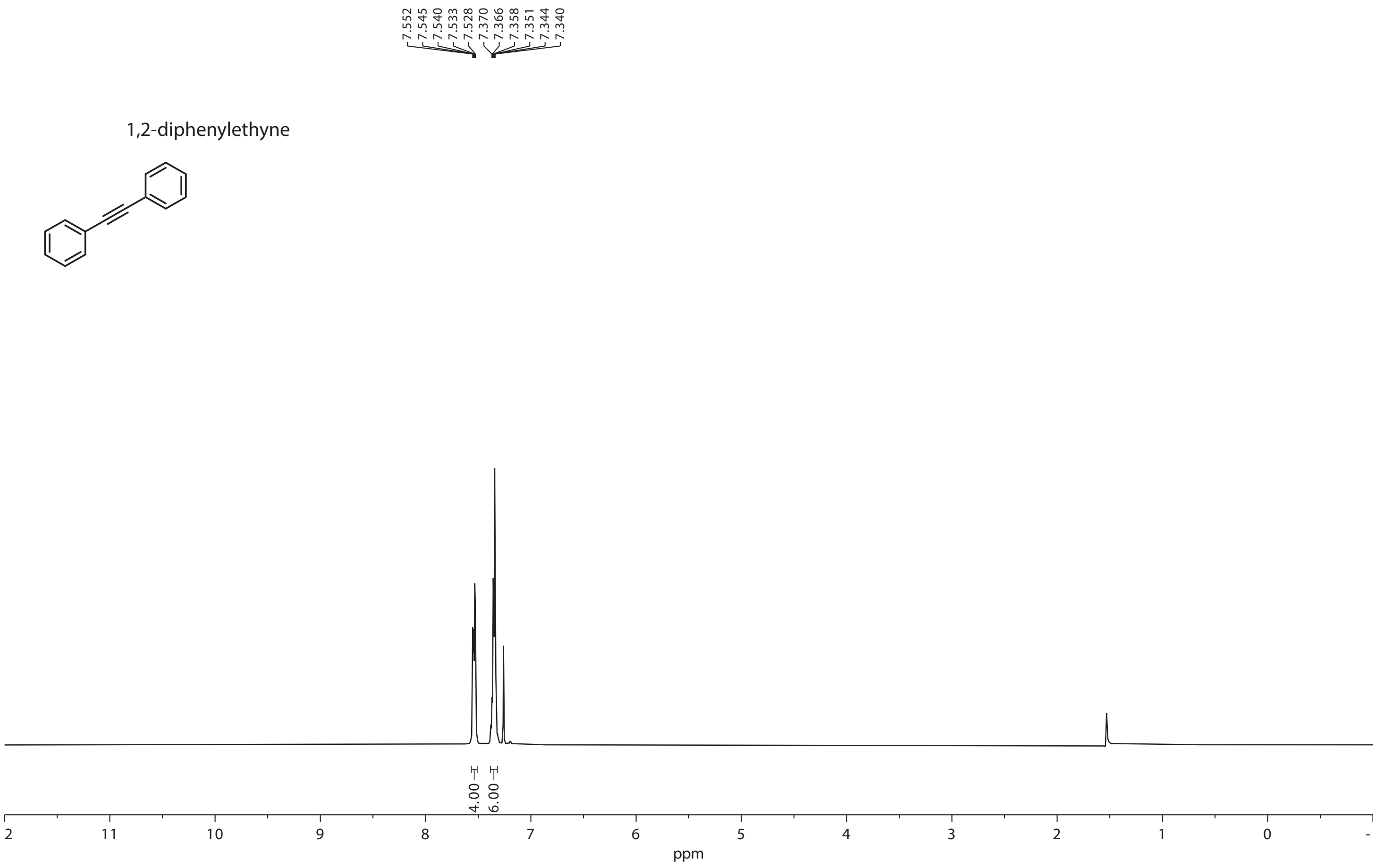

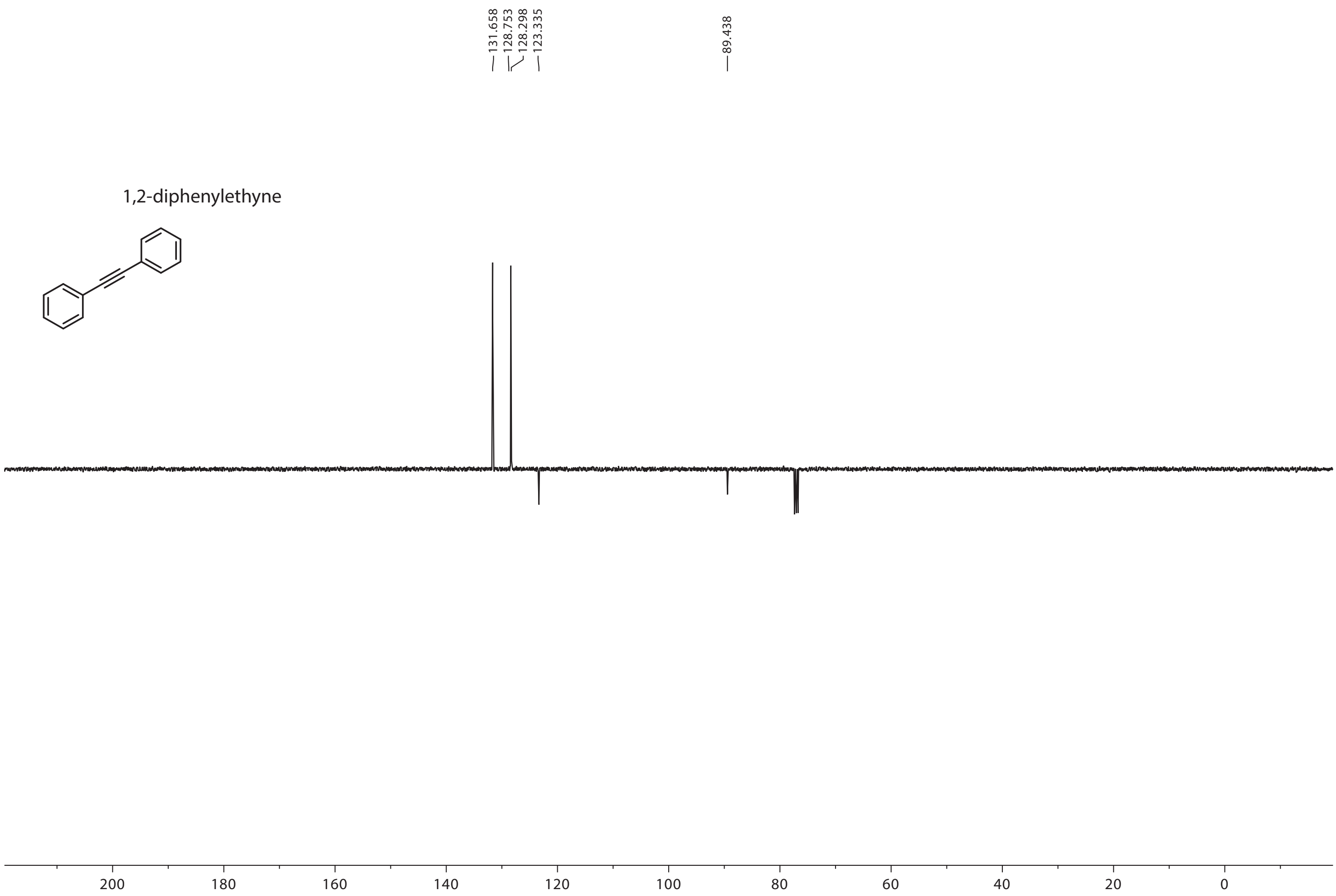

Figure S4. ^{1}H-NMR and ^{13}C-NMR of 1-bromo-4-(phenylethynyl)benzene (**3b**)

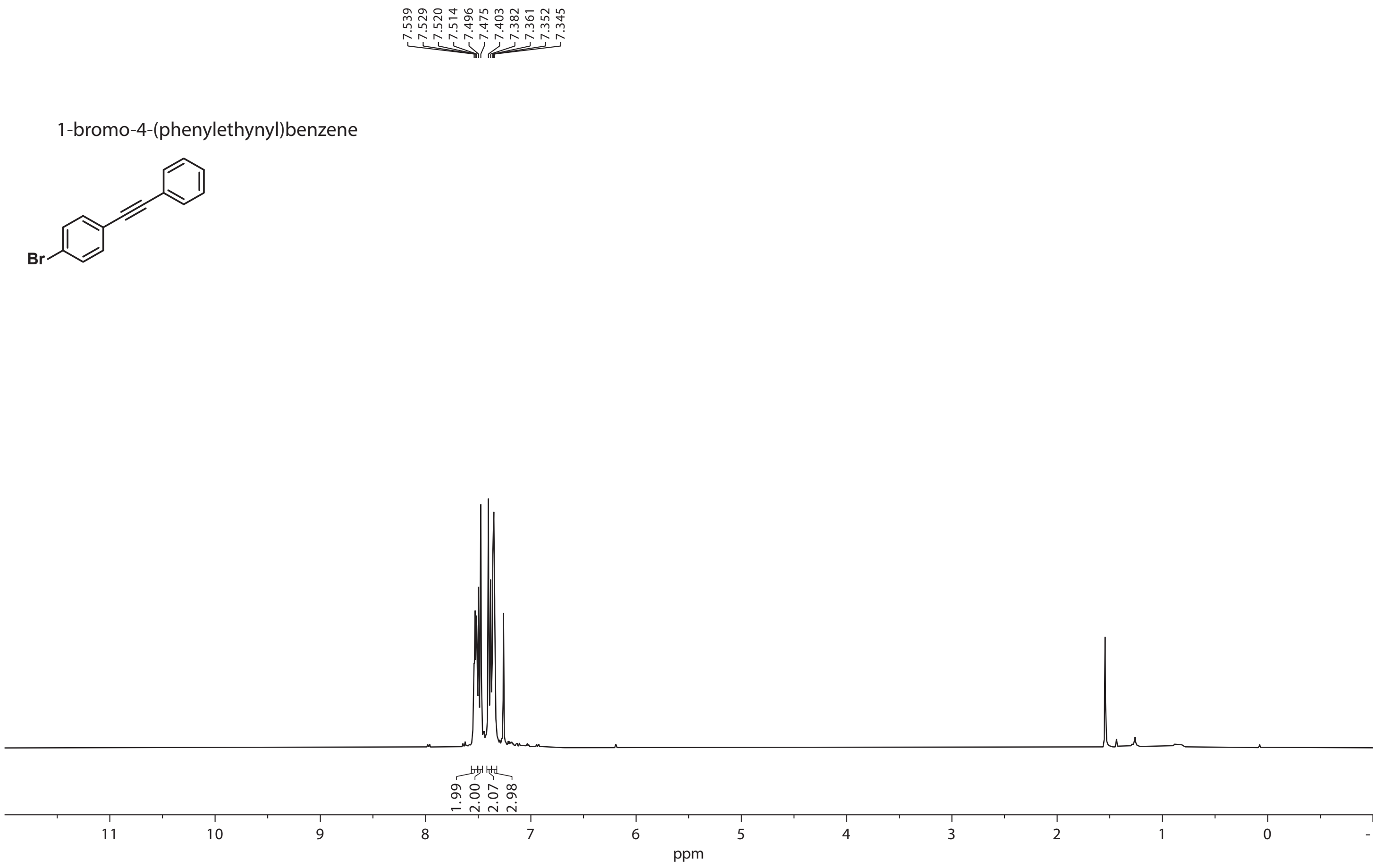

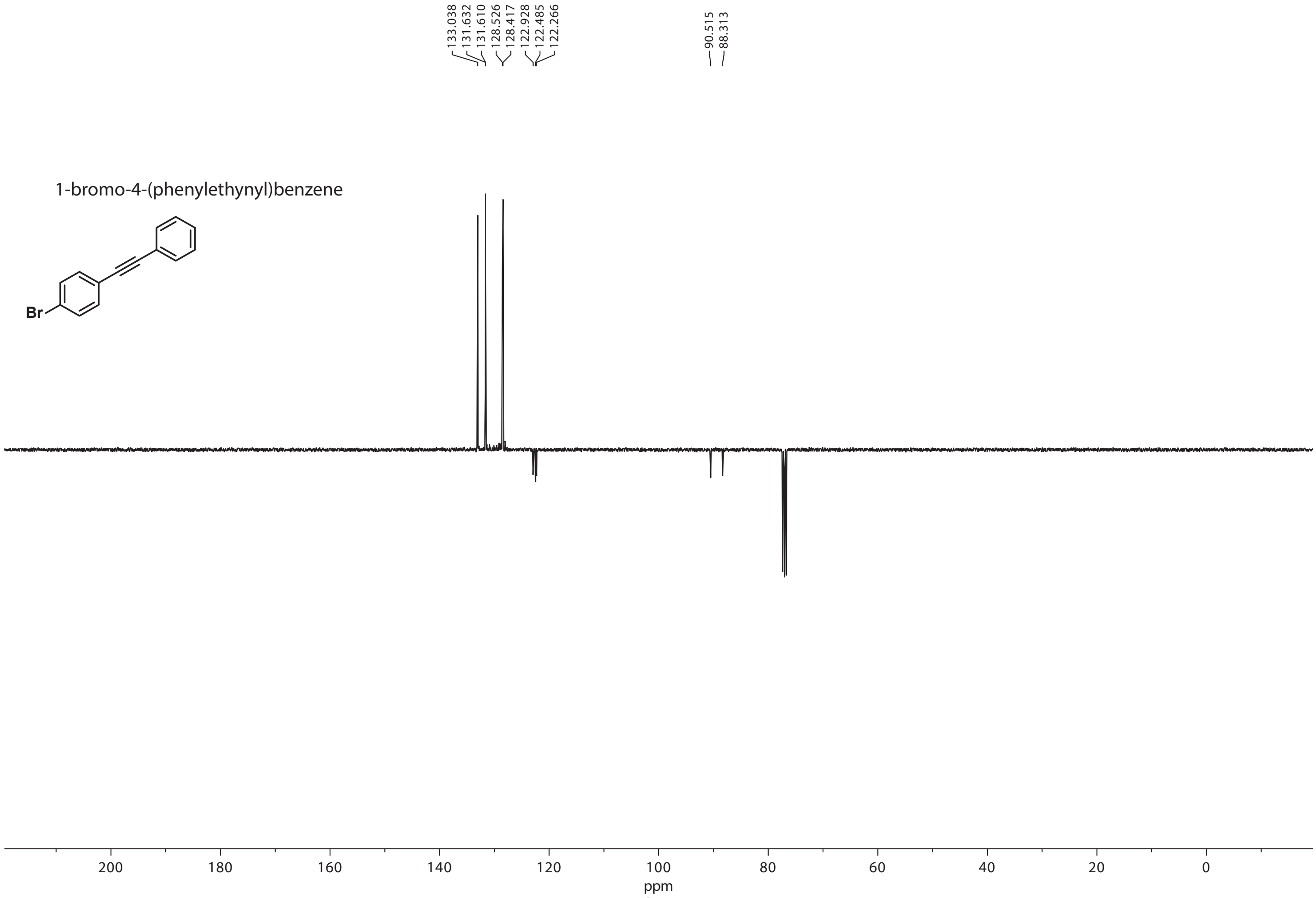

Figure S5. ¹H-NMR and ¹³C-NMR of 1-methyl-4-(phenylethynyl)benzene (**3c**)

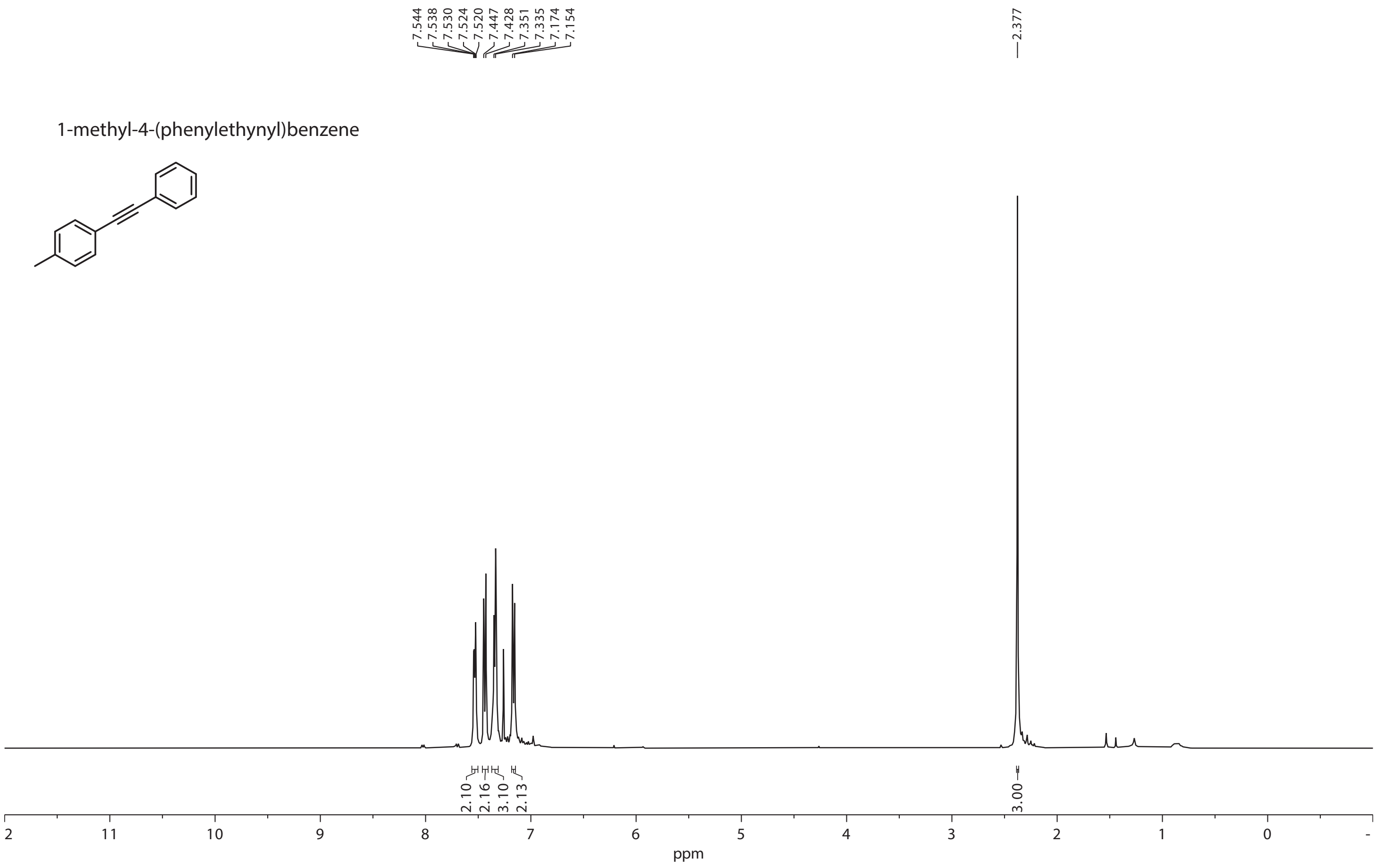

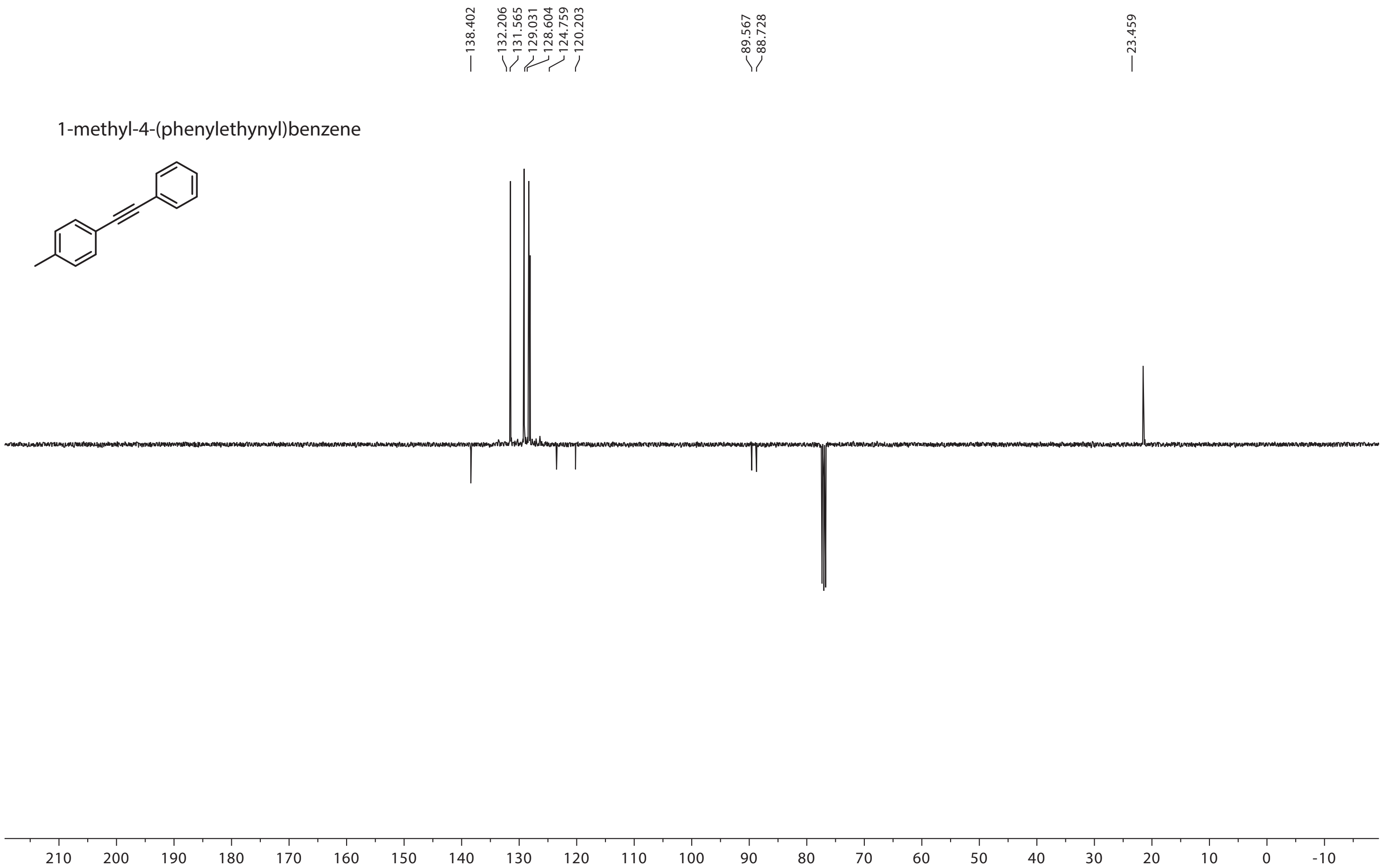

Figure S6. ^{1}H-NMR and ^{13}C-NMR of 1-methoxy-4-(phenylethynyl)benzene (**3d**)

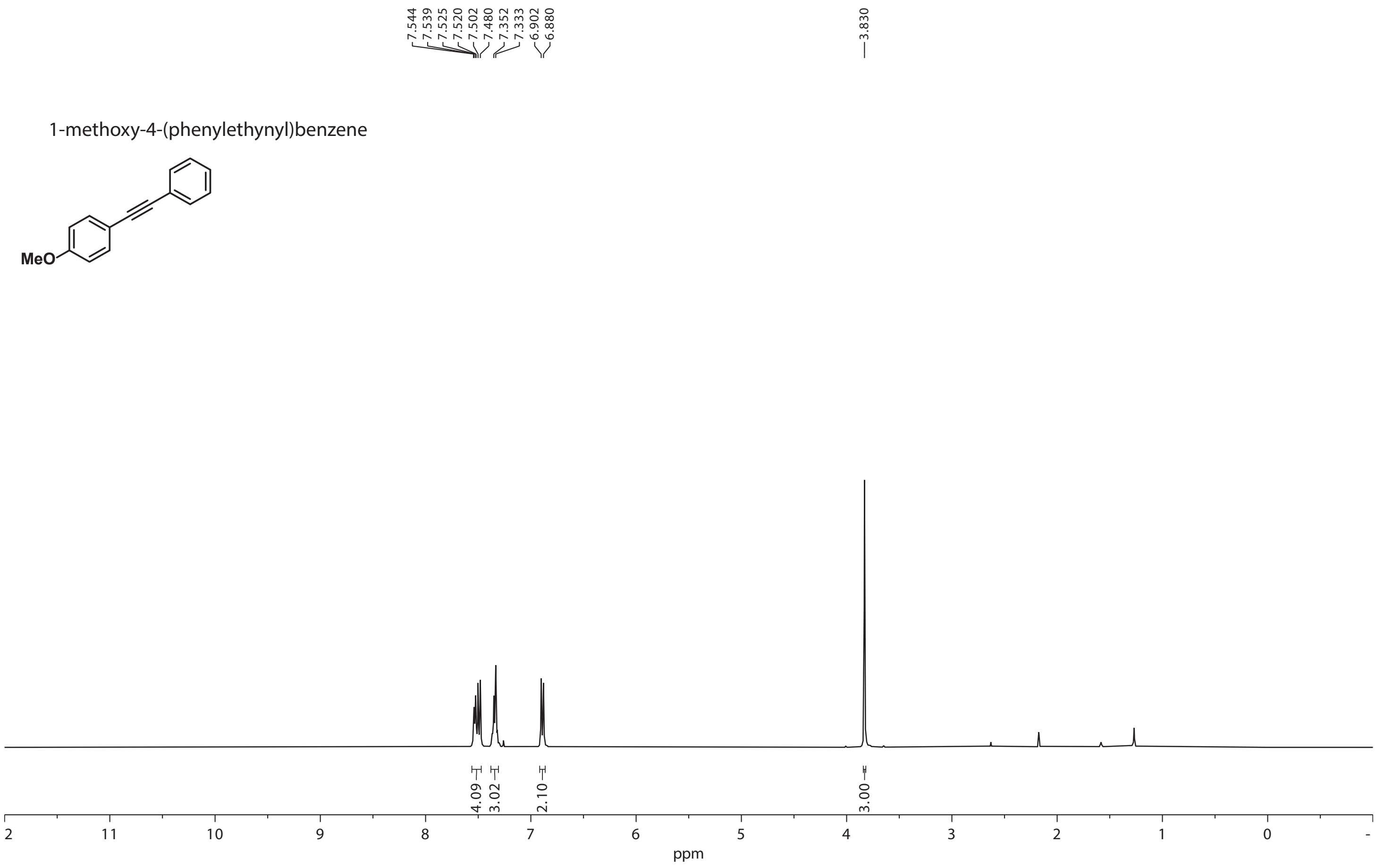

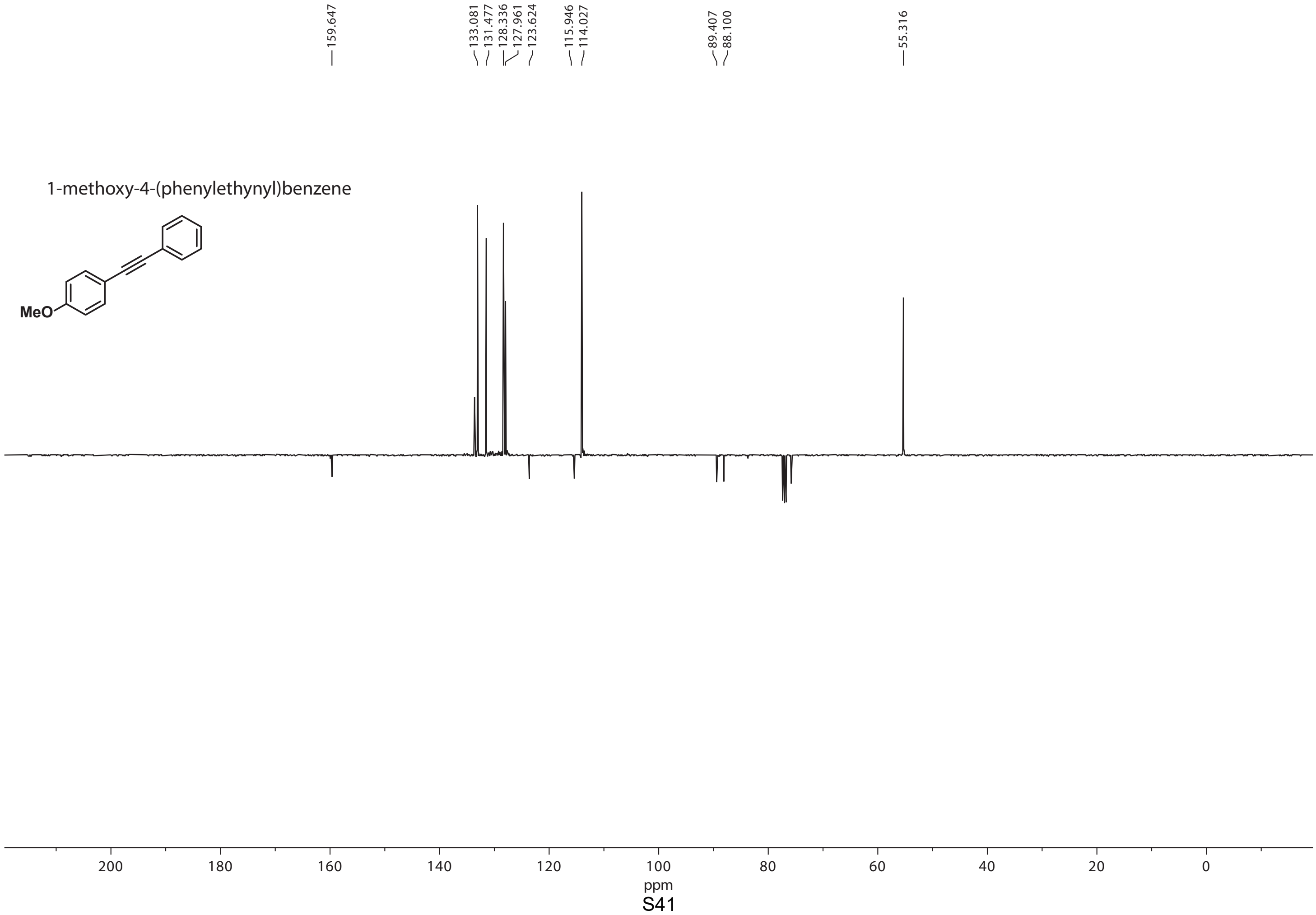

Figure S7. ¹H-NMR and ¹³C-NMR of 1-ethyl-4-(phenylethynyl)benzene (**3e**)

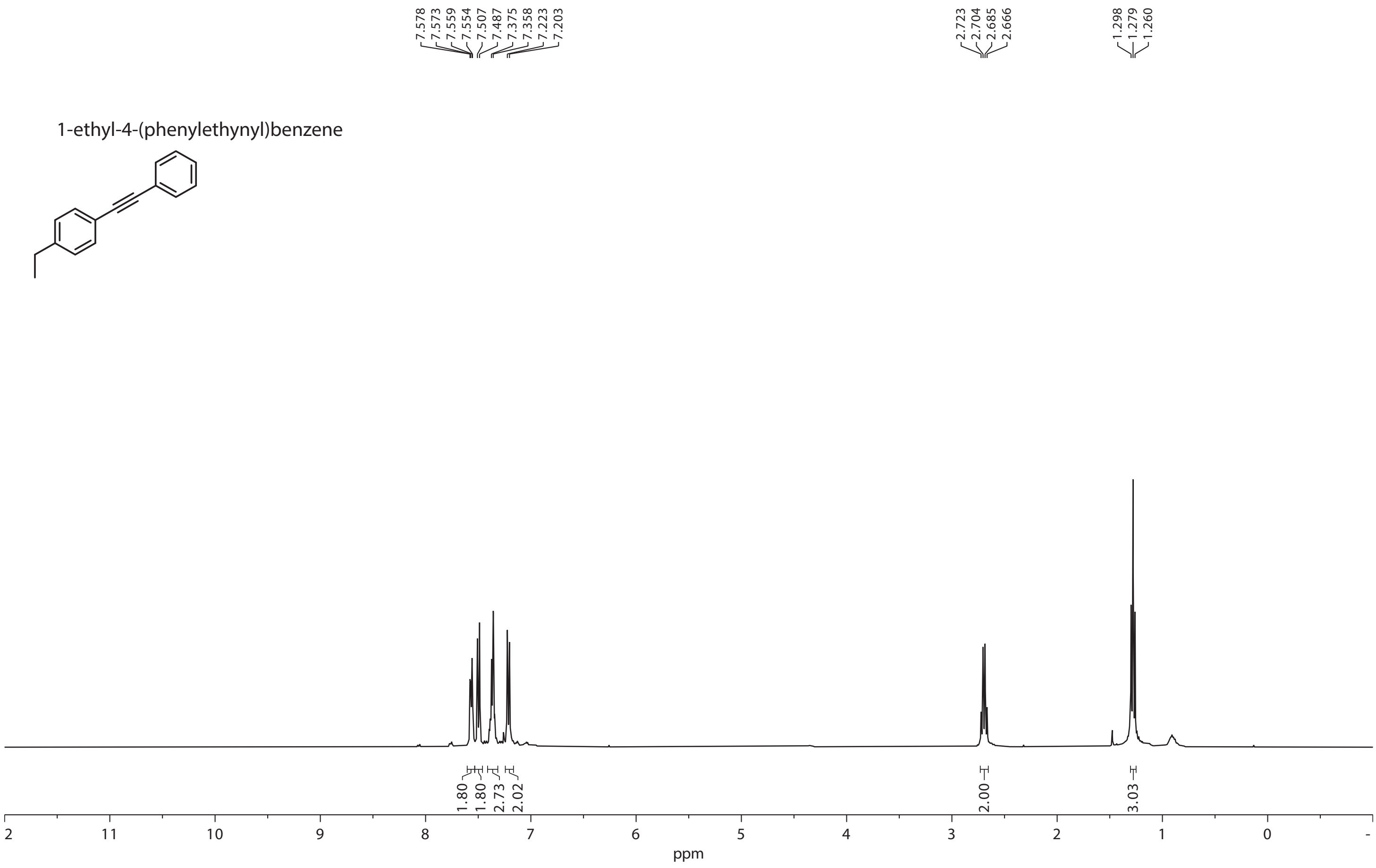

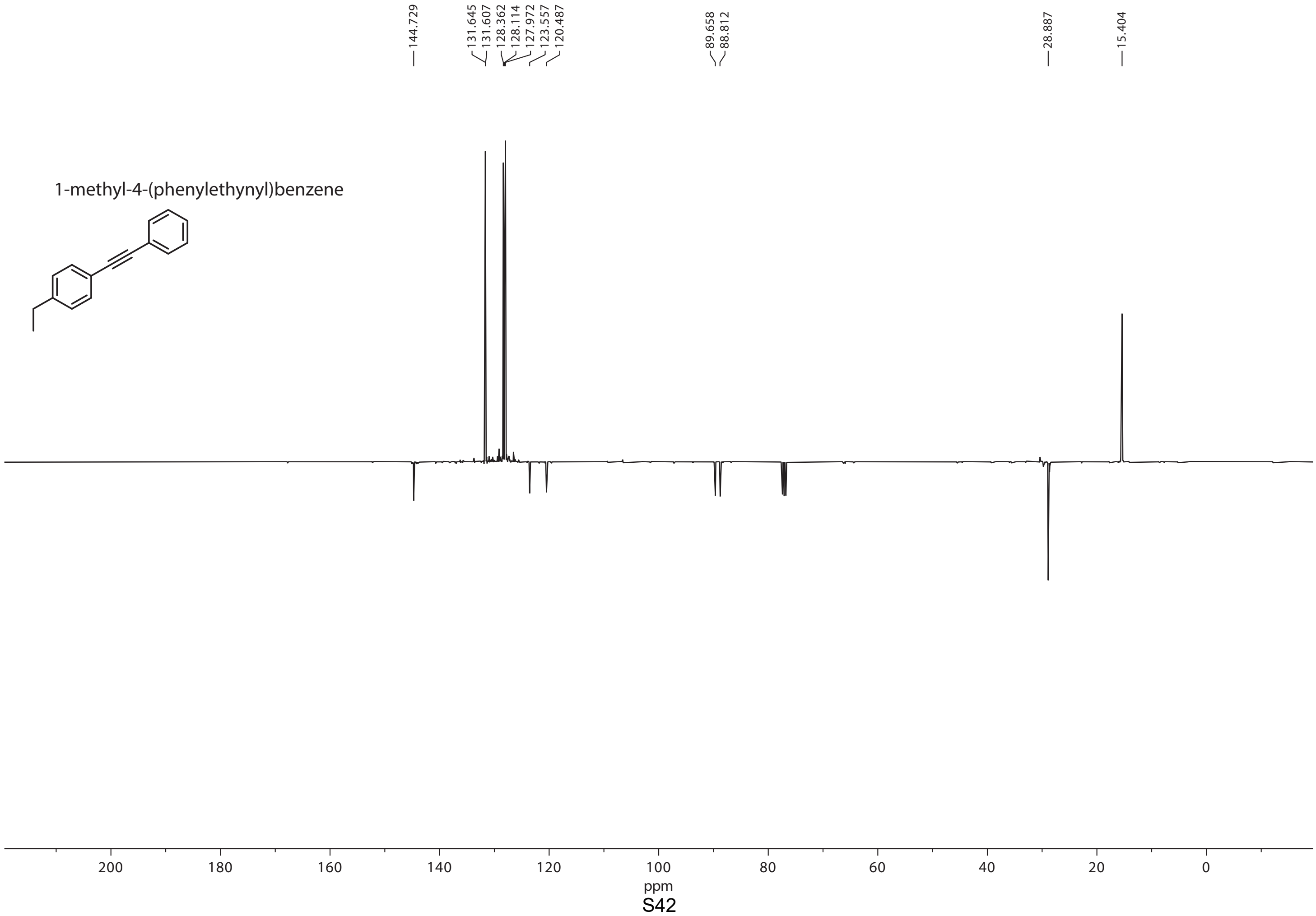

Figure S8. ^{1}H-NMR and ^{13}C-NMR of 4-(phenylethynyl)aniline (**3f**)

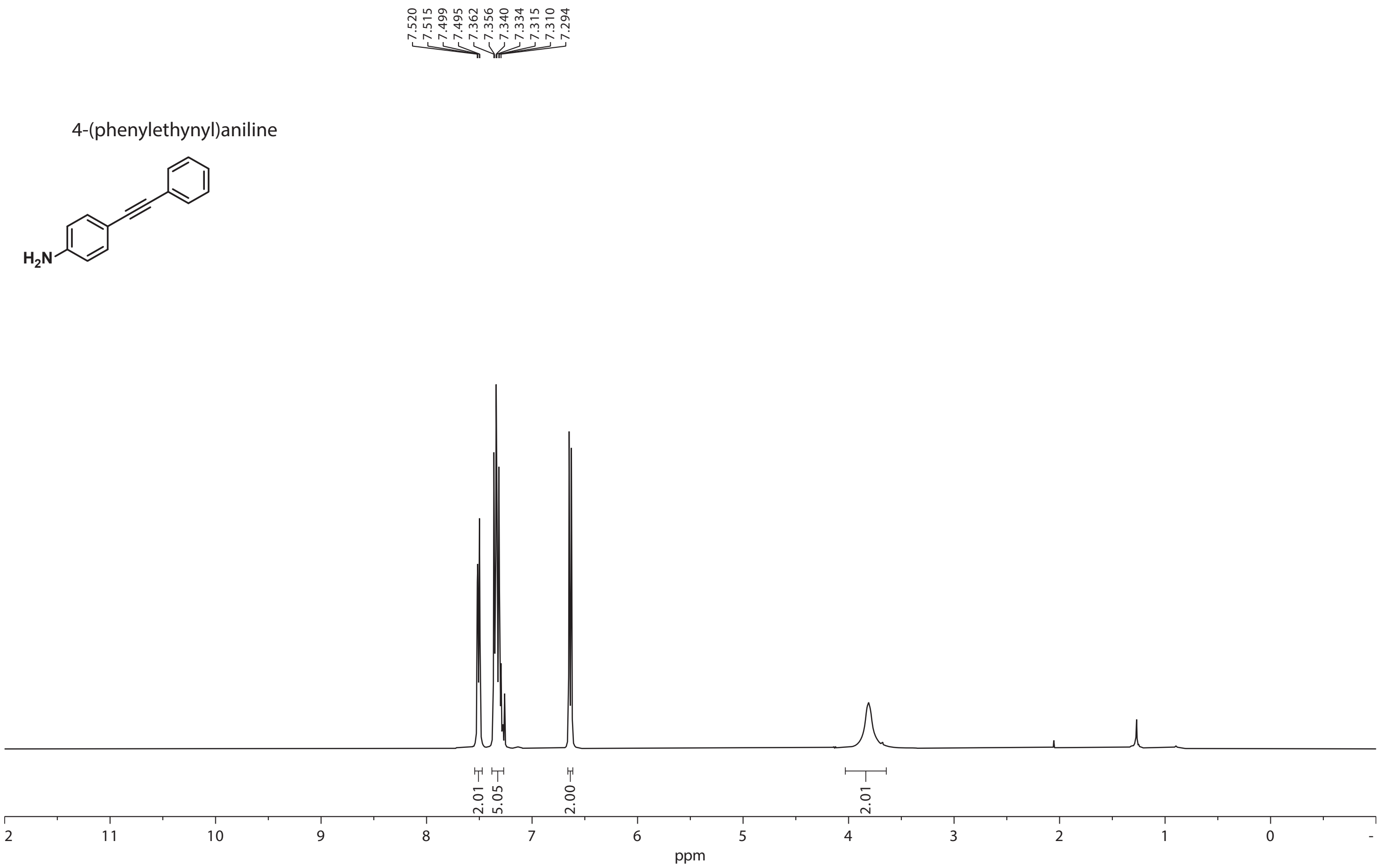

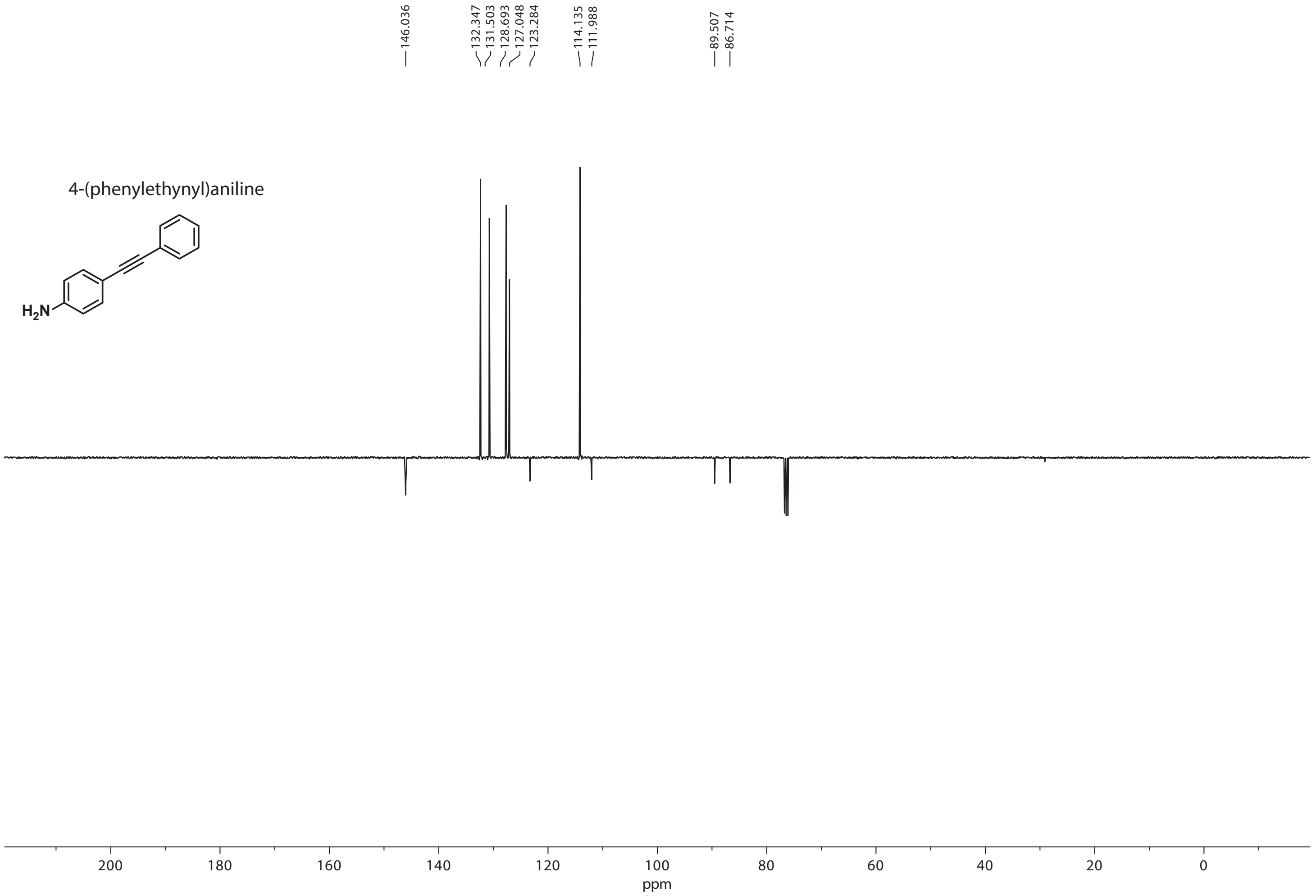

Figure S9. ^{1}H-NMR and ^{13}C-NMR of Triisopropyl(phenylethynyl)silane (**3g**)

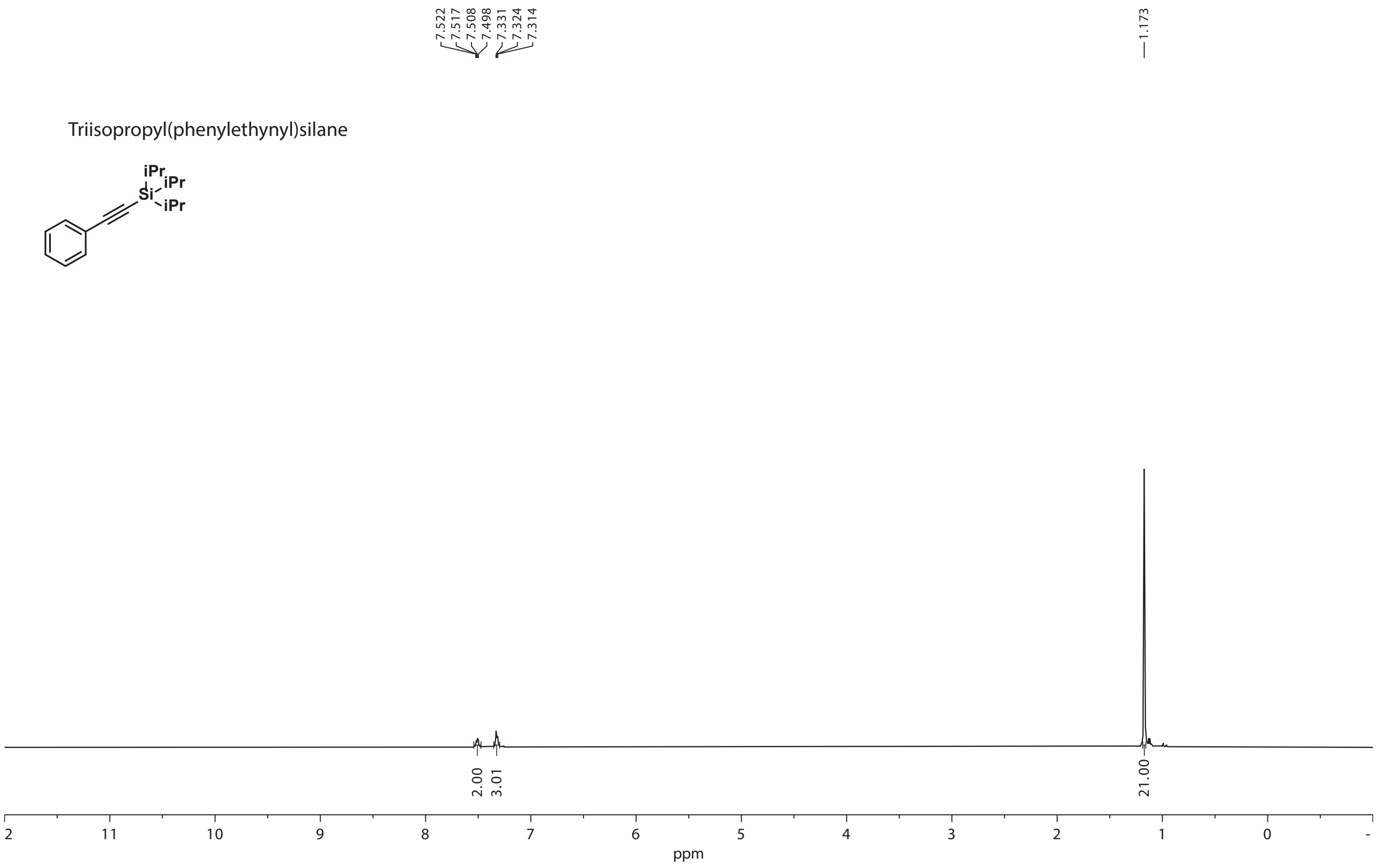

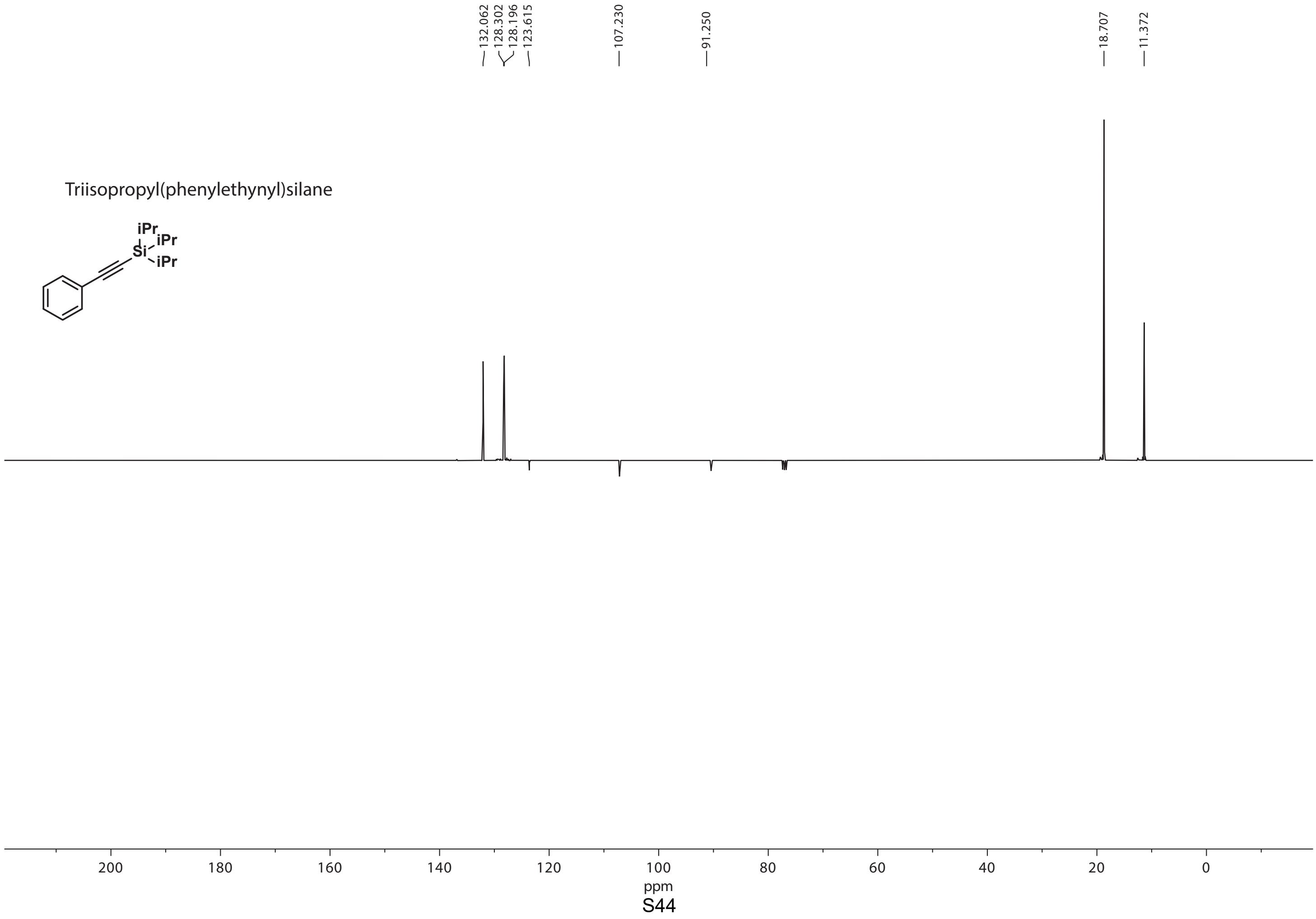

Figure S10. ^{1}H-NMR and ^{13}C-NMR of 1-chloro-4-(phenylethynyl)benzene (**3h**)

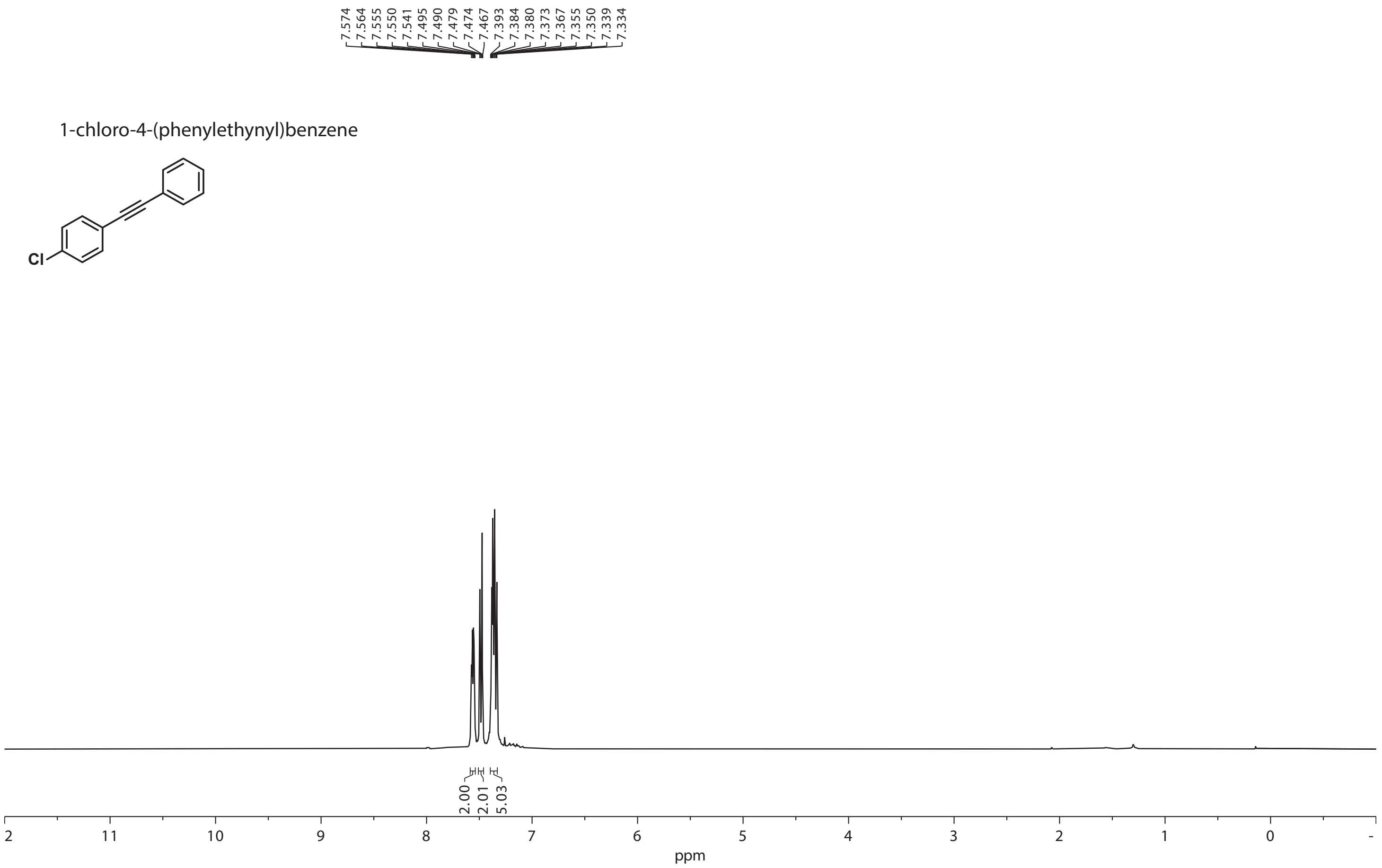

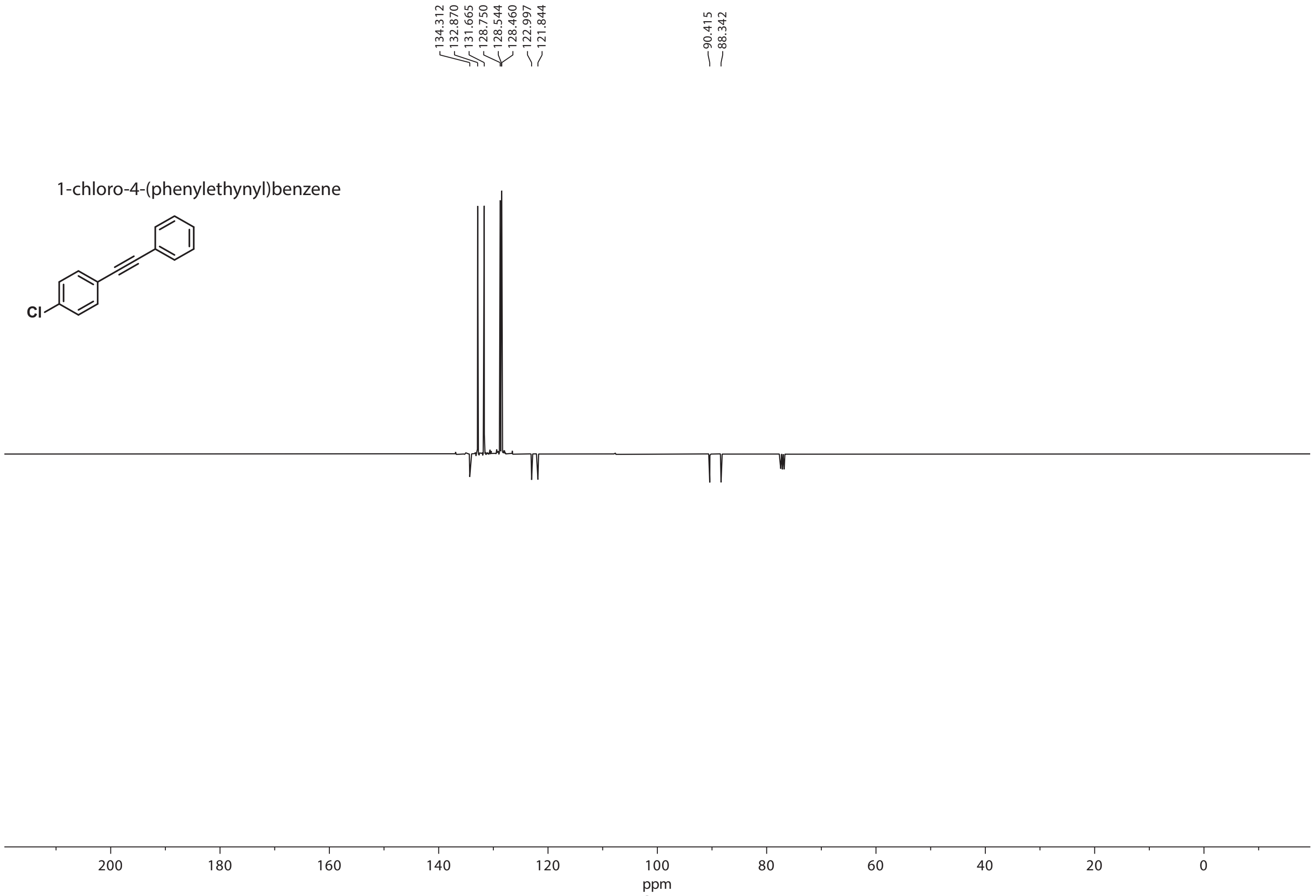

Figure S11. ^{1}H-NMR and ^{13}C-NMR of 1-chloro-3-(phenylethynyl)benzene (**3i**)

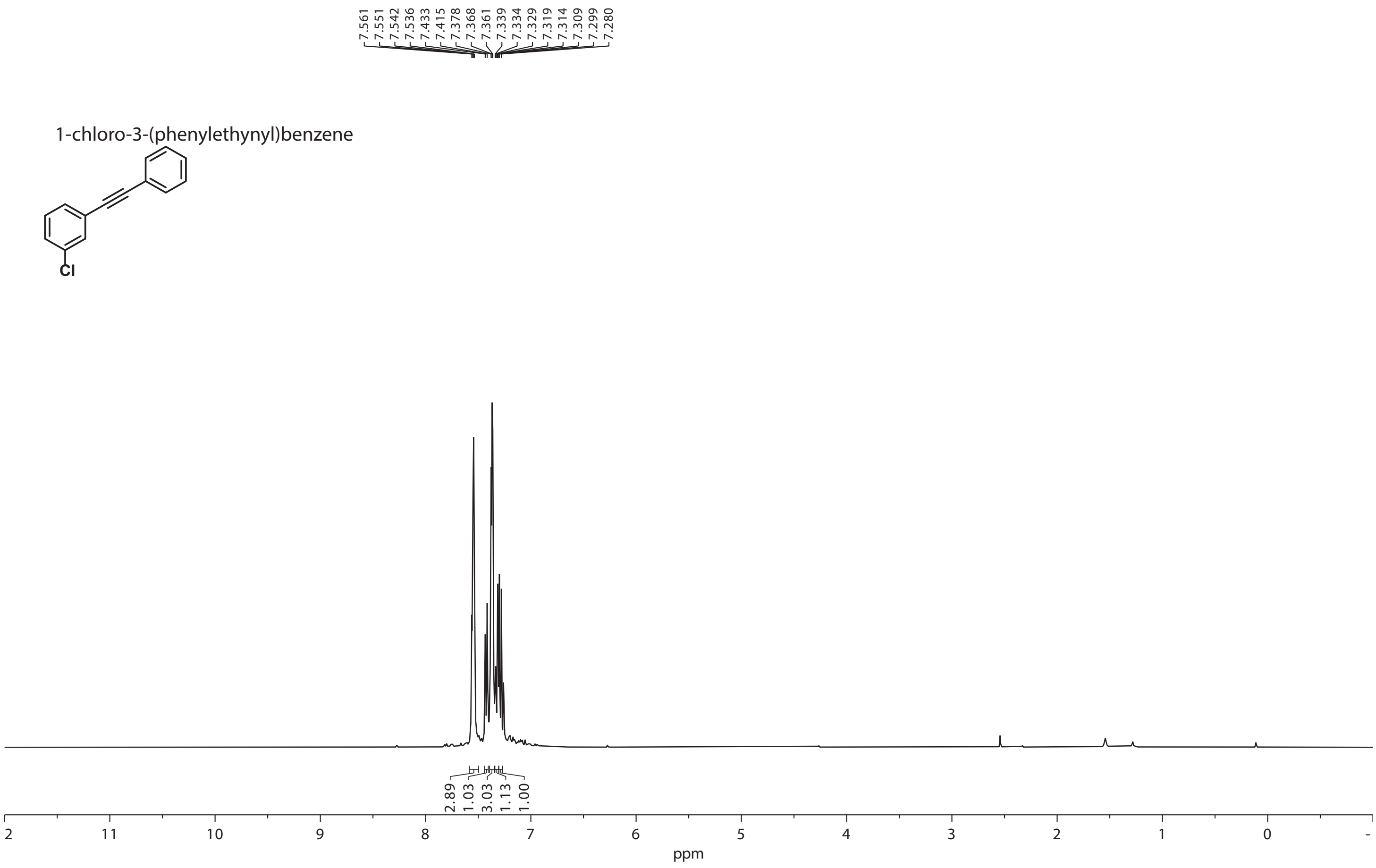

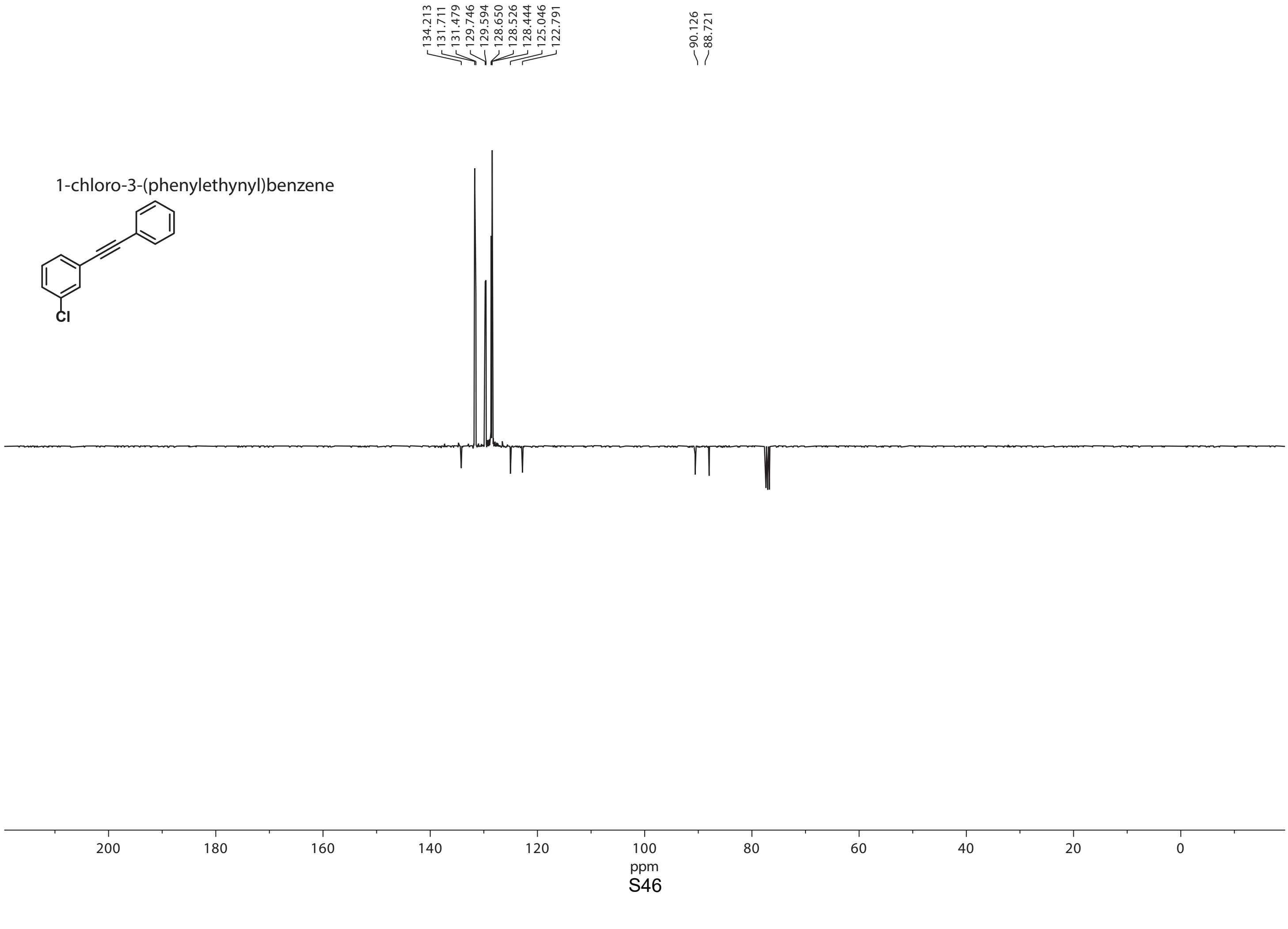

Figure S12. ^{1}H-NMR and ^{13}C-NMR of 1-(phenylethynyl)-4-(tri luoromethyl)benzene (**3j)**

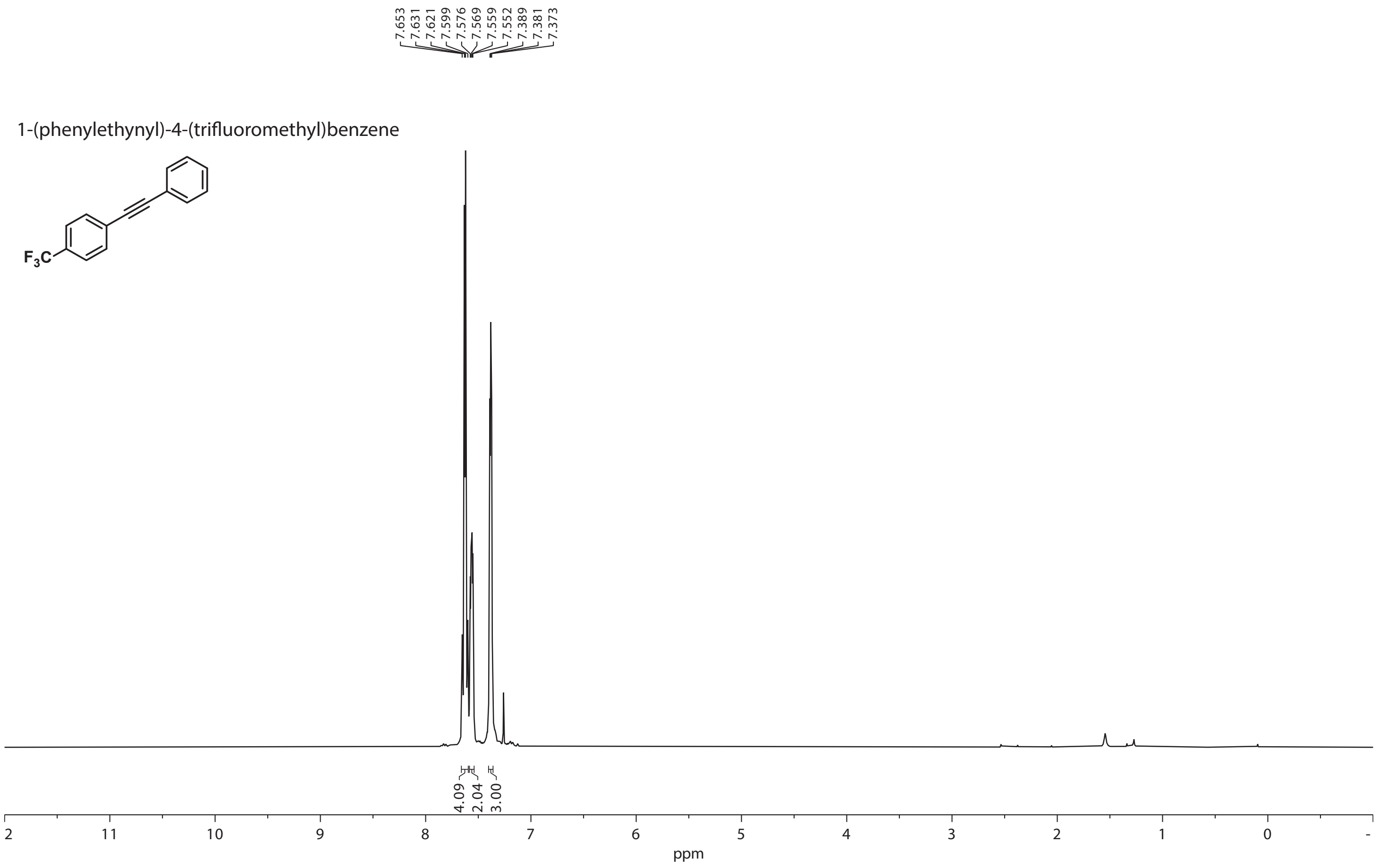

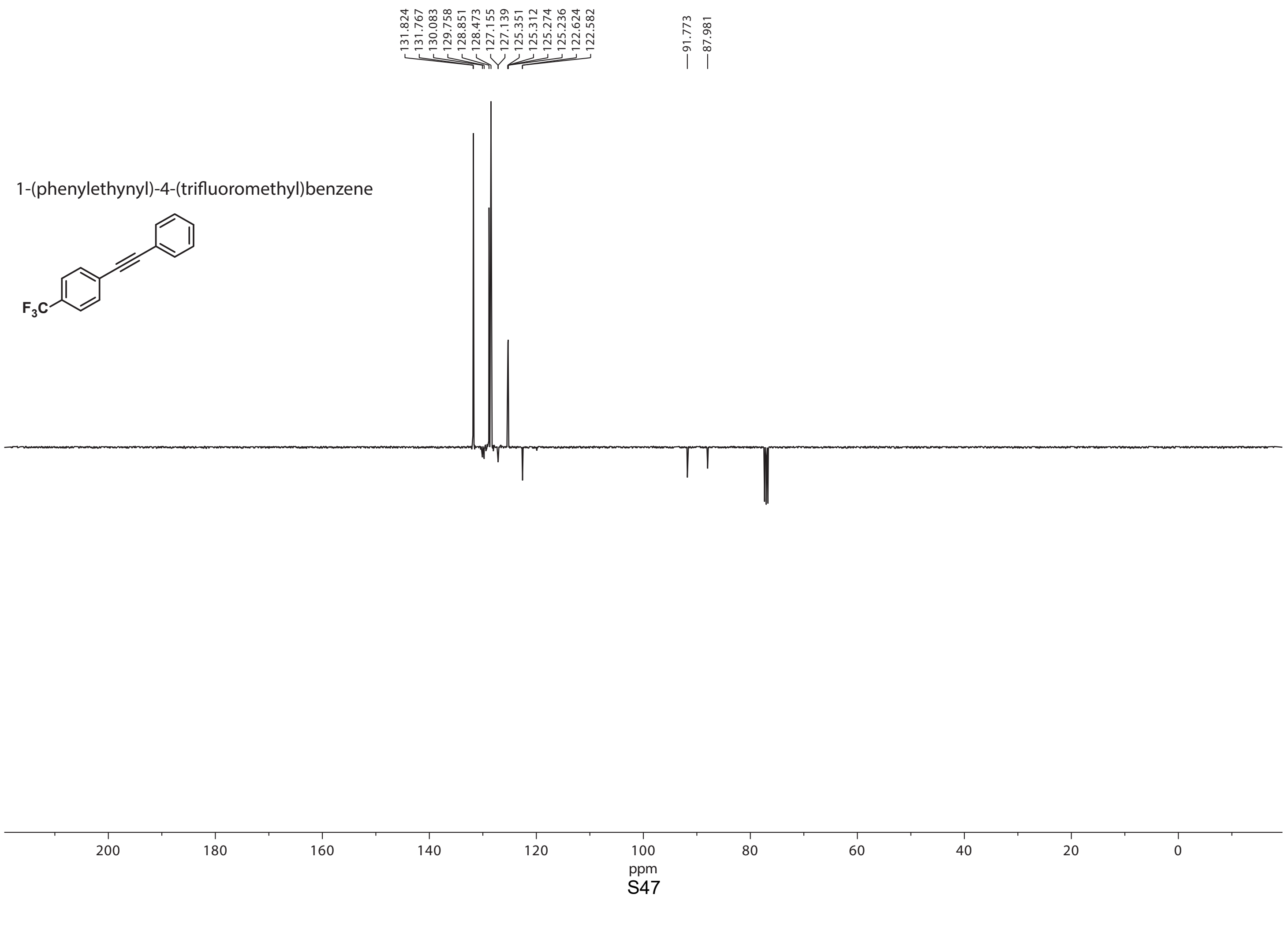

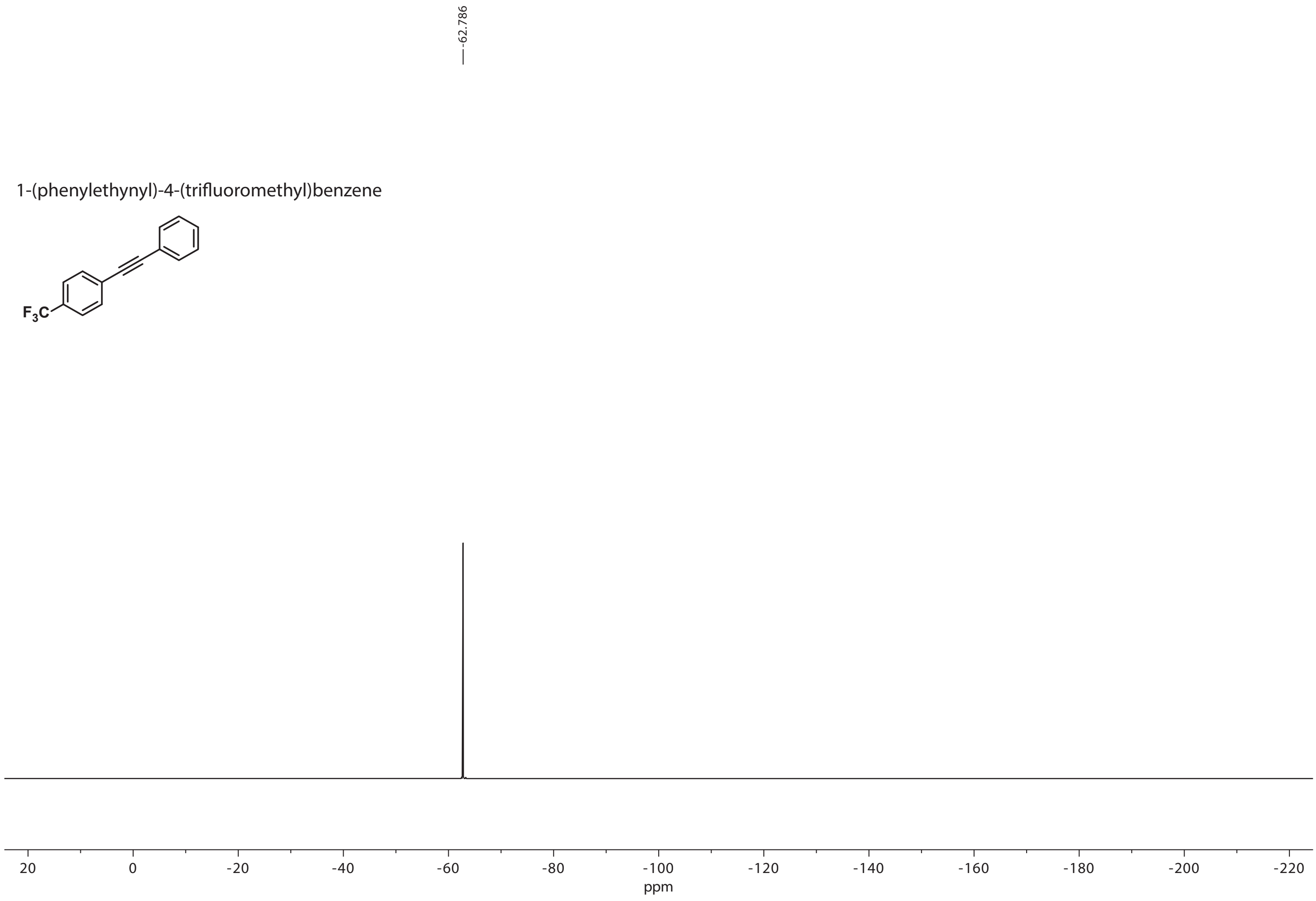

Figure S13. ^{1}H-NMR and ^{13}C-NMR of 1-methyl-2-(phenylethynyl)benzene (**3k**)

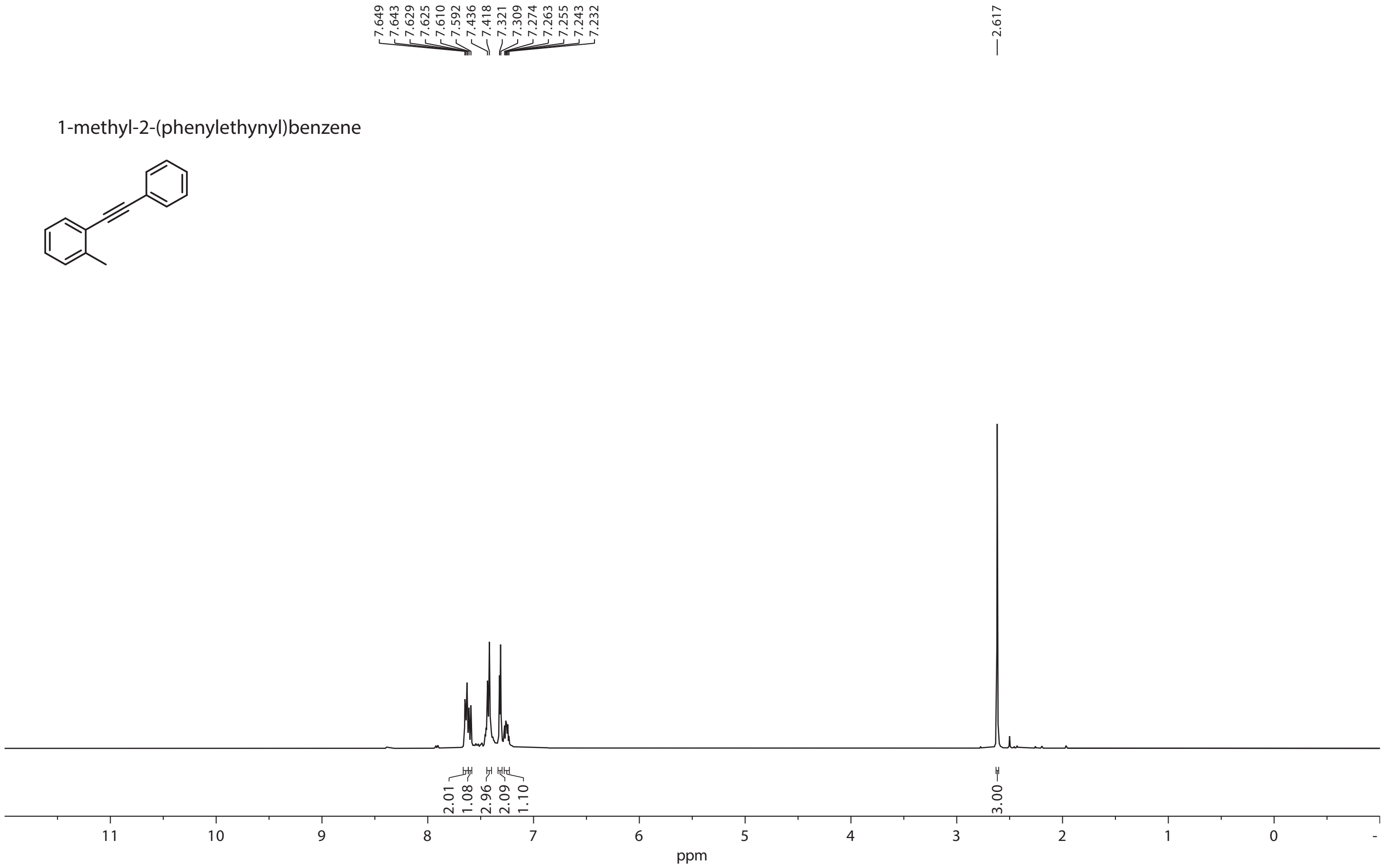

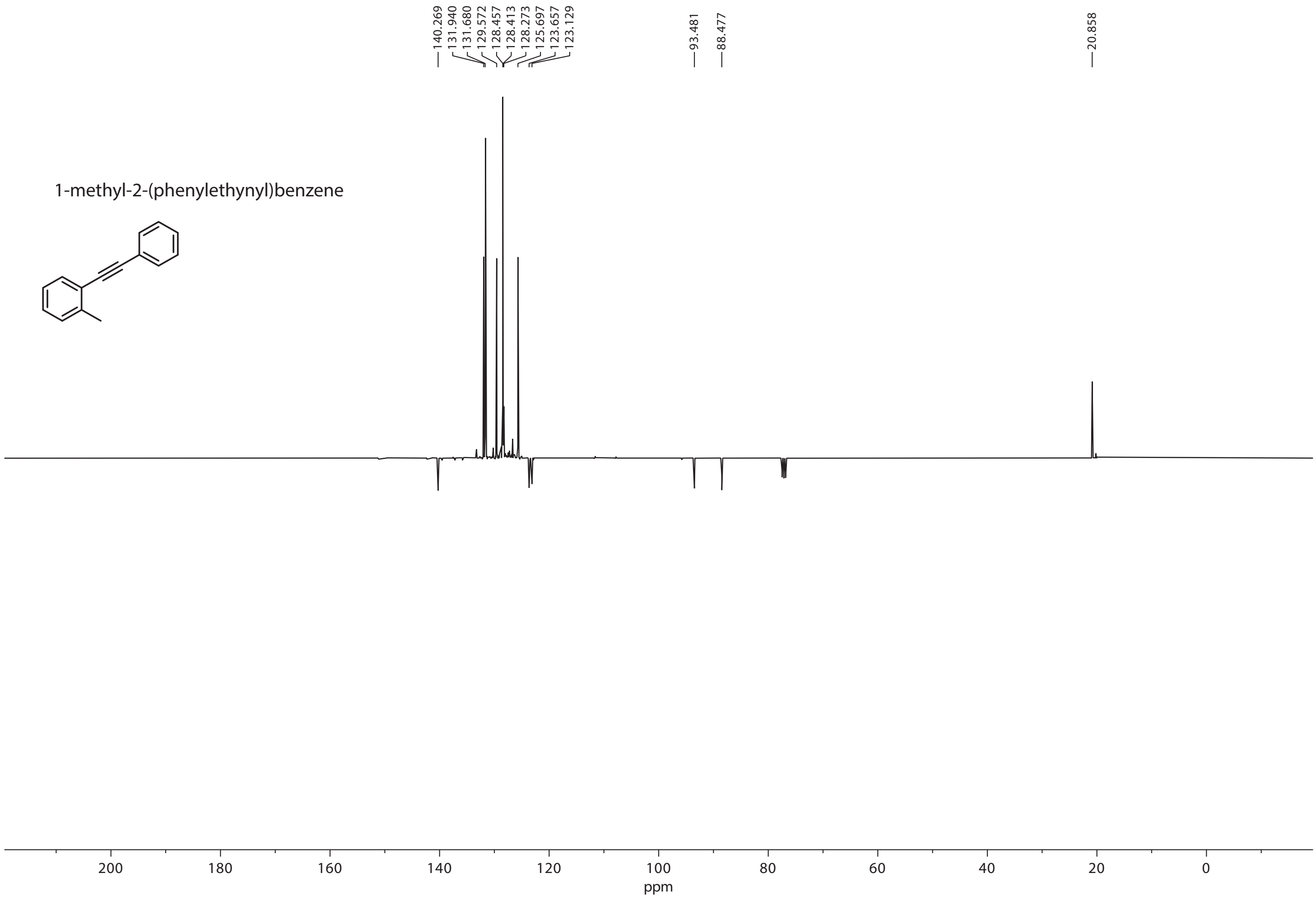

Figure S14. ¹H-NMR and ¹³C-NMR of 1-(tert-butyl)-4-(phenylethynyl)benzene (**3l**)

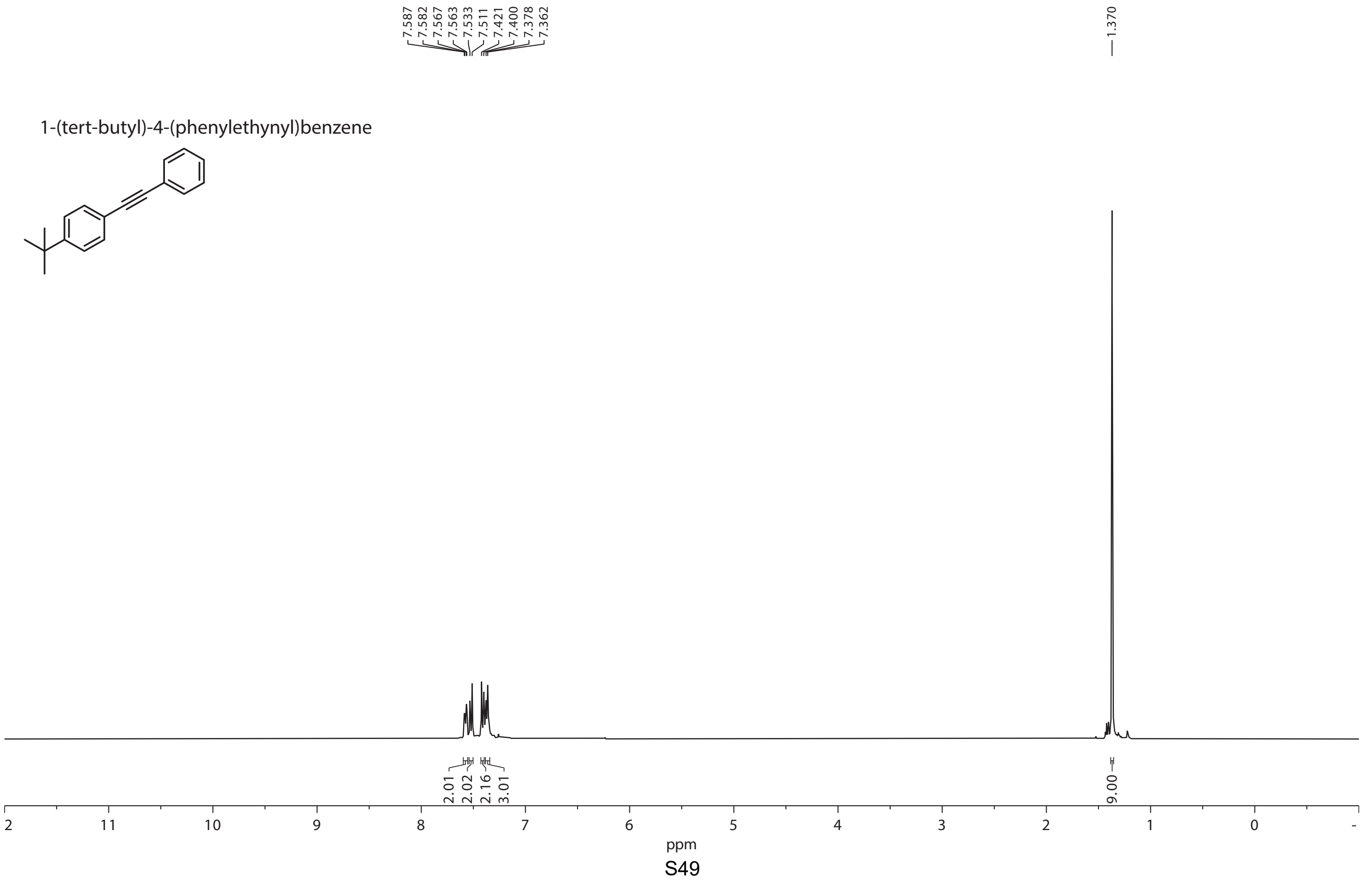

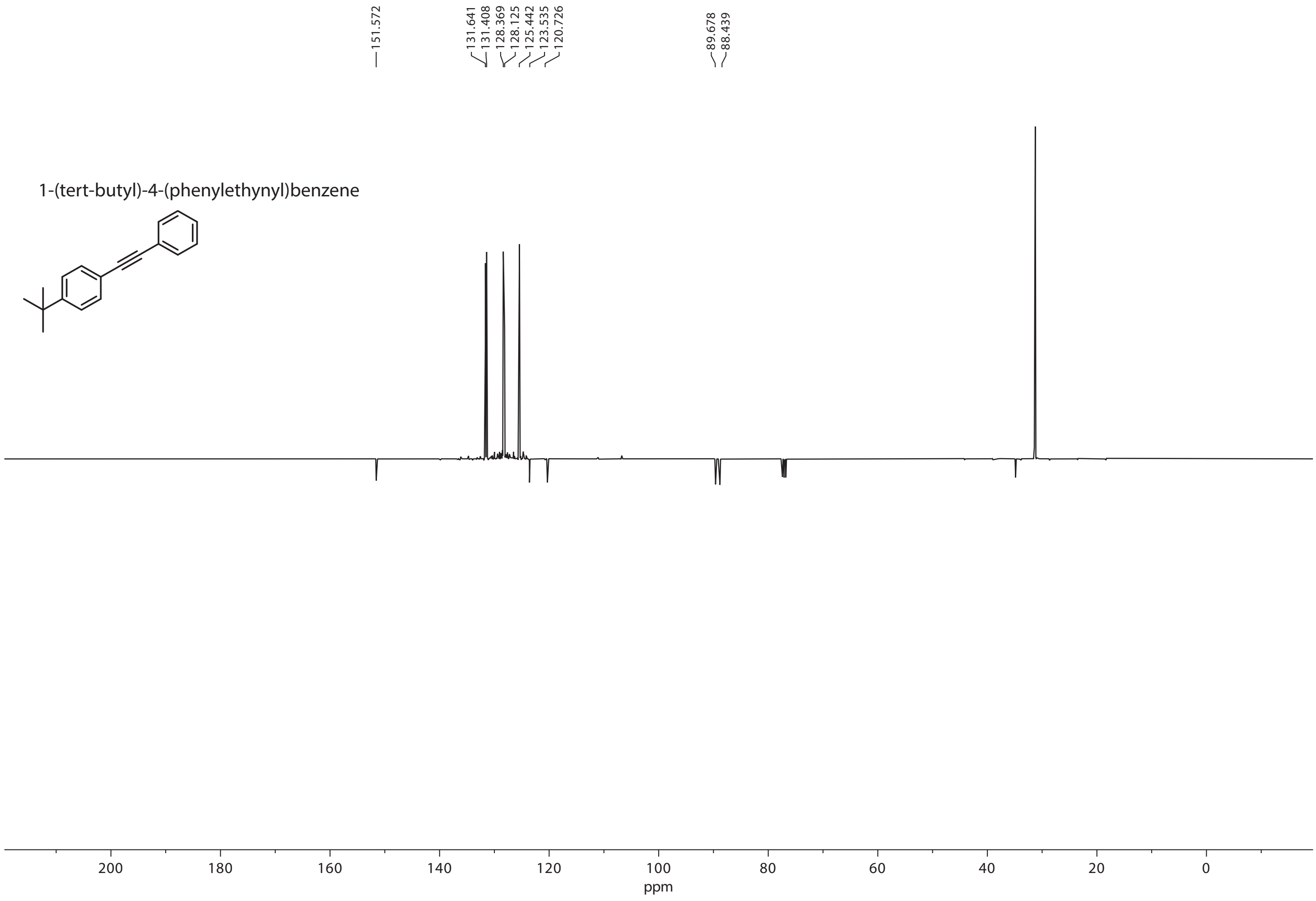

Figure S15. ^{1}H-NMR and ^{13}C-NMR of 1-methoxy-3-(phenylethynyl)benzene (**3m)**

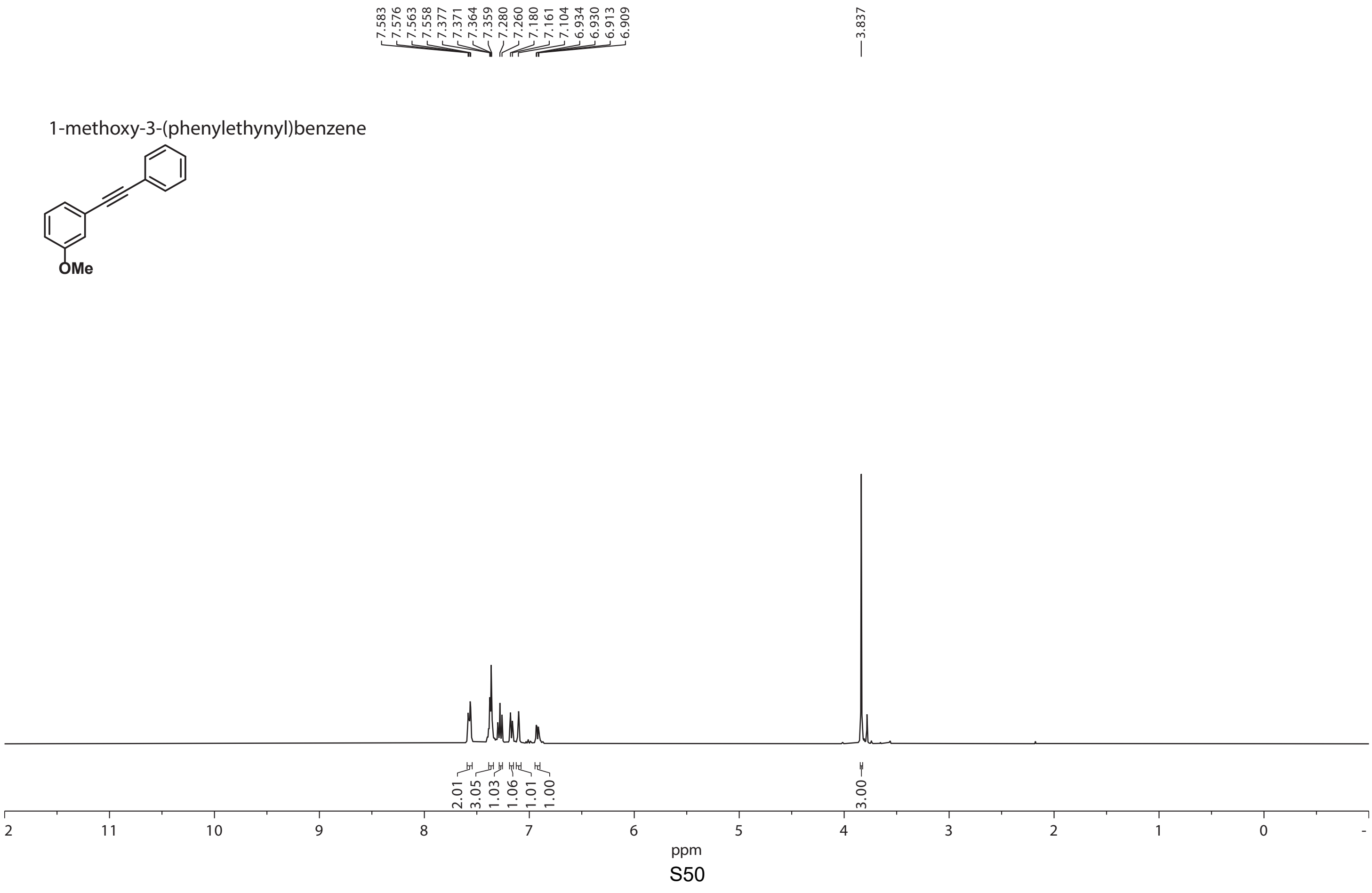

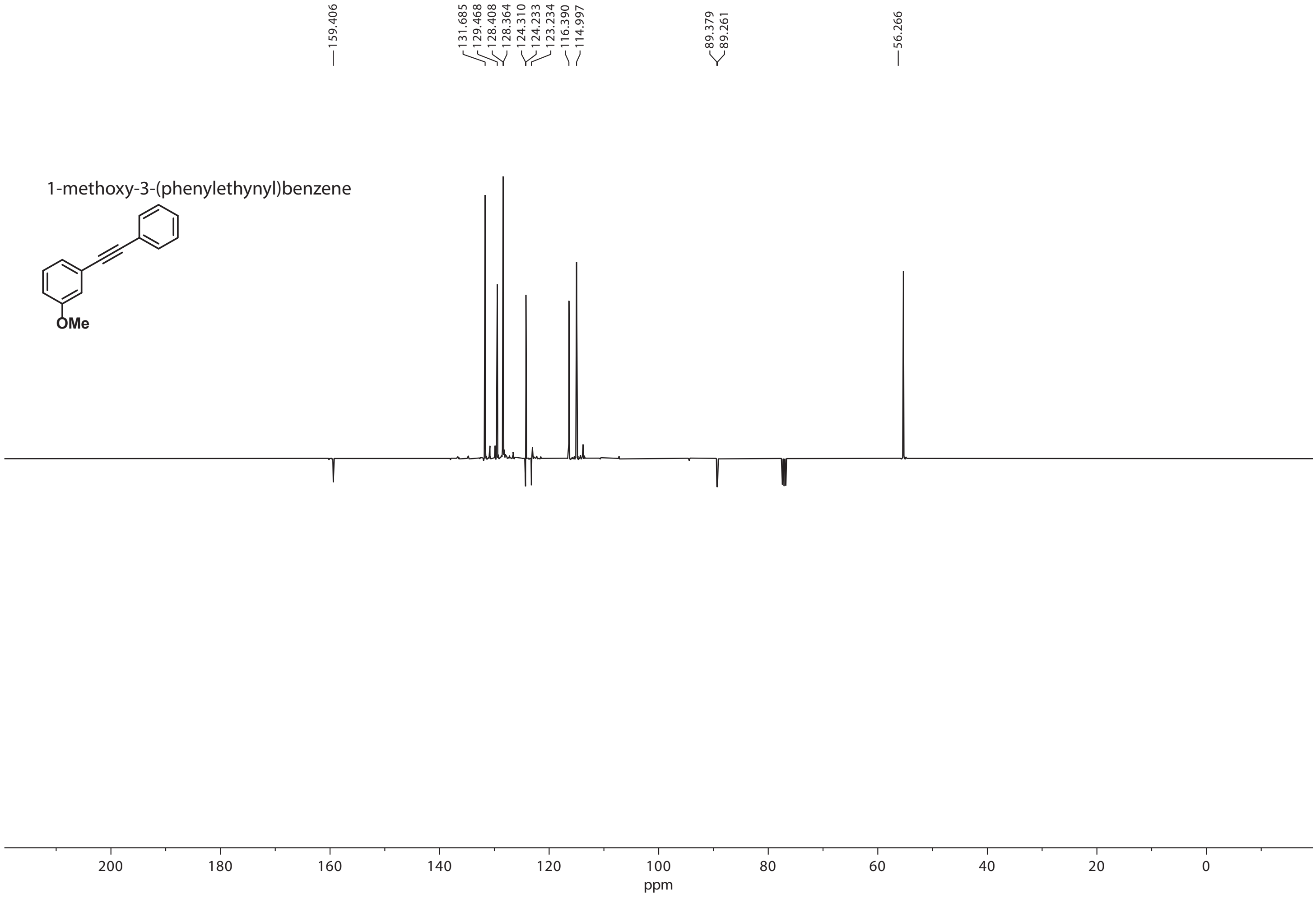

Figure S16. ¹H-NMR and ¹³C-NMR of 1-(phenylethynyl)naphthalene (**3n**)

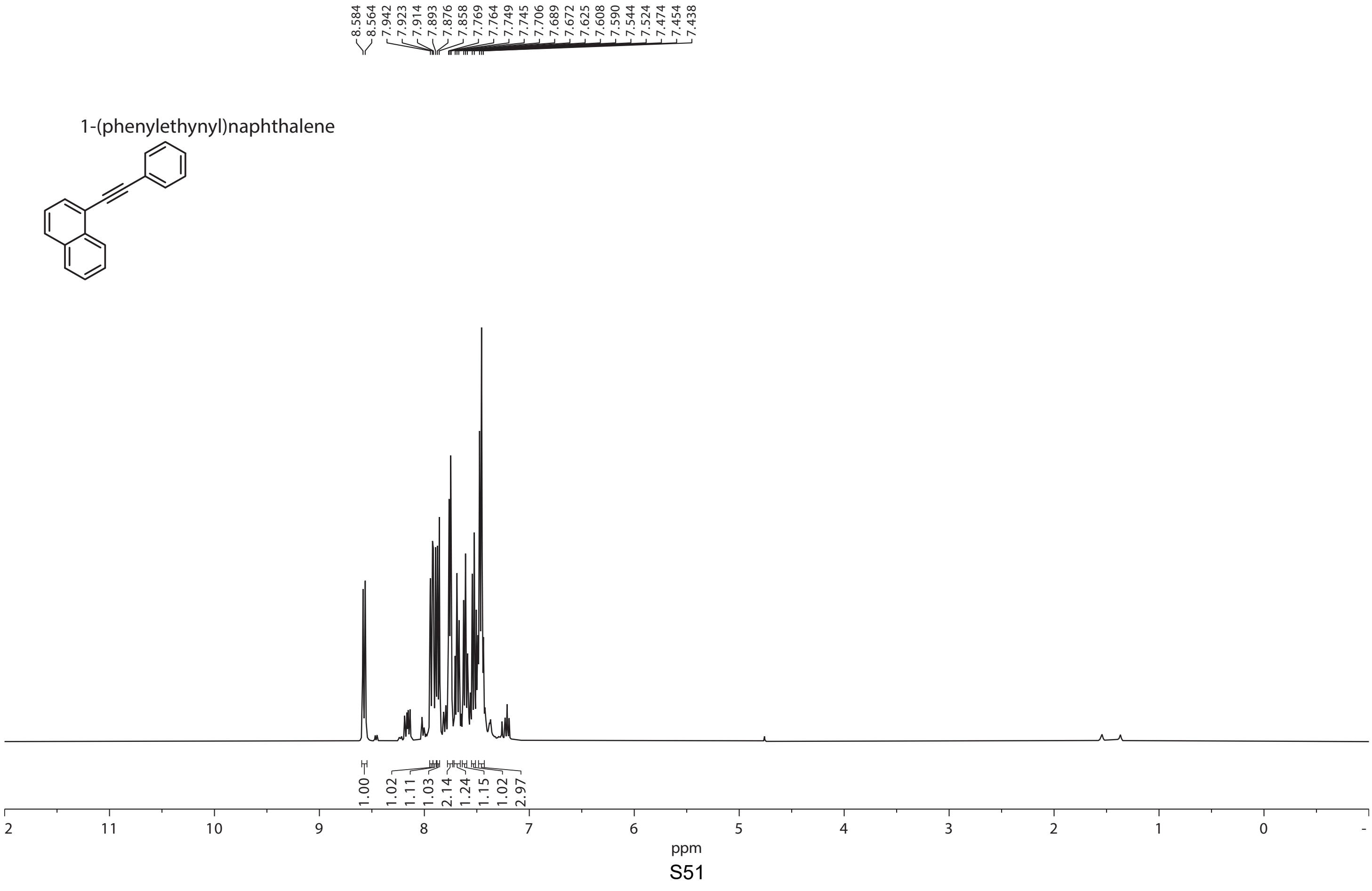

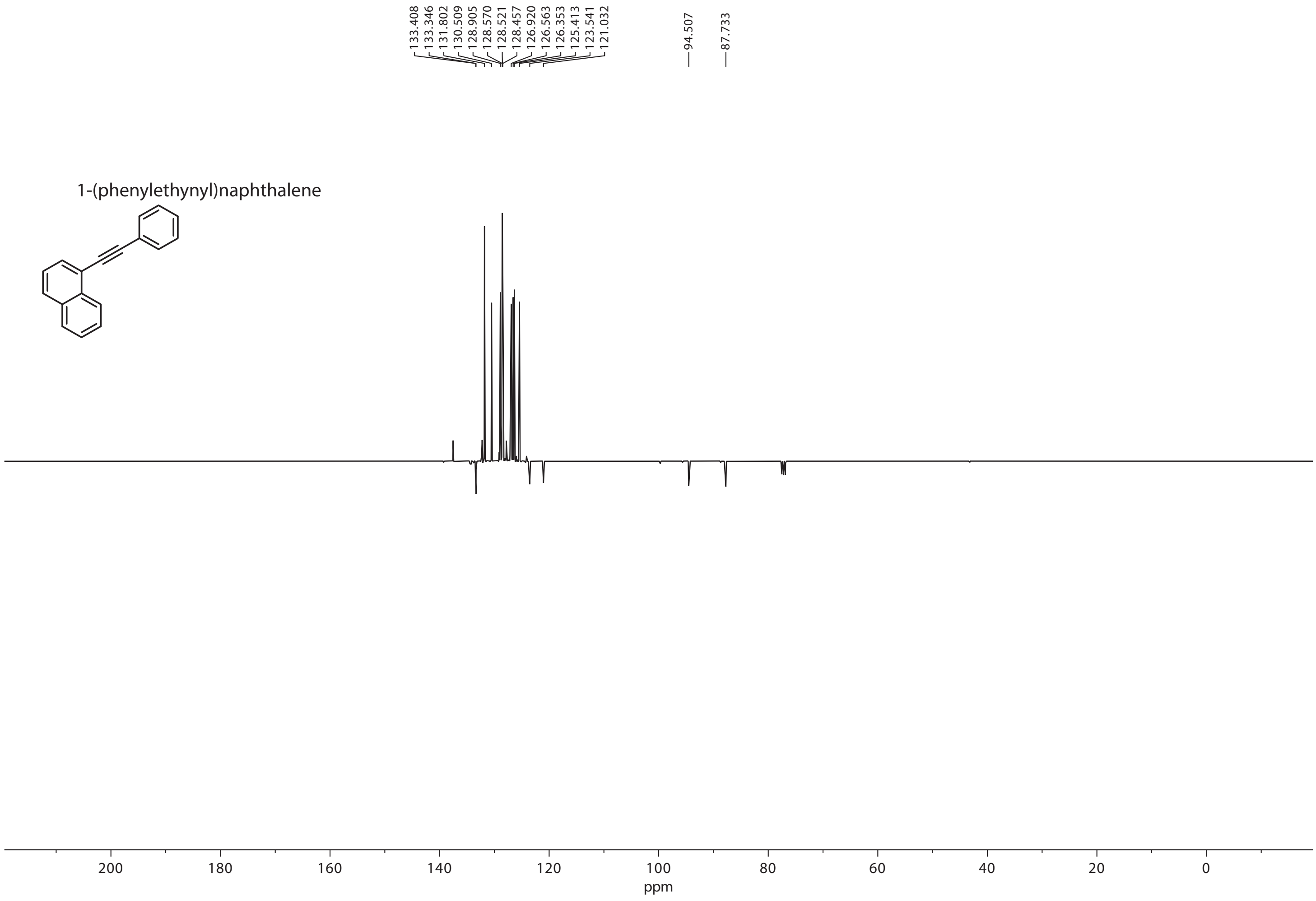

Figure S17. ^{1}H-NMR and ^{13}C-NMR of Ethyl 4-(phenylethynyl)benzoate (**3o**)

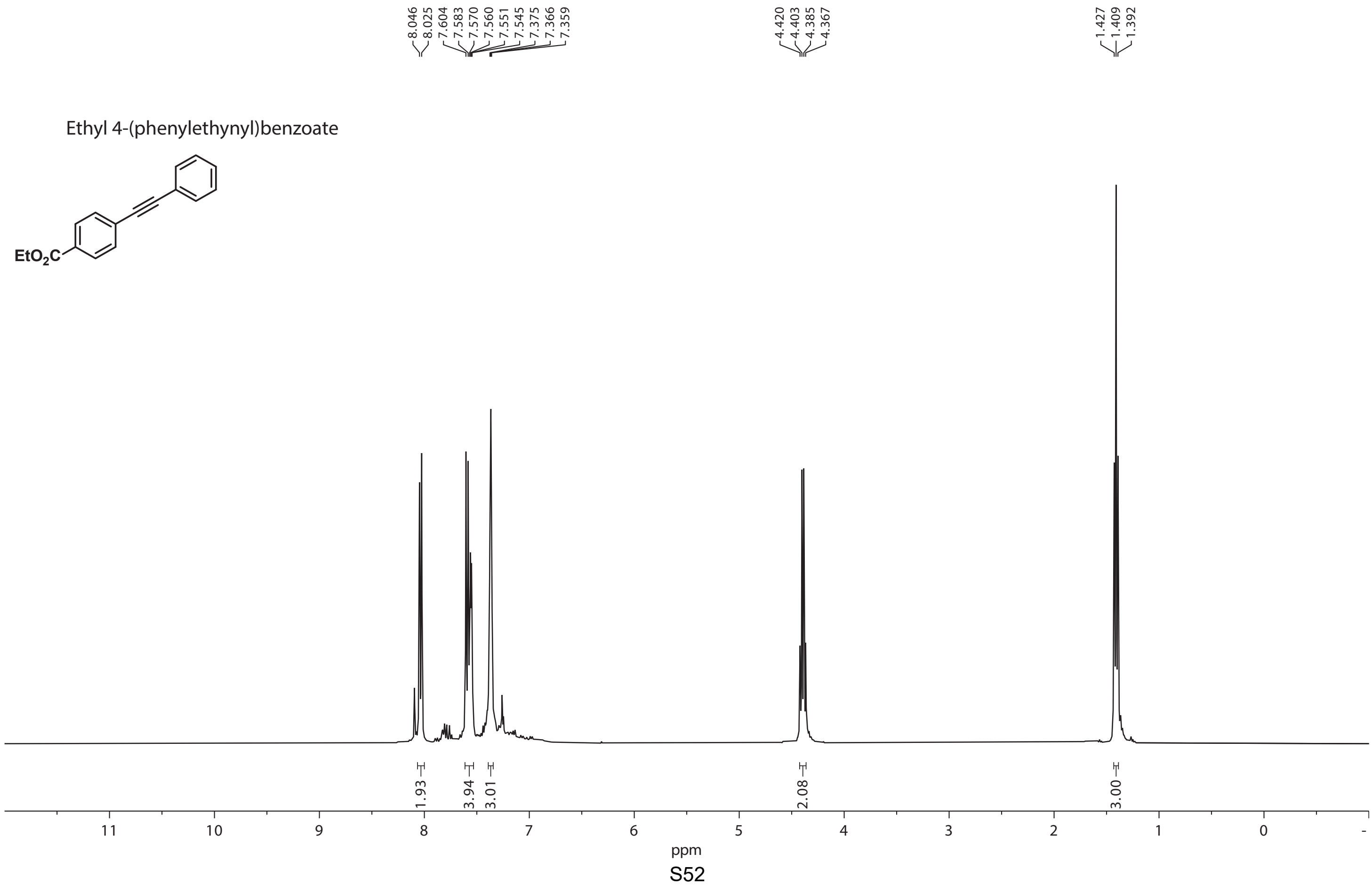

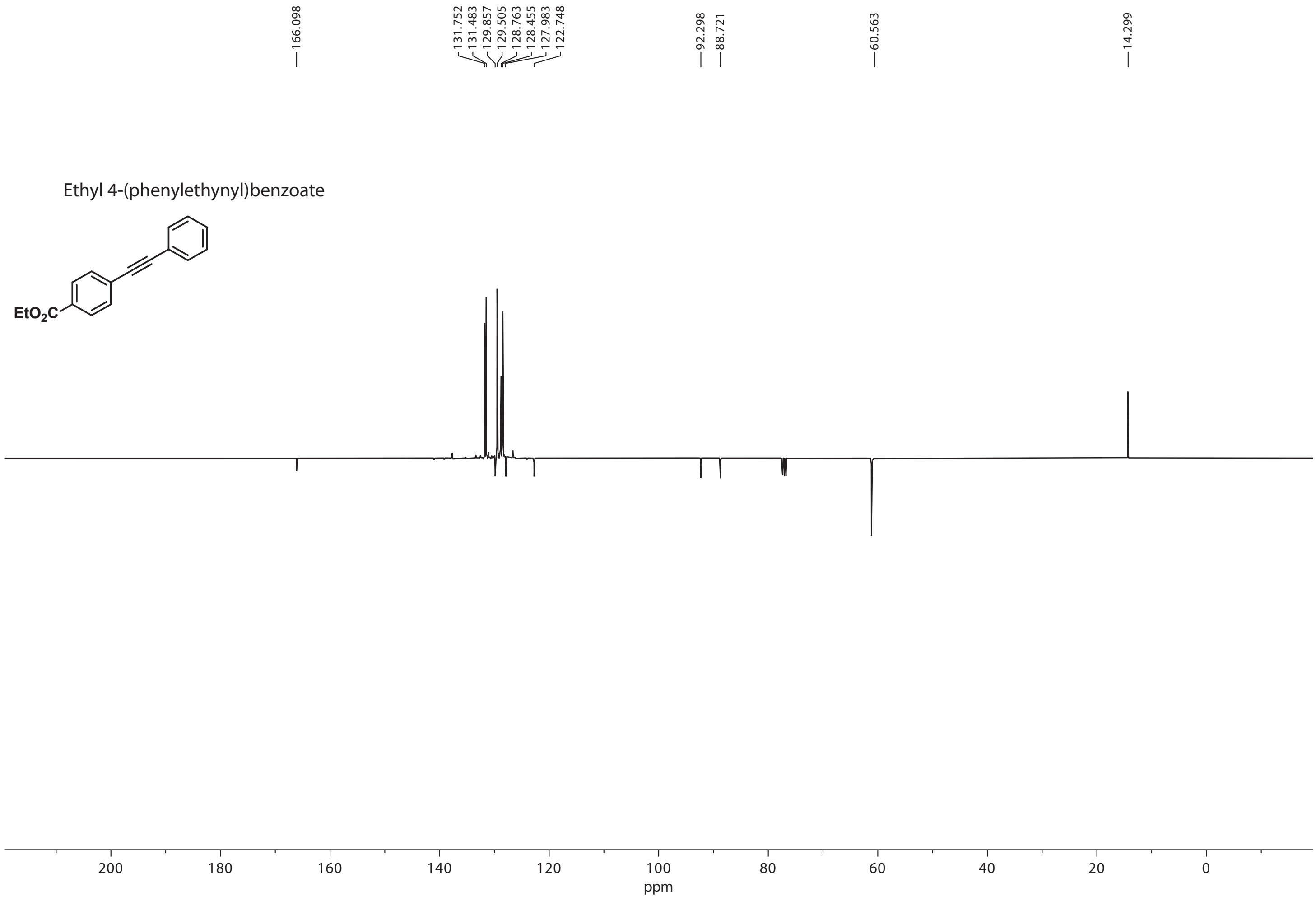

Figure S18. ^{1}H-NMR and ^{13}C-NMR of 1-ethyl-4-(p-tolylethynyl)benzene (**3p**)

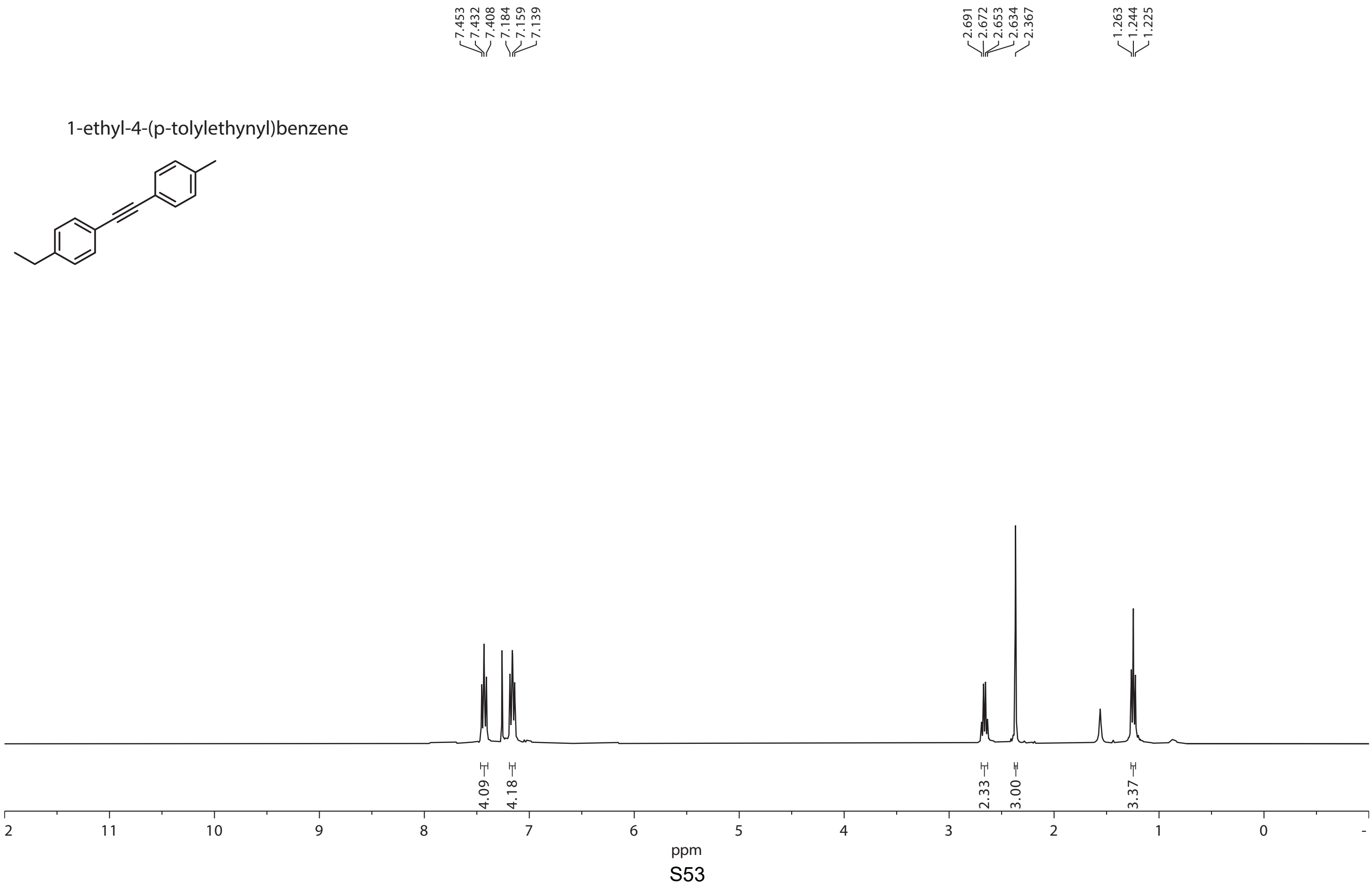

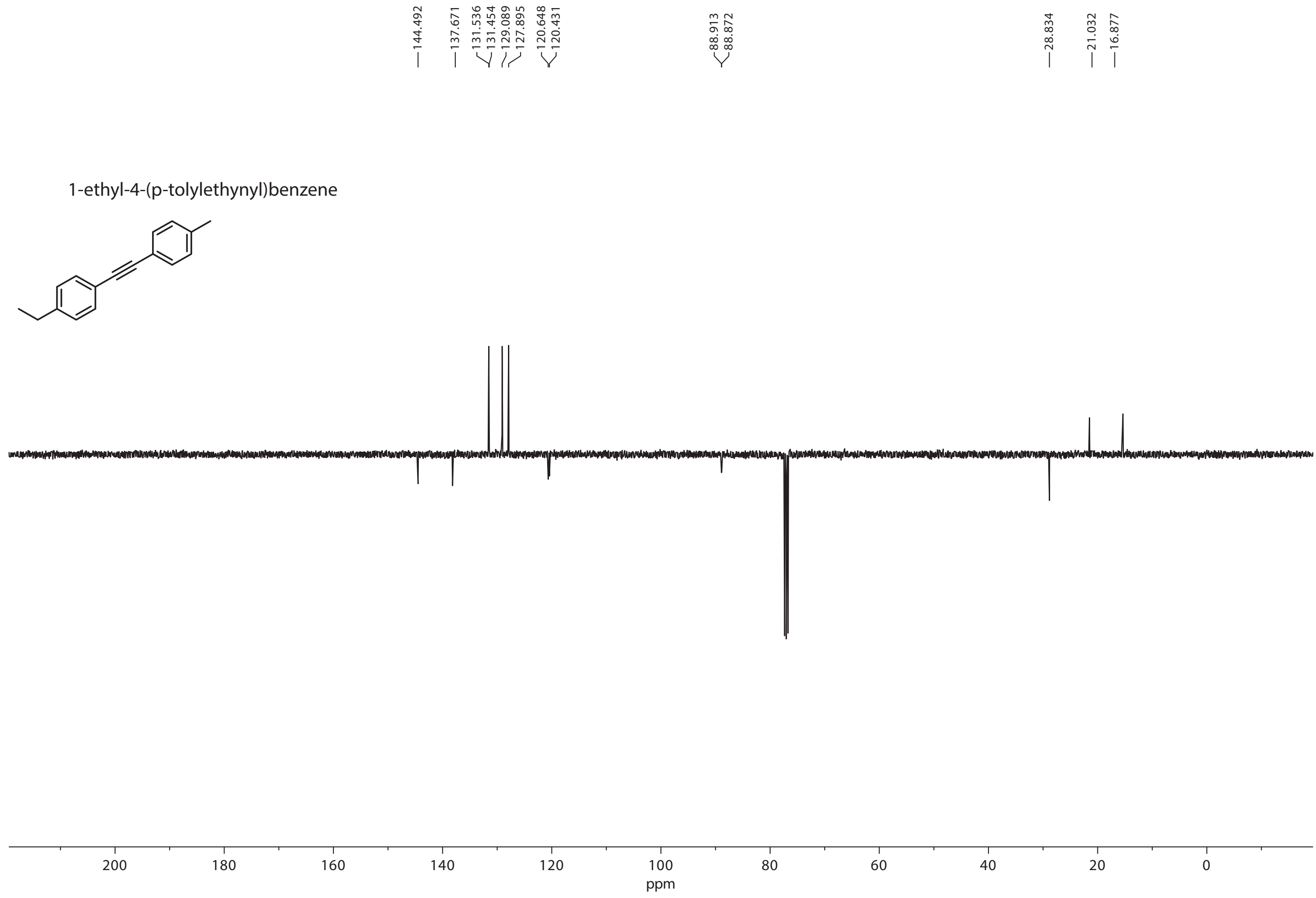

Figure S19. ^{1}H-NMR and ^{13}C-NMR of 1-bromo-4-((4-chlorophenyl)ethynyl)benzene (**3q)**

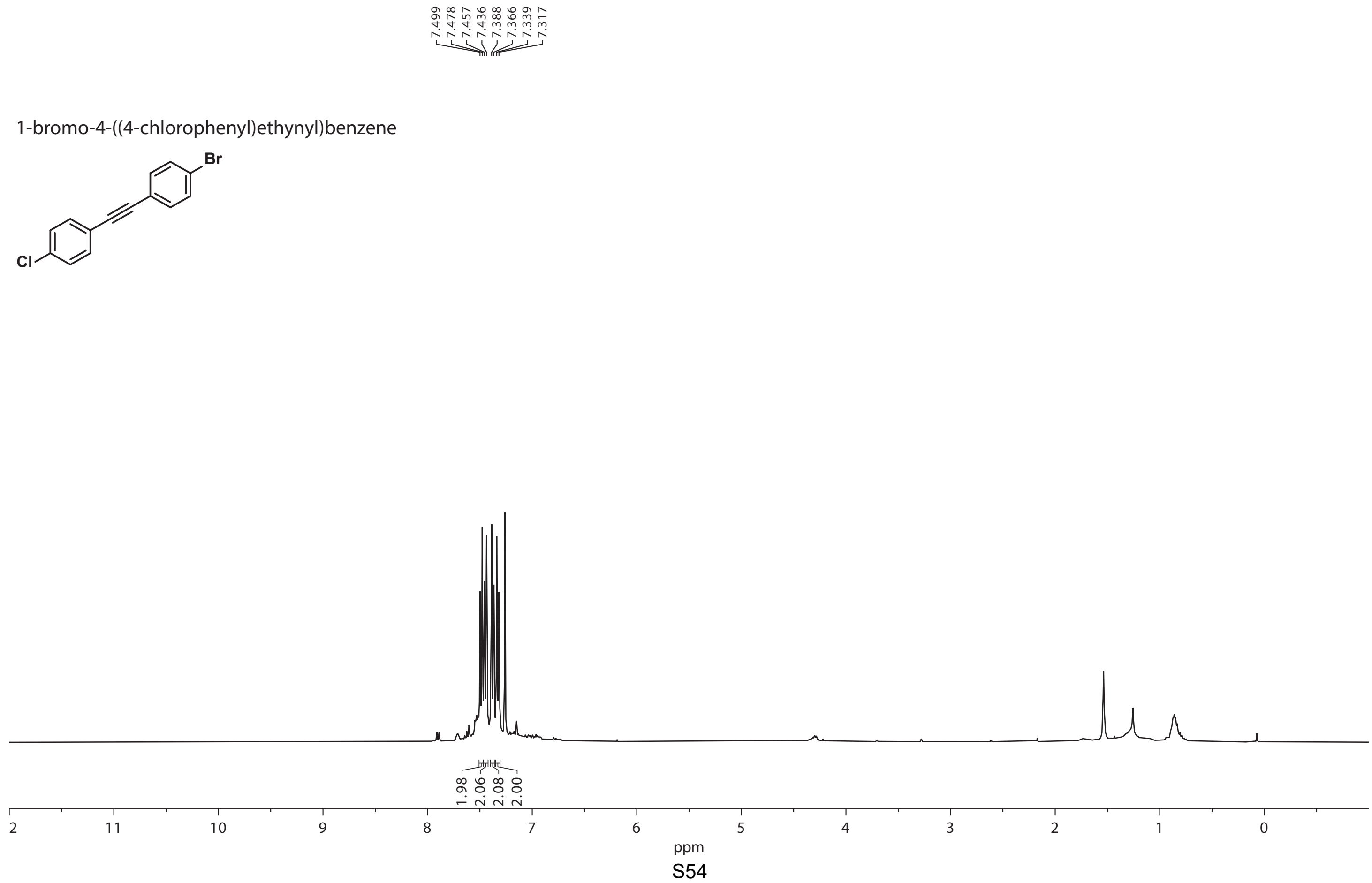

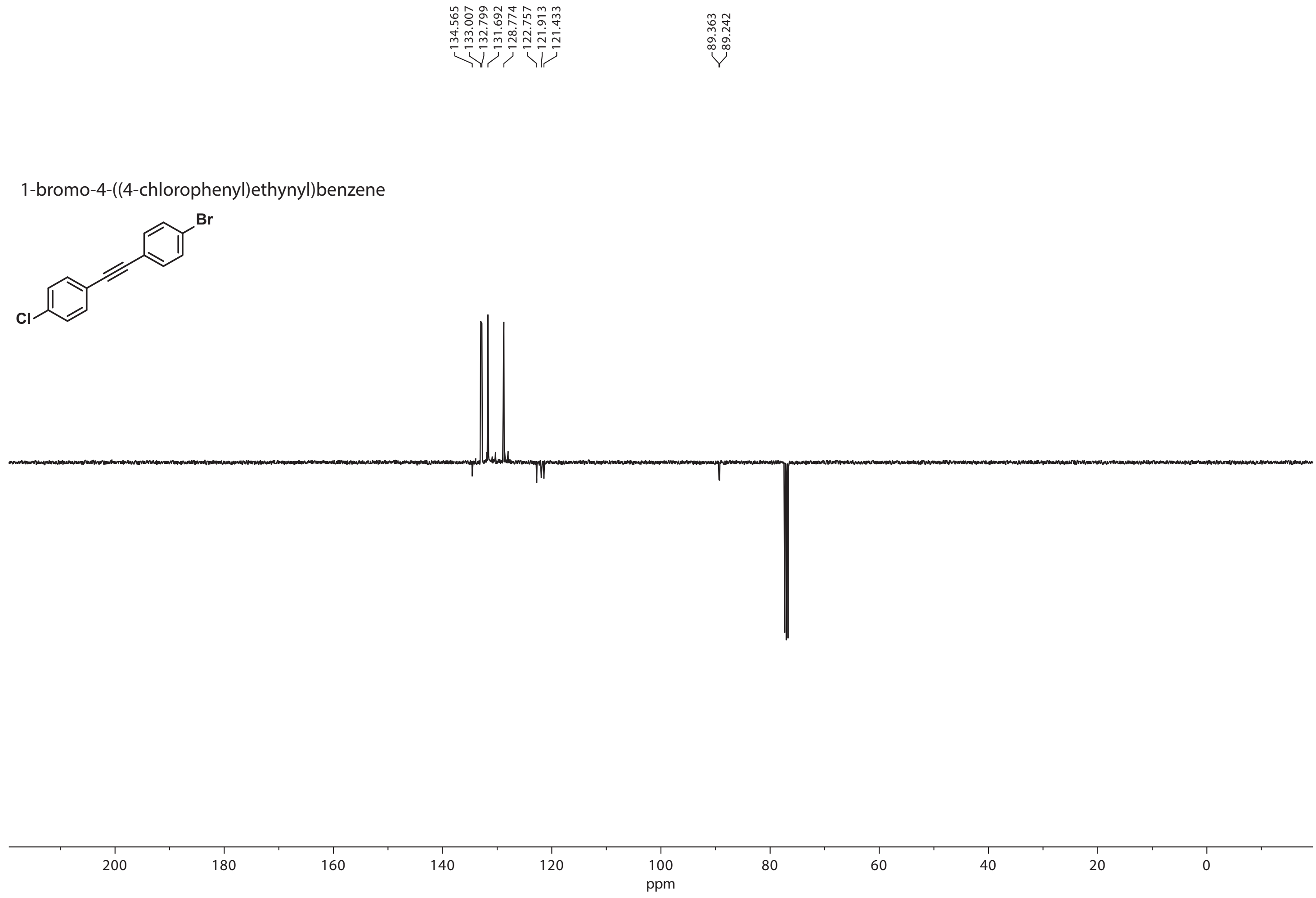

Figure S20. ^{1}H-NMR and ^{13}C-NMR of Ethyl 4-(p-tolylethynyl)benzoate (**3r**)

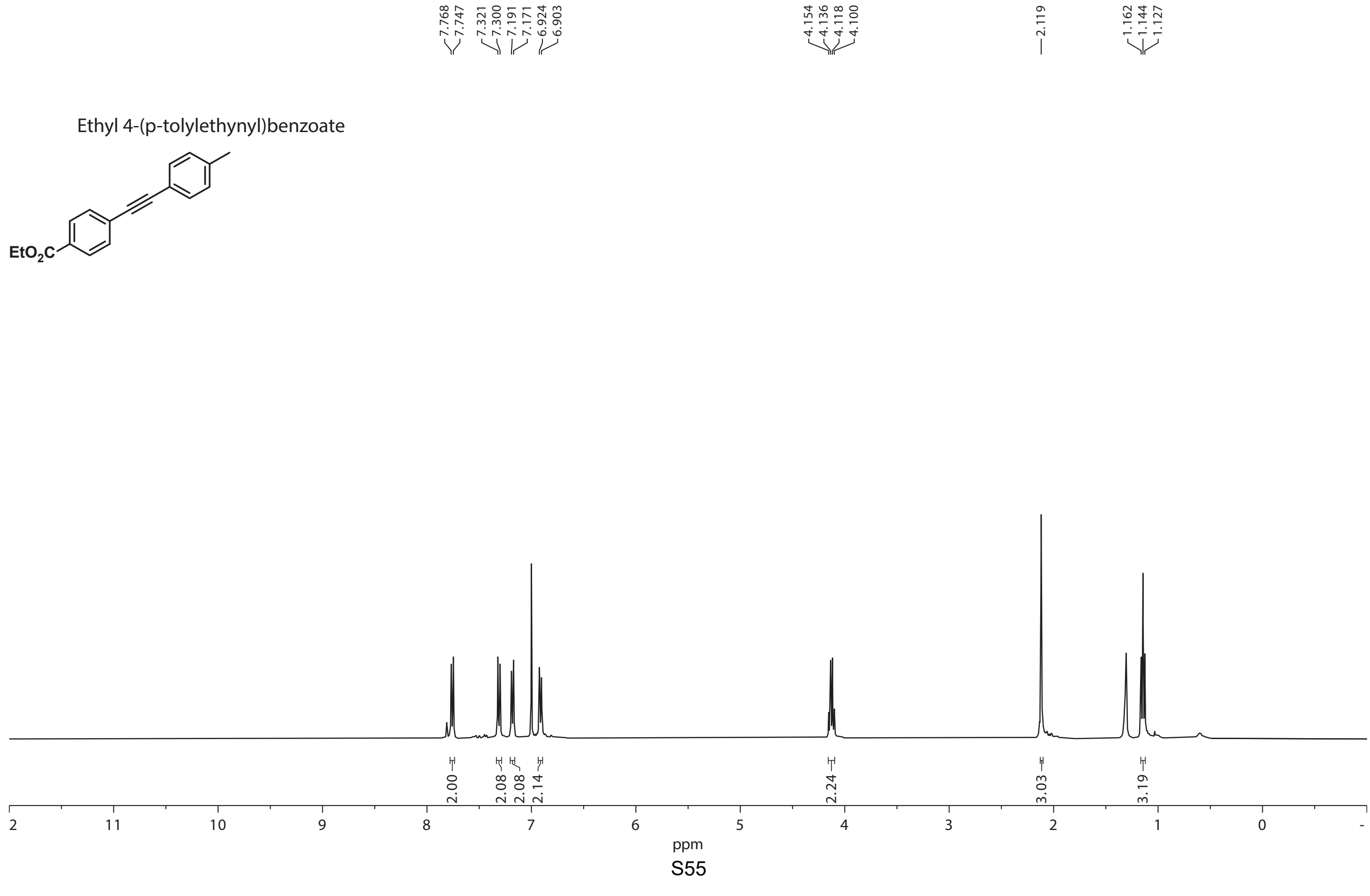

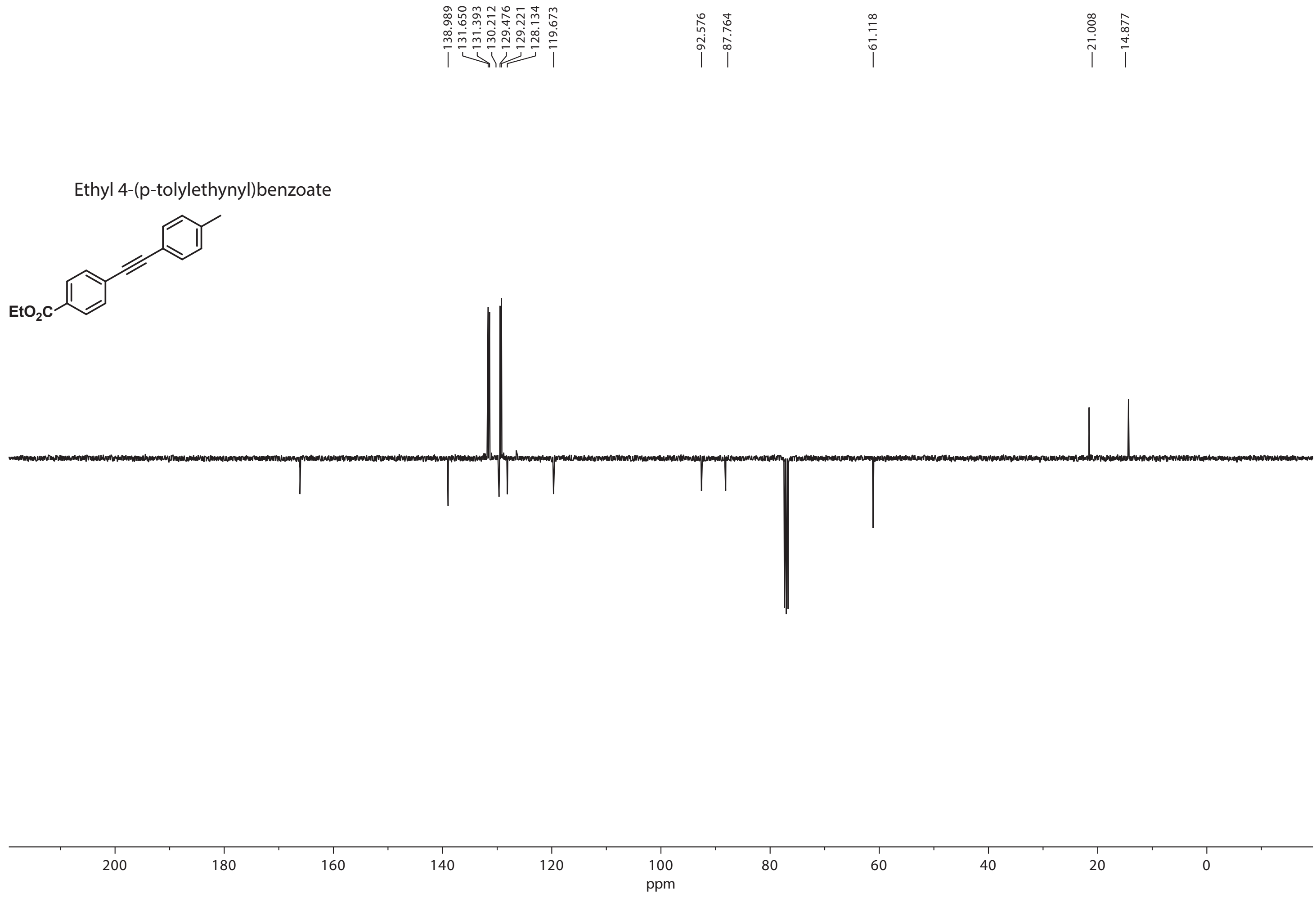

Figure S21. ^{1}H-NMR and ^{13}C-NMR of 1-chloro-4-((4-methoxyphenyl)ethynyl)benzene (**3s**)

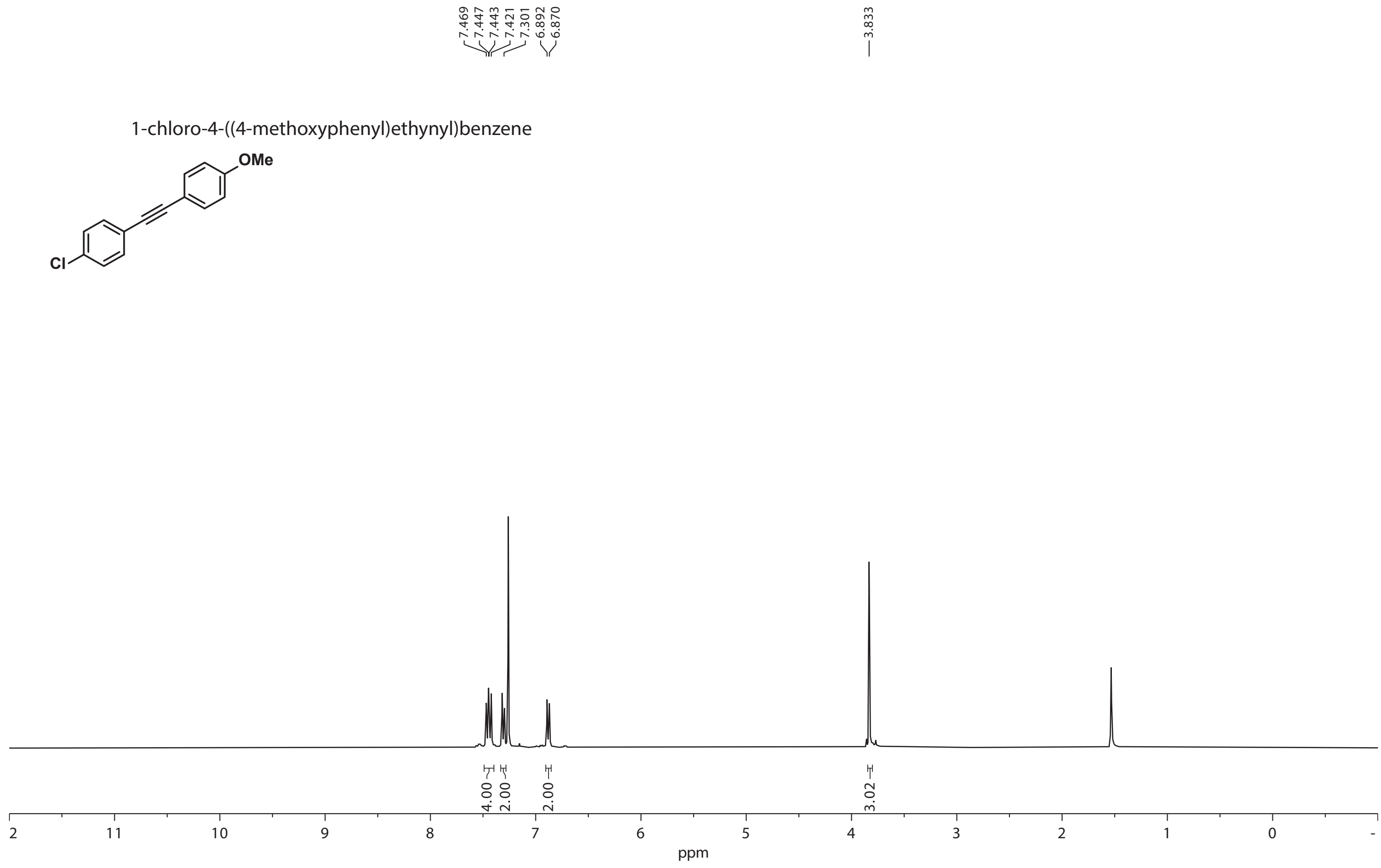

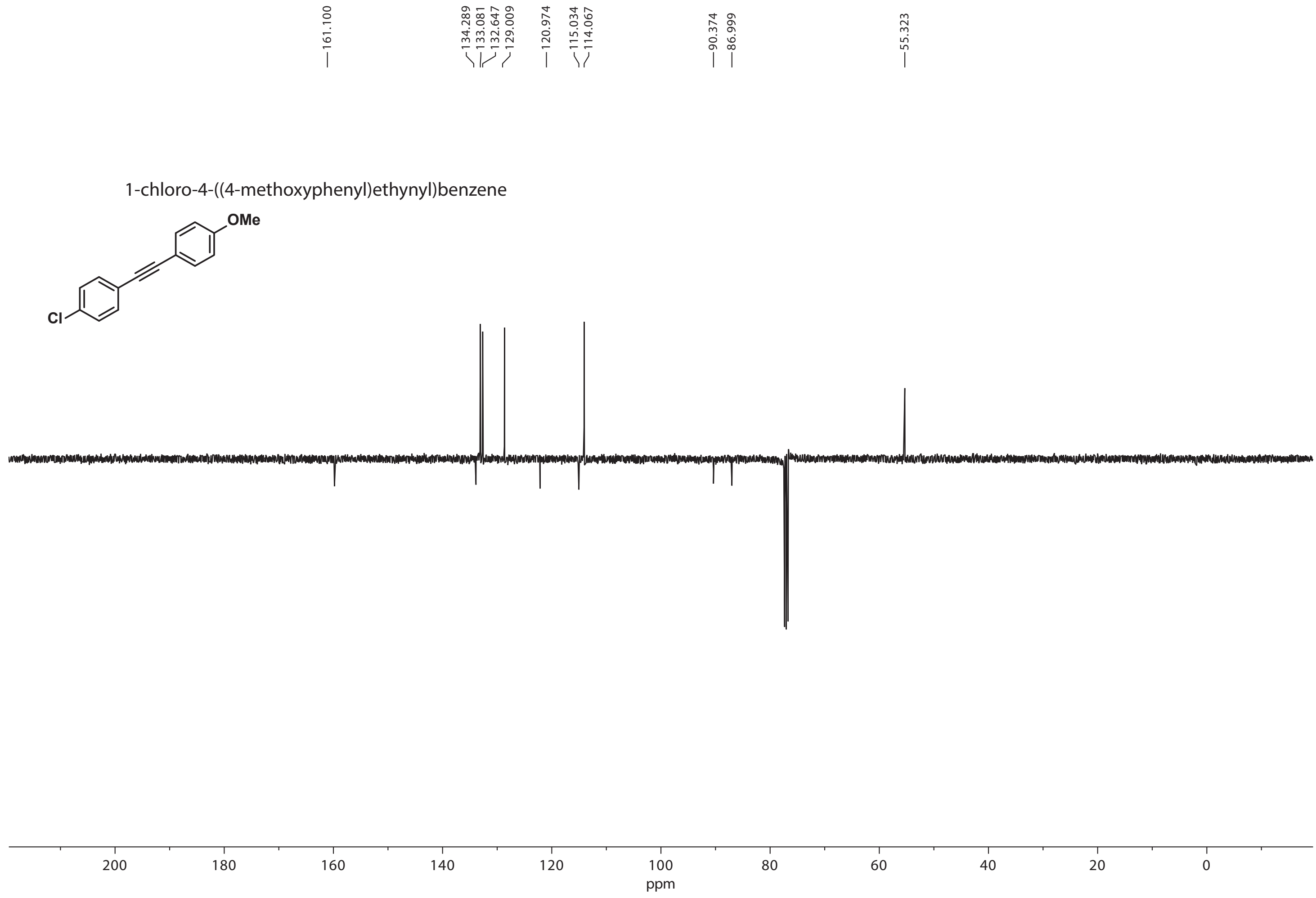

Figure S22. ^{1}H-NMR and ^{13}C-NMR of ((4-(tert-butyl)phenyl)ethynyl)triisopropylsilane (**3t**)

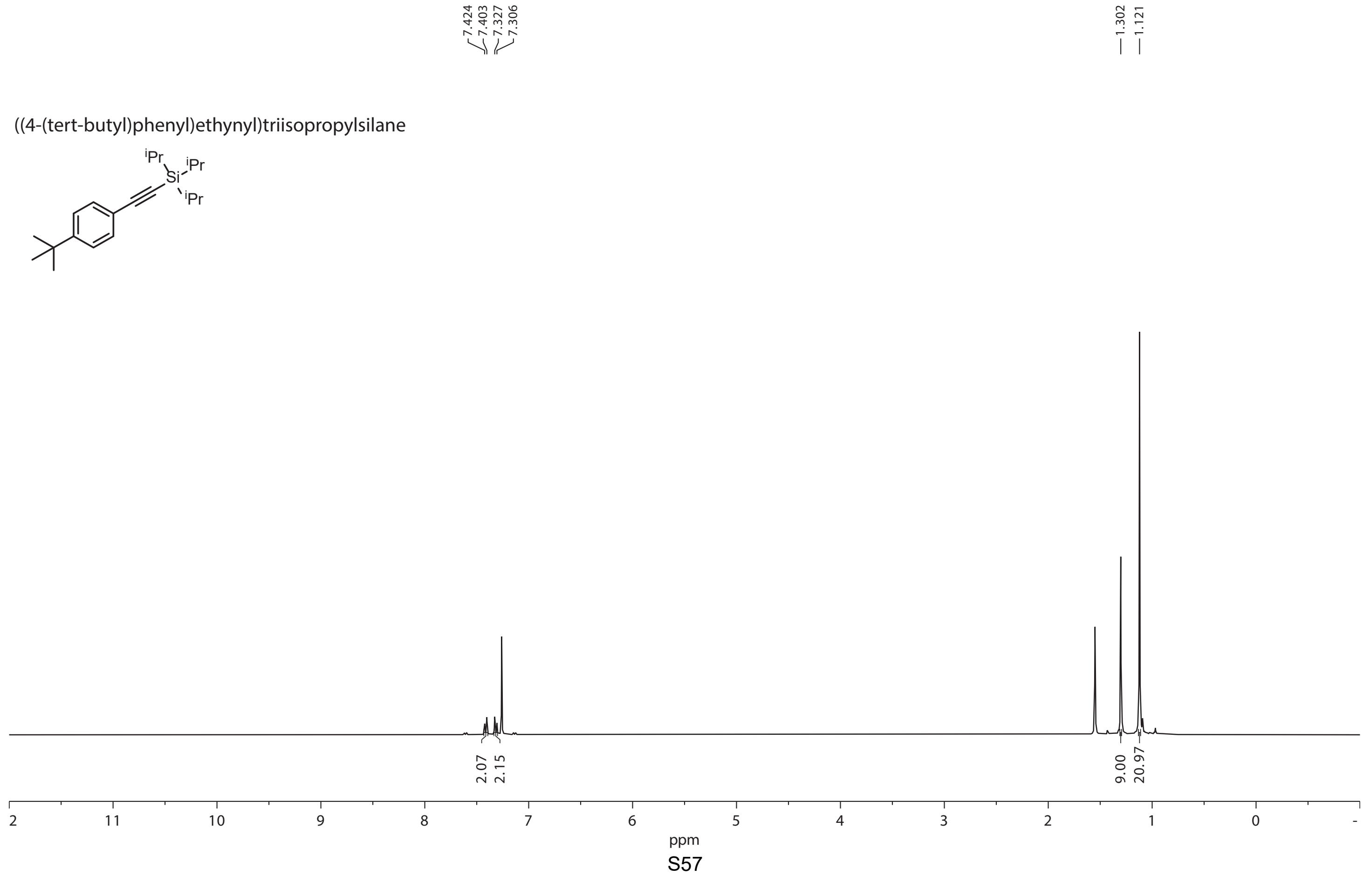

((4-(tert-butyl)phenyl)ethynyl)triisopropylsilane

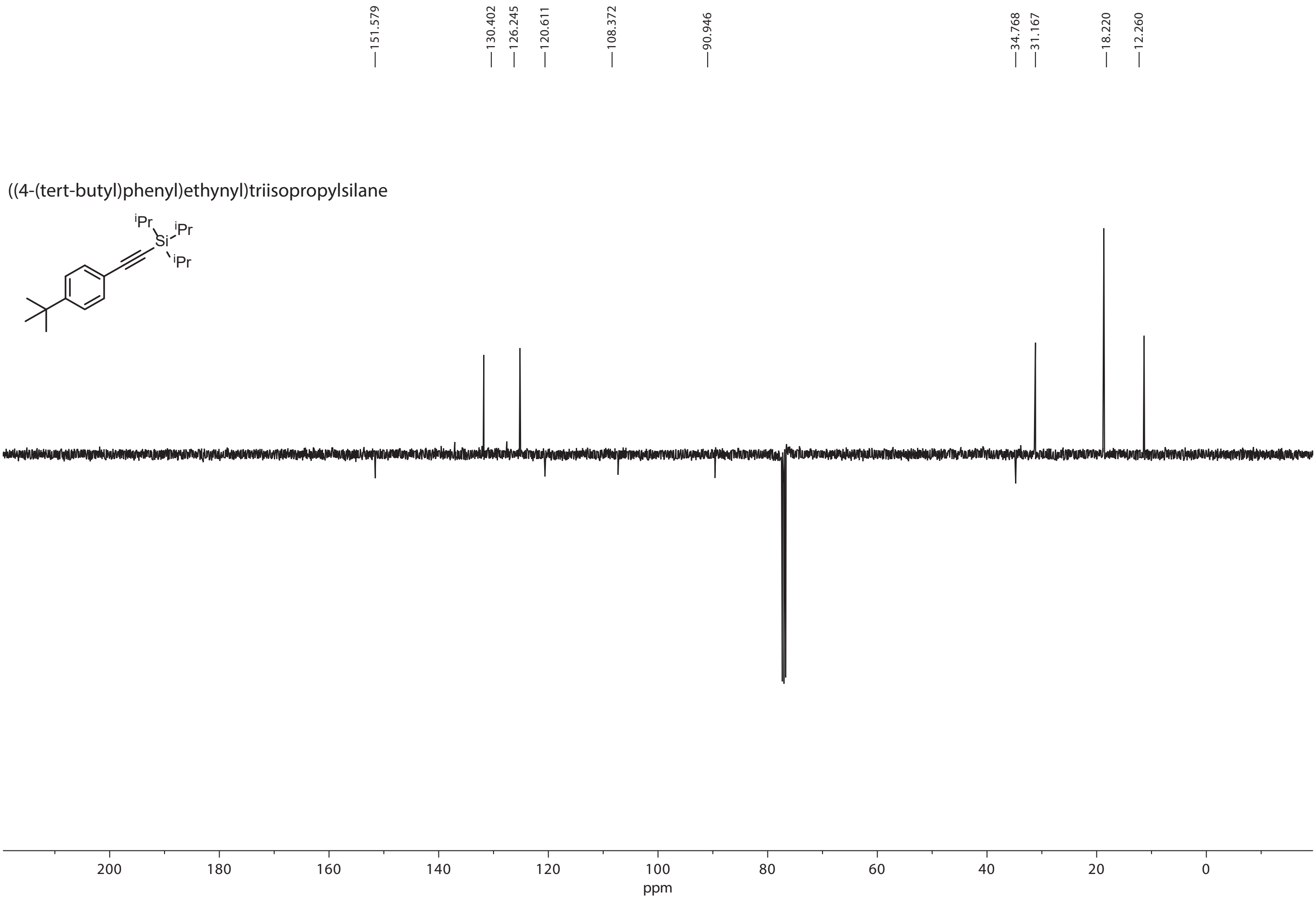

Figure S23. ^{1}H-NMR, 2D NMR spectra (^{1}H–^{1}H COSY and ^{1}H–^{1}H NOESY) and ^{13}C-NMR of (Z)-3-benzylidene-2-methylisoindolin-1-one (**5a**)

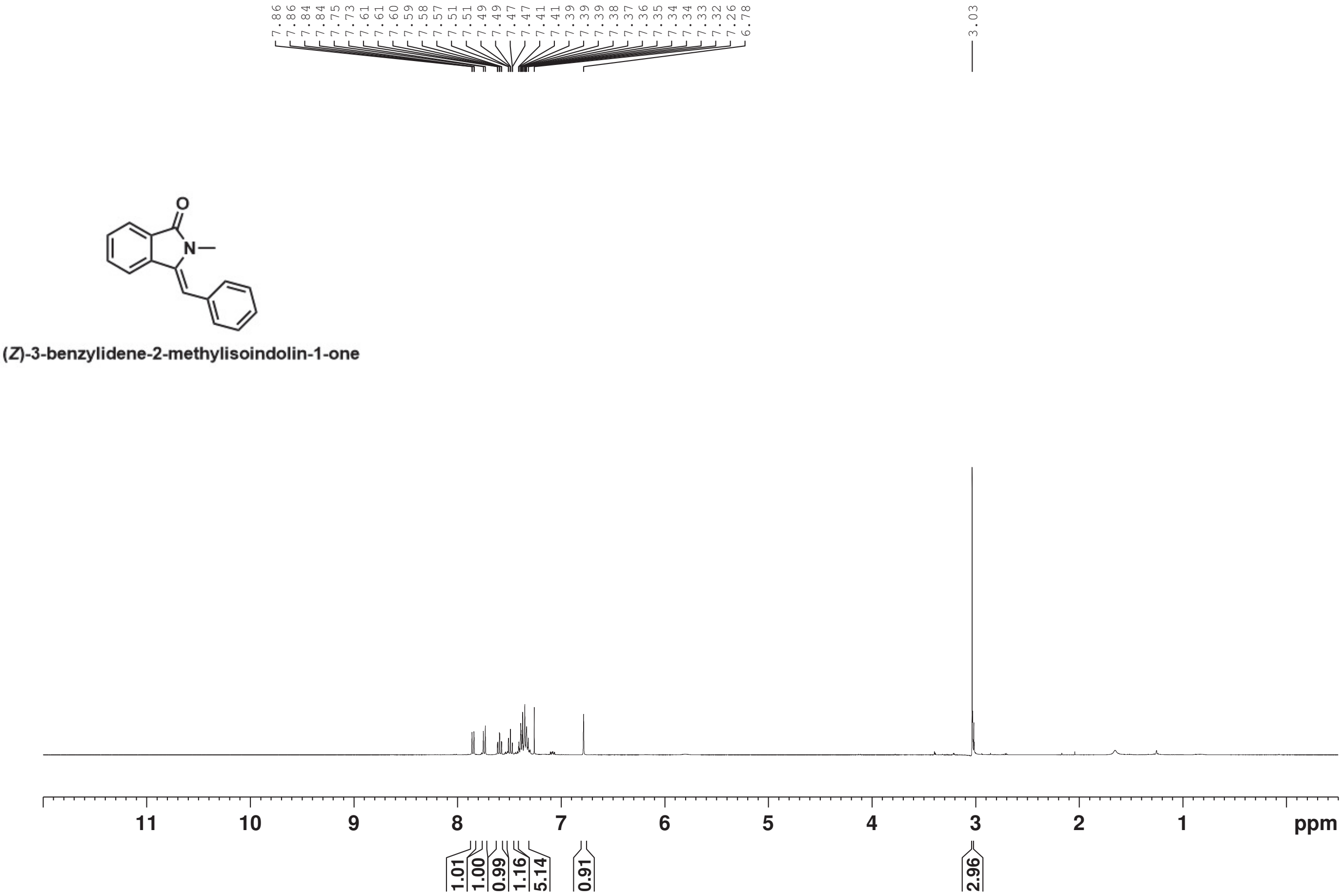

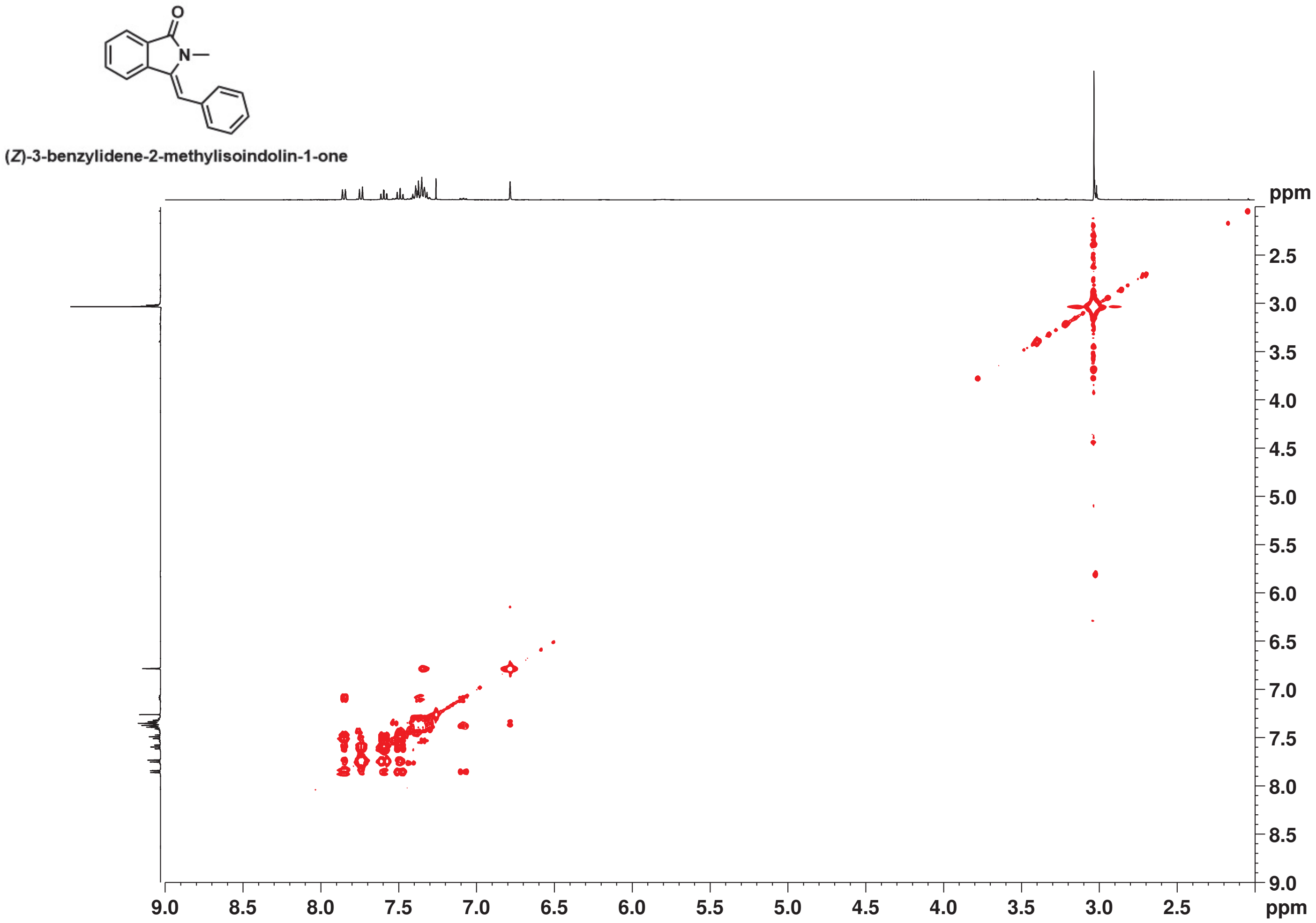

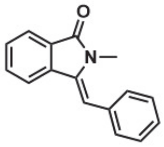

(*Z*)-3-benzylidene-2-methylisoindolin-1-one

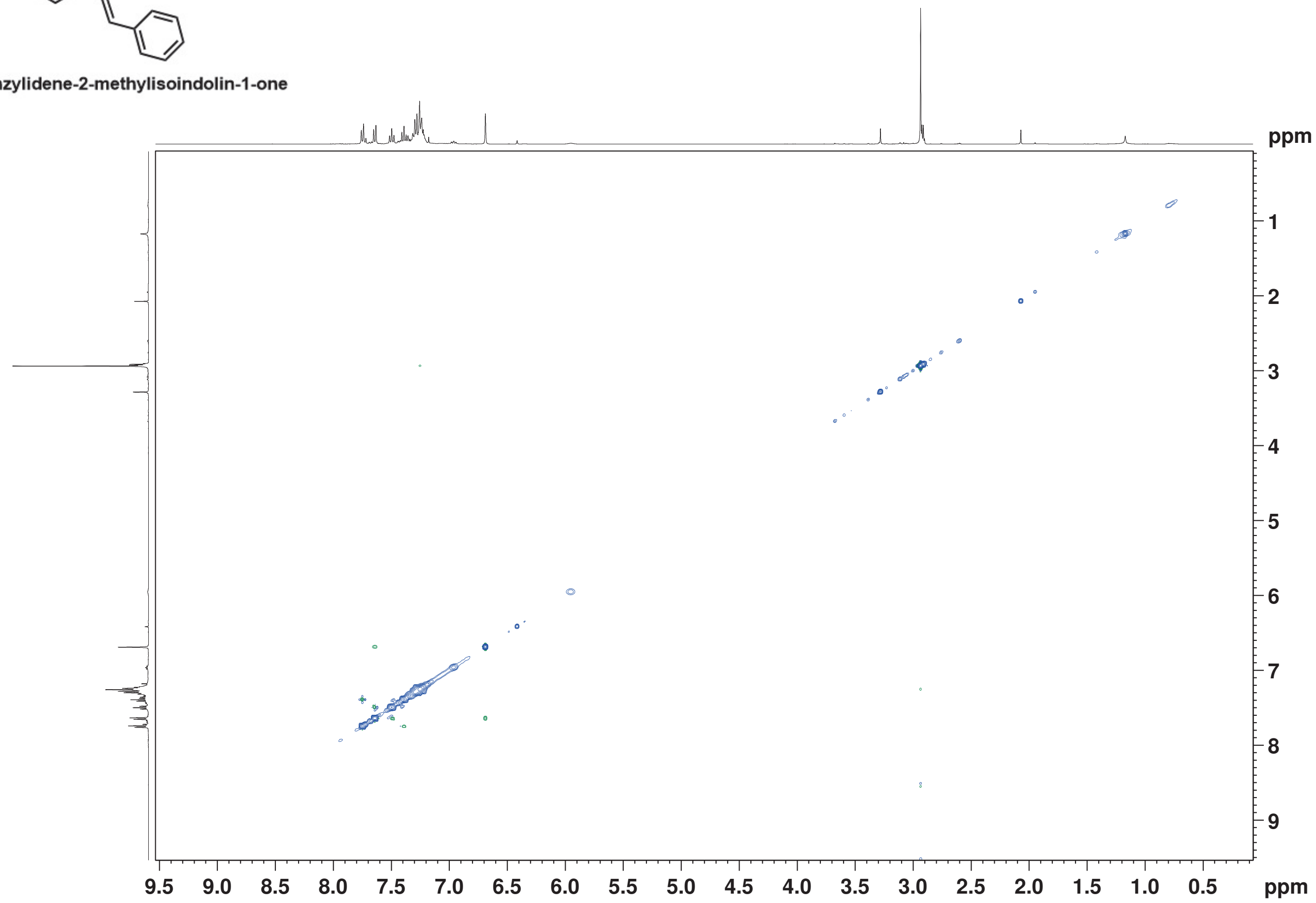

(*Z*)-3-benzylidene-2-methylisoindolin-1-one

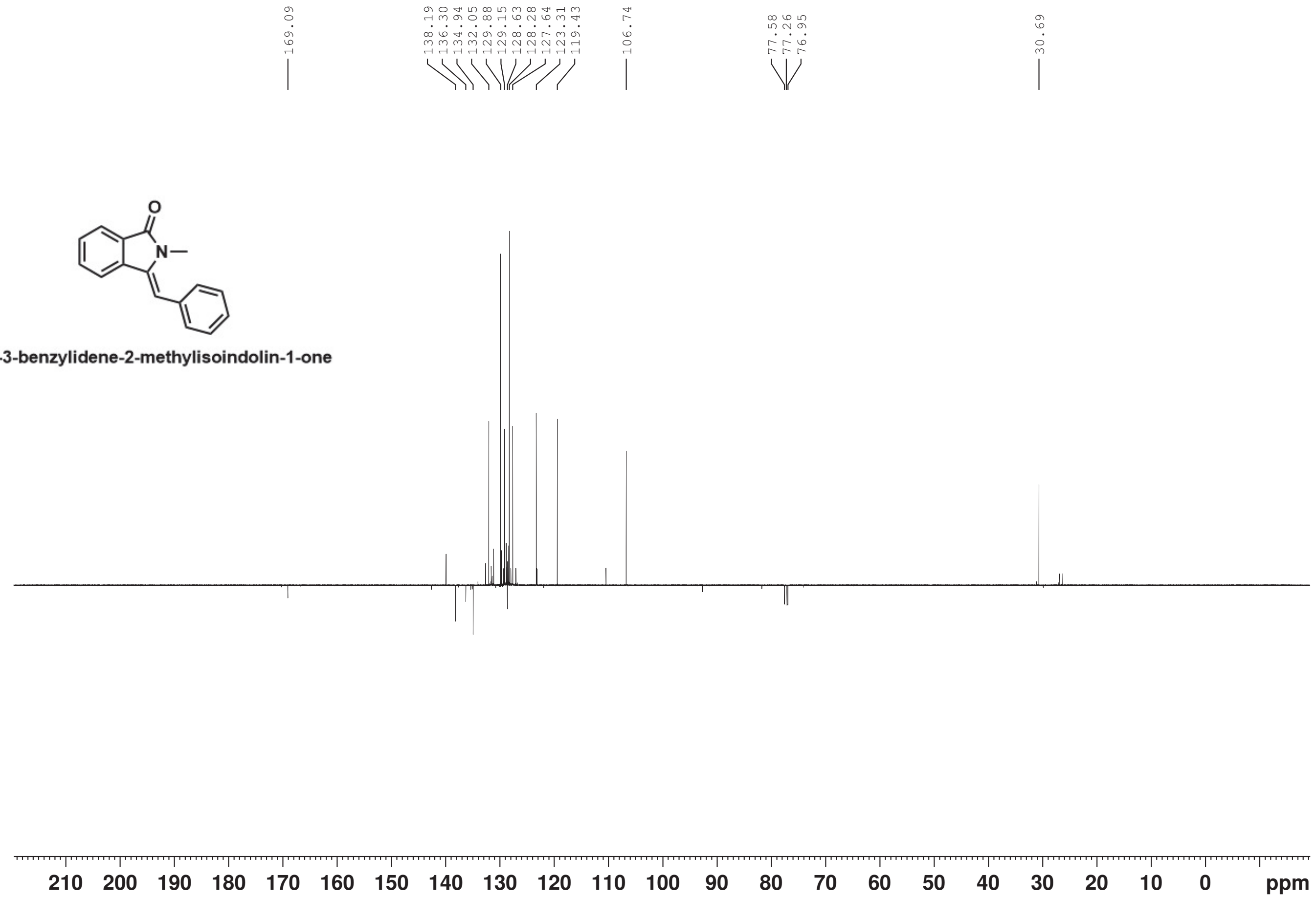

Supplement: Supplementary file 1 — Supplementary Material [file CSSC-19-e70827-s001.pdf]
